# A worldwide phylogeography of the whiteworm lichens *Thamnolia* reveals three lineages with distinct habitats and evolutionary histories

**DOI:** 10.1002/ece3.2917

**Published:** 2017-04-13

**Authors:** Ioana Onuţ‐Brännström, Leif Tibell, Hanna Johannesson

**Affiliations:** ^1^ Department of Systematic BiologyEvolutionary Biology CentreUppsala UniversityUppsalaSweden

**Keywords:** chemical variation, clonality, lichens, phylogeography, symbiosis, *Thamnolia*

## Abstract

*Thamnolia* is a lichenized fungus with an extremely wide distribution, being encountered in arctic and alpine environments in most continents. In this study, we used molecular markers to investigate the population structure of the fungal symbiont and the associated photosynthetic partner of *Thamnolia*. By analyzing molecular, morphological, and chemical variation among 253 specimens covering the species distribution range, we revealed the existence of three mycobiont lineages. One lineage (Lineage A) is confined to the tundra region of Siberia and the Aleutian Islands, a second (Lineage B) is found in the high alpine region of the Alps and the Carpathians Mountains, and a third (Lineage C) has a worldwide distribution and covers both the aforementioned ecosystems. Molecular dating analysis indicated that the split of the three lineages is older than the last glacial maximum, but the distribution ranges and the population genetic analyses suggest an influence of last glacial period on the present‐day population structure of each lineage. We found a very low diversity of Lineage B, but a higher and similar one in Lineages A and C. Demographic analyses suggested that Lineage C has its origin in the Northern Hemisphere, possibly Scandinavia, and that it has passed through a bottleneck followed by a recent population expansion. While all three lineages reproduce clonally, recombination tests suggest rare or past recombination in both Lineages A and C. Moreover, our data showed that Lineage C has a comparatively low photobiont specificity, being found associated with four widespread *Trebouxia* lineages (three of them also shared with other lichens), while Lineages A and B exclusively harbor *T. simplex* s. lat. Finally, we did not find support for the recognition of taxa in *Thamnolia* based on either morphological or chemical characters.

## 1 Introduction

Our understanding of how lichens colonize new habitats is still in its infancy, and studies including analyses of both main symbiotic partners are necessary to further understand their dispersal and ecological success. Phylogeographic studies of lichens based on molecular data have been few so far (e.g., Buschbom, 2007; Geml, Kauff, Brochmann, & Taylor, 2010; Högberg, Scott, Thor, & Taylor, 2002; Palice & Printzen, 2004; Printzen & Ekman, 2002; Printzen, Ekman, & Tonsberg, 2003; Sork & Werth, 2014; Werth & Sork, 2010; Widmer et al., 2012) but promise to bring important insights into factors such as postglacial migrations, which might have determined the current distribution of species (Printzen, 2008).

**Figure 1:**
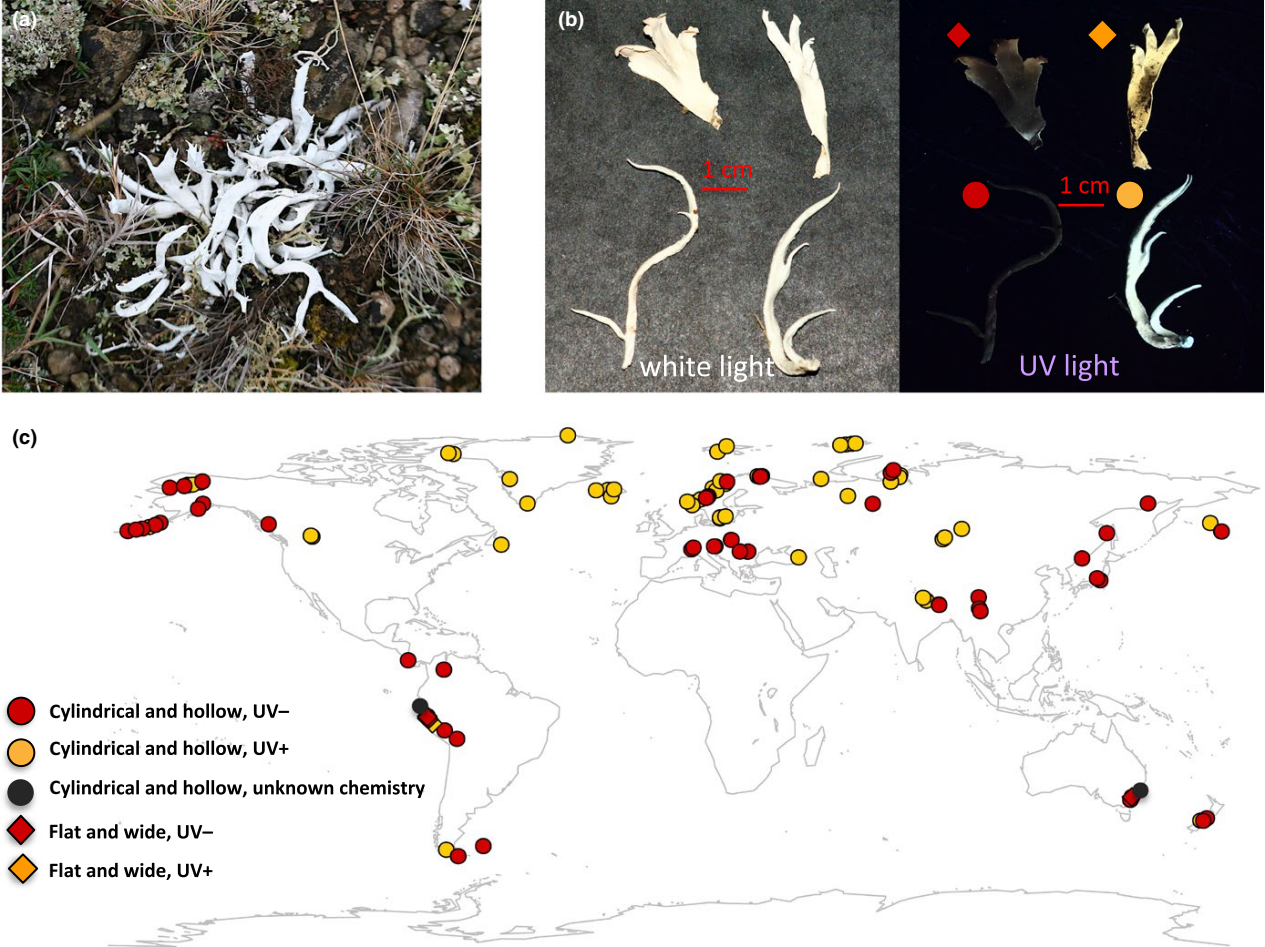
The distribution of and morphological and chemical variation in *Thamnolia*: (a) *Thamnolia* specimen from Öland (Sweden) growing in a typical habitat (thin layer of soil and pebbles covered by low grasses, mosses, and other lichen species). (b) The morphological (“flat and wide” phenotype is shown at the top and “cylindrical and hollow” phenotype is shown at the bottom) and chemical variation in *Thamnolia* seen under UV light. (c) Geographic distribution of *Thamnolia* used in this study. The samples with “cylindrical and hollow” phenotype are coded with 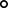 and the “flat and wide” ones with ♦. The chemical varieties are color‐coded: yellow for the UV + (squamatic and baeomycesic acids) and red for UV− (thamnolic acid); unknown chemistry is coded black

Here, we present a study of the phylogeography of *Thamnolia* Ach. ex Shaer., (Icmadophilaceae). These are truly enigmatic lichens, which frequently have attracted the attention of lichenologists due to their peculiar morphology, wide distribution range, apparent lack of sexual reproduction, and chemical variation. *Thamnolia* is often encountered in arctic and alpine tundra environments in all continents except for Africa and Antarctica (Sheard, 1977), and as the name suggests, it resembles small, chalky white worms that occur among low grass and mosses (Figure 1a). In this study, we refer to the lichens of the entire genus as whiteworm lichens. Except for occasional, and probably erroneous, reports of apothecia (fruiting bodies), the fungal symbiont (the mycobiont) seems to be sterile (Beyer, 2009). Sexual fungal spores, which are often carried long distances in the air, are thus unlikely to serve as dispersal propagules. In contrast, thallus fragmentation by lateral branches and longitudinal strips, containing an established, successful symbiotic partnership, has been assumed to be the main reproduction mode of *Thamnolia* (Andrei, Iacob, & Pascale, 2006–2007; Lord et al., 2013). It is unknown whether the fragments travel long distances by wind alone, but it has been suggested that birds or grazing animals enable long‐distance dispersal (Wright, 1992). It has also recently been shown that *Thamnolia* produces fungal mitospores (“conidia”; Lord et al., 2013). Their function is as of yet unknown, but they may act as male gametes during mating and/or serve in dispersal (Maheshwari, 1998; Tibell, 1993, 1997).

Previous attempts to understand the dispersal biology of *Thamnolia* have been inconclusive. A previous study using molecular markers could not reject the hypothesis of strict clonality in *Thamnolia,* even if weak signs of recombination were detected (Nelsen & Gargas, 2009a). The clonality of *Thamnolia* was also supported when microsatellite markers from several whiteworm populations from Manitoba (USA) were studied (Cassie & Piercey‐Normore, 2008), and significant population division and linkage disequilibrium were observed. However, these studies of the *Thamnolia* mycobiont were based on a limited dataset, which has called for additional studies of the species using a more comprehensive sampling. Furthermore, contrary to expectations of vertical transmission of the photosynthetic symbiont, *Thamnolia* was found to exhibit low photobiont specificity in being able to associate with different species of green algae of *Trebouxia* (Nelsen & Gargas, 2009b).

Another aspect of *Thamnolia* that has attracted the attention of biologists is its variation in secondary chemistry. It has thalli of two distinct, mutually exclusive chemotypes (i.e., entities with different composition of secondary metabolites, Nelsen & Gargas, 2009a): Thalli containing squamatic and baeomycesic acids [often named *T. vermicularis* ssp. *subuliformis* (Ehrh.) Schaer.] are encountered at higher frequency in the Northern Hemisphere, while those containing thamnolic acid [*T. vermicularis* ssp. *vermicularis* (Ehrh.) Schaer.] are more frequently found in the Southern Hemisphere (Sato, 1963). The same set of chemotypes was also reported to occur in *T. papelillo* R. Sant., a species distinguished from *T. vermicularis* in thallus morphology (Santesson, 2004). Several attempts have been made to taxonomically recognize some of the more distinctive phenotypes based on thallus chemistry (Culberson, 1963) or a combination of chemical and morphological features (Santesson, 2004; Figure 1; Table S1). A recent molecular phylogenetic analysis, using a limited sampling, showed that the two chemotypes of *Thamnolia* do not form monophyletic groups (Nelsen & Gargas, 2009a). However, the phylogenetic signal given by the chemistry in this study was weak, and thus, it has remained unclear whether the chemical differences are ontogenetically determined, or are triggered by other factors such as the environment.

The overall aim of this study was to increase our understanding of the demography and life history of *Thamnolia*, by investigating the population structure of its mycobiont, and the distribution of the symbiotic algal genotypes. By using phylogeographic analyses of 249 *Thamnolia* samples, we aimed at relating the role of Pleistocene events in the shaping of present‐day distributions of *Thamnolia* populations and the genetic variation between them. Finally, by studying samples of *Thamnolia* covering its phenotypic diversity, we aimed at evaluating the use of morphology and chemistry for the recognition of taxa in *Thamnolia*.

## 2 Materials and Methods

### 2.1 Sampling and phenotypic analyses

We analyzed sequence data from a total of 253 samples (Table S2). Of these, 250 samples belong to *Thamnolia*, and they represent its geographic range. Nine of these were referred to *T. papelillo*, which until recently (Santesson, 2004) was considered a variety of *T. vermicularis,* when *Thamnolia papelillo* was described as different from *T. vermicularis* in having flat and wide podetia (we henceforth use the term “podetium” for individual thalli; Table S1), and a distribution restricted to South America. Molecular data have so far not been available for *T. papelillo*, and its taxonomic status has been controversial. For the majority of the samples used in our investigation, we investigated field‐collected thallus material, but for three of them axenic fungal cultures obtained from the Akita Prefectural University, Japan, or the Institute of Plant Sciences, University of Graz, Austria (Table S2), were used. For 35 of the samples, sequences were retrieved from GenBank, and we also used data from draft fungal genomes of *Thamnolia sp*. and three additional lichens in Icmadophilaceae: *Dibaeis baeomyces* (L. f.) Rambold & Hertel, *Icmadophila ericetorum* (L.) Zahlbr., and *Siphula ceratites* (Wahlenb.) Fr. Information about the samples: sampling location, herbarium voucher, thallus chemistry and morphology, collector, and accession number of GenBank sequences, is given in Table S2.

Several samples were gathered from each locality. Whenever possible, the samples were collected along a transect and within a distance of 1–3 m from each other (Table S2). Only one podetium per sample was included in the analyses, unless two podetia of different chemotypes were encountered in the same sample, when both of them were studied. Each podetium was considered an individual, and each individual was assigned to one of two morphotypes: “cylindrical and hollow” *versus* “flat and wide,” using Santesson herbarium material as reference (Santesson, 2004). Thallus chemistry was recorded by documenting the fluorescence under UV light (3,500 A): The podetium appears bright yellow when containing squamatic and baeomycesic acids, and dark red when only containing thamnolic acid (Culberson, 1963; Table S1), and these chemotypes were here designated as “UV+” and “UV−,” respectively. For the 35 *Thamnolia* sequences downloaded from GenBank (Table S2), we were guided by the phenotype information given by the authors.

A map of the origin of all the *Thamnolia* used for this study (new and from GenBank) is presented in Figure 1c and on Google Maps. The following link enables the visualization of a map at high resolution (https://drive.google.com/open?id=1JHKT8Hcaozr3Dxr2BPviOdZWNP8&usp=sharing).

### 2.2 DNA extraction, PCR, and Sanger sequencing

Before DNA extraction, we visually examined each podetium for fungal parasites and selected only specimens seemingly free of infection. We placed each individual sample in a separate tube together with two tungsten (3 mm) carbide beads, froze them in liquid nitrogen, followed by two‐minute shaking at maximum speed in a Qiagen TissueLyser II machine. We subsequently isolated the total genomic DNA using the DNeasy Plant Mini Kit from Qiagen.

For the mycobiont, a total of six nuclear markers were analyzed: intergenic spacer region (IGS), internal transcribed spacer (ITS), β‐tubulin (β‐tub), translation elongation factor 1‐alpha gene (EFα), RNA polymerase II core subunit (RPB2), and a putative DEAD‐box helicase (DEAD, annotation based on blast results in NCBI, e.g., accession number XM_001242797). For the photobiont, we used three markers: two nuclear regions (ITS and actin) and one mitochondrial [cytochrome c oxidase subunit I (COX) (Table S3). For all PCR amplifications, we used the Phusion High‐Fidelity DNA Polymerase kit (ThermoFisherScientific). The primer combinations used for each marker are found in Table S3, and when we used more than one combination for a particular marker, the primer position is shown in Figure S1. We sequenced the PCR products from both directions using Sanger technology with an ABI3730xl. We trimmed and corrected the raw reads for errors using Geneious 9 (http://www.geneious.com).

### 2.3 Extracting sequences of the six fungal nuclear markers from four draft fungal genomes

Genomic data were obtained from draft fungal genomes of *Dibaeis baeomyces, Icmadophila ericetorum*,* Siphula ceratites,* and *Thamnolia sp*. (Table S2). The details on the assembly of these genome sequences will be reported on elsewhere. The draft genomes of the axenic fungal cultures were used as a source to verify that the PCR‐amplified sequences obtained from thalli originate from the main mycobiont (Table S3), and to extract orthologous sequences for the outgroups. To find the homologous sequences for each of our loci, we used a BLAST search strategy employing the BLAST 2.2.31+ suit (Camacho et al., 2009). *Thamnolia* sequences of our six fungal loci of interest were BLASTed against the scaffolds of the draft assemblies using blastn for ITS and IGS, and tblastn for EFα, β‐tub, DEAD, and RPB2. We considered only hits with e‐values smaller than 1e‐5, and determined orthology by a reciprocal best blast hit to the *Thamnolia* draft genome. We aligned the candidate sequences to the sequences from *Thamnolia* species generated in this study using Mafft version 7 (Katoh, 2013) and visualized them in AliView v.1.18‐beta7 (Larsson, 2014). The sequences that were homologous to our loci of interest were used for further analyses.

### 2.4 Retrieving algal sequences from GenBank

To assign the algal sequences from our lichen specimens to an algal species/lineage, we downloaded a total of 91 *Trebouxia* ITS sequences from GenBank. These sequences were obtained through a blastn search using the newly generated algal sequences from our lichen specimens as queries. We considered only hits that had an e‐value of 0 and were sequenced from other sources than *Thamnolia* (Table S4).

### 2.5 Sequence alignment and phylogenetic analyses

For each analysis, we aligned the included sequences with Mafft version 7 and visualized the alignment in AliView v.1.18‐beta7. We manually removed the ambiguous sites in each alignment. When we used data from multiple markers in the analysis, the sequences were concatenated. We deposited all the alignments in the Dryad repository (https://doi.org/10.5061/dryad.79d91).

For both fungal and algal datasets, we performed maximum‐likelihood (ML) inference to elucidate phylogenetic relationships. We chose the nucleotide substitutions models under the corrected Akaike information criterion using JModelTest v 2.1.6 (Darriba, Taboada, Doallo, & Posada, 2012; Table S5), and performed the ML phylogenetic analyses using Garli v 2.0 (Bazinet, Zwicki, & Cummings, 2014). In the case of concatenated datasets, we applied the substitution model for each individual locus. We performed five independent searches to identify the tree with the best likelihood value, and calculated the branch support for each phylogeny by performing 500 bootstrap replicates in Garli v 2.0. Prior to concatenation, we visually inspected individual gene trees and verified absence of significant discordance. Additionally, we inferred NeighbourNet and maximum‐parsimony haplotype networks for fungal datasets using SplitsTree (Huson & Bryant, 2006) and TCS v 1.21 (Clement, Posada, & Crandall, 2000), respectively. For the latter, we transformed the fasta files to TCS input files using the online tool FaBox (Villesen, 2007). Gaps were treated as missing data.

### 2.6 Polymorphism and divergence estimates of the mycobiont

In order to characterize the levels of genetic diversity within, and differentiation between *Thamnolia* phylogenetic lineages, we used several estimates of genetic variability and differentiation for all fungal datasets (https://doi.org/10.5061/dryad.79d91). First, we calculated the genotypic diversity (Gdiv, Caron, Ede, & Sunnucks, 2014) of each lineage by dividing the number of genotypes (in our case the number of sequences after clone correction) by the total number of genotyped individuals (the total number of sequences without clone correction). Second, we used R package Pegas v 0.9 (Paradis, 2010) to calculate the nucleotide diversity (π, Nei, 1987) within each lineage, with and without clone correction, as a measure of the degree of polymorphism. We used both bootstrapping (sampling with replacement) and permutation with randomization (with sample size of 2) to obtain the 95% confidence intervals. For each analysis, we performed 1,000 permutations. Third, we used DNASP v.5 (Librado & Rozas, 2009) to calculate Tajima's *D* and Fu's *F* statistics on the clone‐corrected fungal datasets. Forth, after clone correction, we estimated using Mega 6.06 (Tamura, Stecher, Peterson, Filipski, & Kumar, 2013) the net mean sequence distance between lineages (*D*_A_) within two standard errors (SE) confidence intervals. The *D*_A_ was calculated as a function of average distances between lineages (*D*_xy_) and the sum of mean distance within lineages (*D*_x_ and *D*_y_) (*D*_A_ **= *D*_xy_ − (*D*_x_ + *D*_y_)/2). The matrix of *D*_A_ plus SE was obtained in Mega 6.06 using the Tamura–Nei model of evolution and 1,000 bootstrap replicates.

The *D*_A_ estimate of molecular divergence together with mutation rate estimates of the ITS region (μ) was used to calculate an approximate divergence time (*D*_T_) for the lineages as *D*_T_ (years) = *D*_A_ (subs/sites)/(μ × 2). We used three previously estimated ITS mutation rates quantified as substitution/site/year (s/s/y). The first two rates were calculated from two lichens in the class Lecanoromycetes (in which also *Thamnolia* is placed): *Melanelixia* (2.43 × 10−9 s/s/y) and *Oropogon* (2.38 × 10−9 s/s/y) (Leavitt, Esslinger, Divakar, & Lumbsch, 2012; Leavitt, Esslinger, & Lumbsch, 2012). The third rate was calculated for *Erysiphales* (Leotiomycetes) (2.52 × 10−9 s/s/y) based on phylogenetic estimates and node dating (Takamatsu & Matsuda, 2004). The assumption made for this study is that that the substitution rates of ITS region per year are similar across *Thamnolia, Melanelixia, Oropogon,* and *Erysiphales*.

### 2.7 Detection of recombination in the mycobiont

We searched for population‐level signs of recombination in *Thamnolia* populations using the four gamete test (4GT) together with the minimum number of recombination events (*R*_m_) (Hudson & Kaplan, 1985), and the Index of Association (*I*_A_) for all fungal datasets (https://doi.org/10.5061/dryad.79d91). The 4GT method searches for the presence of all four possible combinations of alleles in sequence data and is therefore sensitive to rare recombination events. The *I*_A_ method quantifies the genetic levels of linkage disequilibrium (LD) between variable sites (Avise & Wollenberg, 1997). *I*_A_ values close to 0 show linkage equilibrium (LE) and thus suggest molecular recombination, while values close to 1 suggest that the population is undergoing clonal reproduction. The 4GT and *R*_m_ analyses were performed hierarchically on different fungal datasets, among and between lineages, using DNASP v.5 (Librado & Rozas, 2009). For the *I*_A_ analyses, only the multilocus fungal datasets were used (https://doi.org/10.5061/dryad.79d91) and the tests were performed within and between lineages. For this analysis, the nucleotide sequences were transformed as multilocus genotypes (MLG) with the R package apex v. 1.0.2 (Thibaut, Kamvar, Schliep, Archer, & Harris, 2016) and the two indexes of association *I*_A_ and *r*_D_ (which accounts for number of loci sampled) were calculated in R with the package poppr v.2.3.0 (Kamvar, Brooks, & Grünwald, 2015).

## 3 Results

### 3.1 Phenotypic characterization of the samples

Of the 250 investigated *Thamnolia* specimens, 109 samples had cylindrical and hollow UV‐ thalli (traditionally often named “*T. vermicularis* ssp*. vermicularis*,” Table S1); 127 samples had cylindrical and hollow UV+ thalli (often referred to as “*T. vermicularis* ssp*. subuliformis”*); six samples had flat and wide UV‐ thallus (*T. papelillo* var*. papelillo*); and three samples had a flat and wide UV+ thallus (*T. papelillo* var*. subsolida*). For five samples, the morphology and chemistry was not known (i.e., the information was not recorded prior to DNA extraction, or absent in GenBank accessions) and they were assigned to the “cylindrical and hollow” morphology (Tables S1 and S2).

### 3.2 Mycobiont and photobiont sequences of Thamnolia

We successfully sequenced the fungal and algal loci for each sample (Table S6) except for two (Nepal_Dolpo_349 and Sweden_Tärna_219.2). In these two latter samples, rerunning a PCR under optimized conditions revealed sequences of two different algal ITS variants. By the blast strategy outlined above, we retrieved sequences for the six nuclear markers (ITS, IGS, β‐tub, DEAD, EFα, and RPB2) from the draft fungal genome assemblies of *D. baeomyces, I. ericetorum*,* S. ceratites,* and *Thamnolia sp*. All newly generated sequences of this study have been deposited in GenBank under the accession numbers listed in Table S6. We did not gather information from all markers from all specimens, and thus, for subsequent analyses, we combined all the information obtained from the sequences into four fungal datasets (Dataset F1–F4) and two algal datasets (A1 and A2). Dataset F1 contained a total of 249 ITS1 fungal sequences obtained from *Thamnolia* of all morphologies and chemical variation. Datasets F2, F3, and F4 subsets represent samples of dataset F1 but with more genetic markers. Dataset F2 was assembled from 154 full ITS sequences amplified from *Thamnolia* of all morphologies and chemical variation. Dataset F3 was prepared from 129 sequences amplified from both chemical variations of the “cylindrical and hollow” phenotype and is containing information from three nuclear markers (ITS, EFα, and DEAD). Dataset F4 contained fungal sequences for six nuclear markers (β‐tub, DEAD, EFα, IGS, ITS, and RPB2) amplified from 50 samples of both chemical variations of the “cylindrical and hollow” phenotype together with homologous sequences obtained through blast searches from the draft fungal genomes of *D. baeomyces, I. ericetorum, S. ceratites,* and *Thamnolia sp*. Dataset A1 comprised 214 ITS algal sequences obtained in our study together with sequences downloaded from GenBank. Dataset A2 contained a subset of 73 algal sequences generated for this study with genetic information from two nuclear markers (ITS and actin) and one mitochondrial locus (COX). Information for each dataset, including number of samples, markers, sequence length, and number of phylogenetically informative characters, is summarized in Table S5.

### 3.3 No support for recognition of taxa in Thamnolia based on morphological and chemical characters

**Figure 2:**
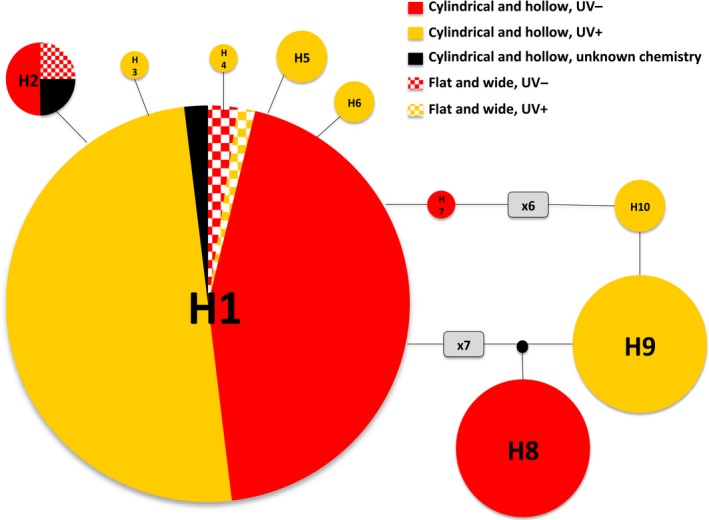
TCS haplotype network based on the ITS1 sequences of 249 *Thamnolia* samples across its distribution range. The data are clustered in 10 haplotypes and the distribution of samples of chemical and morphological variants is color‐coded

**Figure 3:**
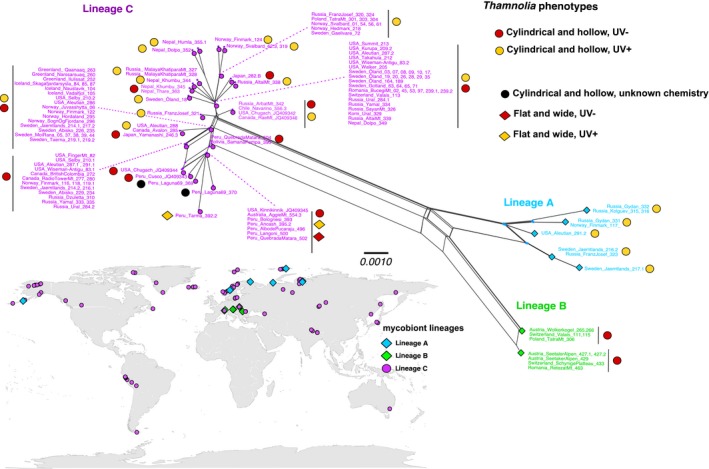
SplitsTree haplotype network of the ITS sequence data of 154 samples of *Thamnolia* from both hemispheres. Symbols show samples of Lineages A (blue), B (green), and C (purple) and their morphology and chemistry. The distribution of the three lineages shows Lineage A to be restricted to northern Europe, Kolguev Island, Gydan Peninsula, Franz Josef Land, and the Aleutian Islands; Lineage B being restricted to Central Europe; and Lineage C to have a wide distribution. The scale bar indicates the branch lengths. Thallus features (chemistry and morphology) are indicated for each haplotype as colored • and ♦

The traditional separation of *Thamnolia* into different taxa using morphological and/or chemical characters was not supported by our analyses. First, the TCS haplotype network based on sequence data from the ITS1 region from 249 *Thamnolia* samples from across its distribution range (Dataset F1; Table S5) showed that samples of both morphotypes and chemotypes from across the world belong to a single haplotype (Haplotype H1; Figure 2, Table S7). In addition, haplotype H2 (Table S7), only found in Australia, includes specimens of both morphotypes. The increase in genetic information used in the SplitsTree haplotype network based on the complete ITS region of 154 sequences of *Thamnolia* samples from both hemispheres (Dataset F2; Table S5) still maintained samples of the two morphotypes and chemotypes in the same haplotype (Figure 3 and Figure S2).

### 3.4 Three mycobiont lineages and their diversification

Our analyses revealed three, hitherto not observed, genetically distinct lineages of *Thamnolia*. Hereafter, we refer to them as Lineages A, B, and C. First, the SplitsTree haplotype network based on the complete ITS sequences of 154 samples of *Thamnolia* obtained from both hemispheres (Dataset F2; Table S5) revealed a structure with Lineages A and B to be more genetically similar than each of them to Lineage C (Figure 3). In all lineages, we found little reticulation. Lineage A included five haplotypes and comprised samples with cylindrical and hollow UV+ thalli; in Lineage B, we found two haplotypes, both being of the cylindrical and hollow morphotype and the UV− chemotype; in Lineage C, haplotypes of both morphotypes and both chemotypes were found (Figure 3). The TCS analysis of the same dataset revealed the same general pattern as the SplitsTree haplotype network, and a star‐like topology of the large haplogroups of Lineage C (Figure S3). Furthermore, the TCS analysis inferred the Northern Hemisphere haplotype H1a (Table S7), from Lineage C, to be ancestral (Figure S3). The TCS haplotype network also supports the separation of Lineage C from the Lineages A and B, but in this analysis Lineage B directly descends from one of the internal nodes of Lineage A (Figure S3).

The TCS haplotype network constructed from the concatenated data of three genes (ITS, IGS, and EFα) from 129 *Thamnolia* samples (all of cylindrical and hollow morphotype and both chemistries) from the Northern Hemisphere (Dataset F3; Table S5) showed greater resolution than when using ITS only, with regard to the genotypes of Lineage C (Figure S4). The designation of the ancestral haplotype was still maintained within Lineage C, but was narrowed down to a sample from Norway (haplotype H1a1; Figure S4). When comparing the results from this three‐gene dataset from the Northern Hemisphere with the network built from ITS sequences of samples across its distribution range (Dataset F2; Table S5; Figure S3), the star‐like topology of Lineage C was noticeable in both analyses, but more finely resolved when more sequence information was used. In the latter analysis, several haplotypes with samples from the same locality or region were grouped around a larger haplogroup (e.g., H1b2 of Figure S4), suggesting local diversification. Furthermore, by using more sequence information, we revealed additional steps between the three lineages, while the number of haplotypes in the two Lineages A and B did not increase (Figure S4).

In terms of geographic distribution, Lineage C was encountered in almost all investigated localities, while Lineages A and B were found to be mutually allopatric and geographically restricted to limited regions of the Northern Hemisphere (Table S7). Lineage A was found in high latitudes of the arctic tundra in Eurasia and also in the Aleutian Islands. With the exception of Kolguev Islands and Gydan Peninsula, it was found sympatric with Lineage C. Lineage B was found to be restricted to the high alpine region of the Alps and the Western Carpathian Mountains in Central Europe. Lineages B and C were found sympatric with the exception of the two investigated localities of Switzerland, in which only Lineage B was found (Figure 3).

Taken together, all datasets support the existence of three lineages in *Thamnolia*. Lineage C has a wide geographic distribution and contains all observed combinations of morphology and chemistry, while the Central European Lineage B and the circumpolar Lineage A have different chemistries.

### 3.5 A rooted phylogeny supports three monophyletic lineages in Thamnolia

**Figure 4:**
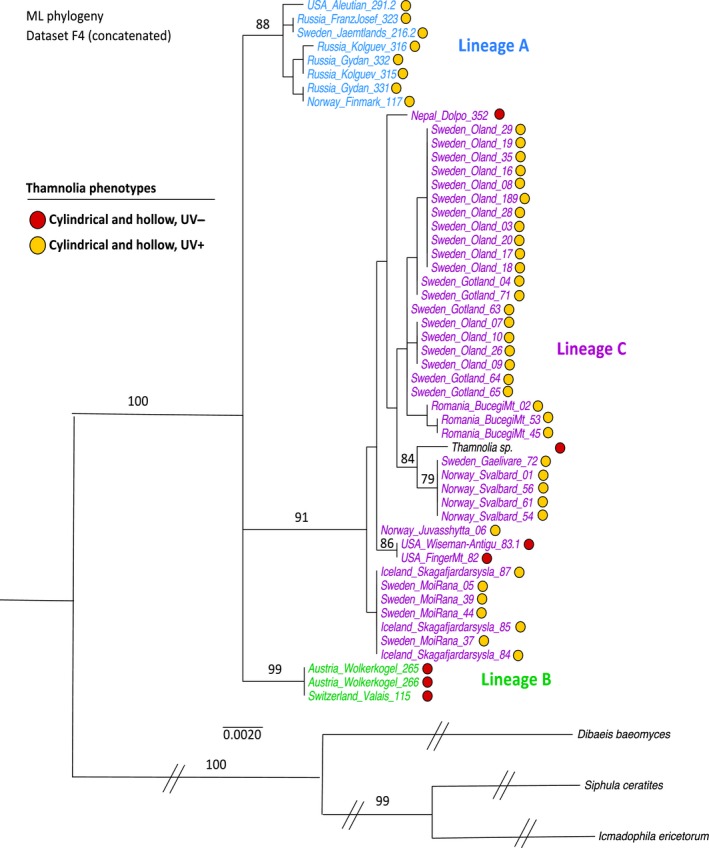
ML phylogeny of *Thamnolia* obtained by using sequence data from six concatenated genes (β‐tub, DEAD, EFα, ITS, IGS, and RPB2), and rooted with *D. baeomyces, I. ericetorum*, and *S. ceratites*. The IDs of the samples show the provenience and are colored according to their lineage belonging in the unrooted haplotypes networks: Lineages A (blue), B (green), and C (purple). The samples from which we used genetic information from the draft genomes are colored in black. Bootstrap values above 70% are indicated above branches. The scale bar indicates the branch lengths

Maximum‐likelihood phylogenies were based on both concatenated and individual datasets of sequences of six nuclear markers from 54 samples (Dataset F4; Table S5) rooted with *D. baeomyces, I. ericetorum,* and *S. ceratites*. In these analyses, the sequences of the 51 samples of *Thamnolia* were derived exclusively from the Northern Hemisphere. This analysis shows that *Thamnolia* forms a monophyletic group that includes three well‐supported clades (Lineages A, B, and C; Figure 4). The relationships between the three lineages were, however, not resolved.

Each single marker ML phylogeny (Figure S5) showed that all included *Thamnolia* sequences belong to a fully supported ingroup. Relative to the outgroup, each of the six nuclear markers sequenced from *Thamnolia* showed a very low amount of variation (excepting ITS; alignment available from the Dryad Digital Repository: https://doi.org/10.5061/dryad.79d91) and does not in the same analysis support all three lineages: The EFα gene tree shows Lineages A and B grouped together; Lineage B is strongly supported in β‐tub and DEAD phylogenies; and Lineage C is fully supported in the IGS tree (Figure S5).

### 3.6 Genetic diversity of Lineages A, B, and C

Based on the genetic diversity estimates, we found that Lineage C and Lineage B contained the highest proportion of clones, while Lineage A exhibited a relatively low number of identical sequences (Table 1, Gdiv measurements). Regarding the bootstrapped nucleotide diversity measurements, Lineages A and C showed a pronouncedly higher genetic polymorphism than Lineage B in all corrected and also not clone‐corrected datasets (Table 1, π values). The π values were maintained even when two samples were randomly redrawn 1,000 times showing robustness of our data for sample size (data not shown). In addition, using the genetic information based on the complete ITS region, significantly negative Fu's *F*_s_ and Tajima's *D* estimates were identified in Lineage C. In contrast, in all investigated fungal datasets, Lineage A showed positive but not significant values of these statistics. Due to the low amount of samples, the Lineage B could not be examined in this regard (Table 1, Fu's *F*s and Tajima's *D* estimates).

**Table 1:** Genetic diversity in the three mycobiont lineages (A, B, and C) of Thamnolia. The table includes information about the nuclear markers used, the total number of sequences without clone correction (m) and with clone correction (m*), genotypic diversity calculated as Gdiv = m*/m, the mean nucleotide diversity with the 95% confidence intervals (CI). The measurements were performed without clone correction (π) and with clone correction (π*). Significant Fu's *F*_s_ and Tajima's *D* values of the non‐clone‐corrected datasets are shown in boldface

Genetic information	Fungal dataset	Lineage	m	m*	Gdiv	mean π	π (95% CI)	mean π *	π* (95% CI)	Fu's *F*_s_	Tajima's *D*
ITS	F1	A	9	5	0.556	0.0045	0.0034–0.0054	0.0046	0.0024–0.0060	0.316	0.686
		B	10	2	0.200	0.0007	0.0005–0.0008	n/a	n/a	n/a	n/a
		C	135	25	0.185	0.0017	0.0013–0.0025	0.0037	0.0027–0.0047	−**18.992**	−2.0819*
ITS, EFα, DEAD	F3	A	9	5	0.556	0.0024	0.0016–0.0029	0.0025	0.0015–0.0033	0.458	0.416
		B	5	2	0.400	0.0002	0.0000–0.0004	n/a	n/a	n/a	n/a
		C	115	33	0.287	0.0023	0.0021–0.0026	0.0029	0.0025–0.0034	−20.944	−1.046
ITS, IGS, EFα, DEAD, RPB2, β‐tub	F4	A	8	5	0.625	0.0015	0.0009–0.0018	0.0015	0.0008–0.0021	0.410	0.4130
		B	3	2	0.667	0.0003	0.0000–0.0005	n/a	n/a	n/a	n/a
		C	39	14	0.359	0.0024	0.0019–0.0027	0.0023	0.0015–0.0030	−0.691	0.138

ITS, internal transcribed spacer; IGS, intergenic transcribed spacer; DEAD, dead‐box helicase; EFα, elongation factor alpha; β‐tub, beta tubulin; RPB2, RNA polymerase II second largest; *estimate after clone correction; n/a, not applicable.

### 3.7 Both Lineages A and C show genetic signatures of recombination

According to the 4GT analyses, signs of recombination were not detected in Lineage A, but we found that the *I*_A_ was not significantly different from zero, and therefore, the null hypothesis of recombination could not be rejected (see Dataset F4 in Table S8, Figure S6a). In the case of Lineage B, we could not find all possible four gametes combinations, and we had too little data to carry out an I_A_ analysis (Table S8). All the methods applied for detecting recombination were positive in Lineage C. Four gametes combinations and *R*_m_ sites (Tables S8 and S9e–g) were identified in all fungal dataset (except Dataset F1 that is based on only half of the ITS region). Additionally, in Dataset F3 that uses three molecular markers and samples from across the whole Northern Hemisphere, the index of association for Lineage C was not significantly different from zero and the null hypothesis of recombination could not be rejected (Table S8, Figure S7a). However, the high clonality of Lineage C is confirmed with Dataset F4 that has more genetic information but the fewest samples and shows LD: *I*_A_ values are significantly different from zero (*I*_A_ = 0.905; *p* = .002) (Table S8, Figure S6b). When all three lineages were considered as one population, 4GT identified several sites with the combination of four gametes and *R*_m_ (Tables S8 and S9a–d). However, *I*_A_ had significantly different from zero *p*‐values in all tested fungal datasets (Table S8, Figures S6c and S7b).

### 3.8 Genetic distances and relative divergence times between the three lineages

Pairwise divergences between the three lineages were measured using the dataset of the complete ITS region of 154 *Thamnolia* samples (Dataset F2; Table S5) as the average number of nucleotide substitutions per site (*D*_xy_). The divergence was low between Lineages A and B (*D*_xy_ = 0.0152), but higher and of the same magnitude between Lineages A and C (*D*_xy_ = 0.0223) and Lineages B and C (*D*_xy_ = 0.0232; Table 2). The relative divergence time estimates calculated based on three different ITS mutation rates, and net genetic distance between lineages, showed overlapping confidence intervals of the pairwise splits of the lineages. The split between Lineages A and B was estimated to occur within a confidence interval of 0.7–2.4 Myr bp, the split of Lineage A from C between 1.6 and 5.7 Myr bp, and between Lineages B and C between 1.8 and 6.6 Myr bp (Table 2).

**Table 2:** Pairwise genetic divergence and divergence time estimates of the three mycobiont lineages (A, B, and C). The average genetic distances (*d*_xy_), the net genetic distance in the confidence intervals of two standard errors (*d*_A_), and divergence times estimates based on mutation rates of *Oropogon*,* Melanelixia,* and *Erysiphe*

Pair	*d*_xy_	*d*_A_	Divergence times (Myr)		
			*Oropogon* μ	*Melanelixia* μ	*Erysiphales* μ
A‐B	0.0152	0.0031–0.0199	0.7–2.4	0.6–4.1	0.6–3.9
A‐C	0.0223	0.0098–0.0074	1.6–5.7	1.5–5.6	1.5–5.4
B‐C	0.0232	0.0112–0.0091	1.9–6.6	1.9–6.5	1.8–6.3

### 3.9 Thamnolia associates with photobionts belonging to four distinct Trebouxia lineages

**Figure 5:**
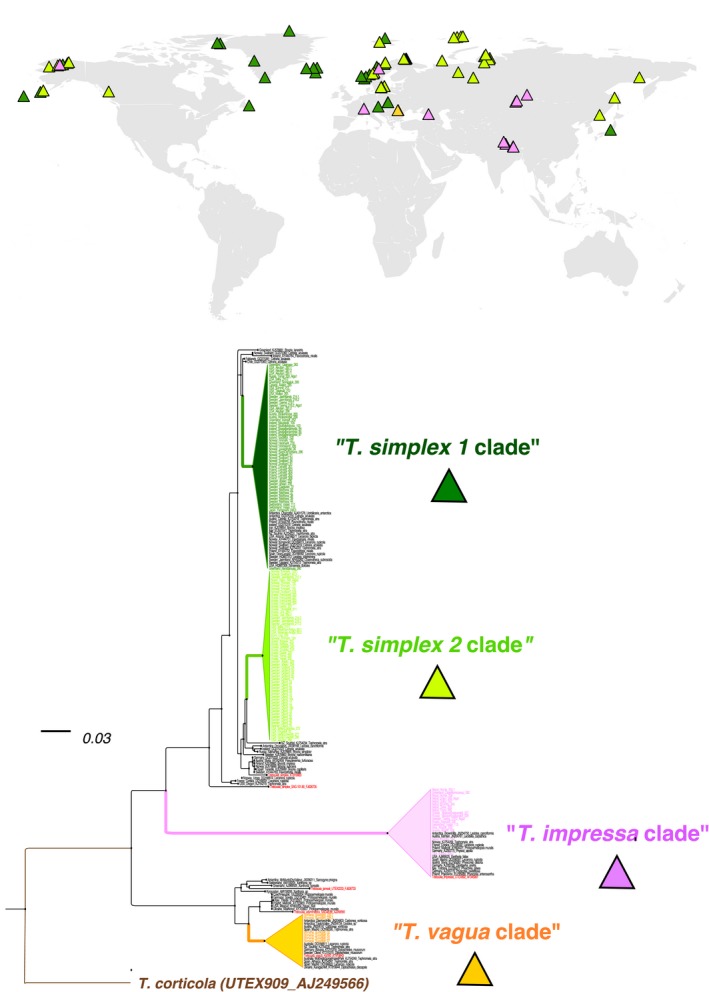
Rooted ML phylogeny of 214 ITS sequences of photobionts from *Thamnolia* and other lichens. The clades that contain photobionts from *Thamnolia* are collapsed and highlighted with colors, while the GenBank photobiont sequences from other lichens are shown in black. The geographic distribution of the *Trebouxia* photobionts is shown on the map as colored Δ according to the clade of origin. The tree was rooted with *Trebouxia corticola*. The scale bar indicates the branch length

The phylogenetic analyses of a dataset of 214 photobiont ITS sequences covering the geographic distribution range of *Thamnolia* (Dataset A1; Table S5) identified the following well‐supported photobiont clades: “*Trebouxia simplex* clade 1,” “*T. simplex* clade 2*,” “T. impressa* clade,*”* and “*T. vagua* clade” (Figure 5, Figures S8–S10). “*Trebouxia simplex* clade 1” was detected in thalli of *Thamnolia* from Europe, North America, and East Asia. It was found associated with all three mycobiont lineages (A, B, and C), and in thalli of both chemotypes (Figure S11). “*Trebouxia simplex* clade 2” in *Thamnolia* has a partially overlapping geographic distribution with “*T. simplex* clade 1” in Europe and Alaska, but was not found in the *Thamnolia* samples from Greenland (Figure 5). It was found associated with mycobiont Lineages A and C (both chemotypes), but not with B (Figure S11). “*Trebouxia impressa* clade” was dominating in *Thamnolia* samples from Central Asia, but was also encountered in a few localities in Europe and one in Alaska (Figure 5). However, *T. impressa* was found in association with mycobionts of Lineage C only (Figure S11). “*Trebouxia vagua* clade” was only found in *Thamnolia* samples from Romania, and only associated with mycobionts of UV+ chemistry from Lineage C (Figure S11).

Except for “*Trebouxia simplex* clade 2,” all the other three *Trebouxia* lineages were also found in a variety of other lichen species across a wide geographic range (Figure 5, Figure S8–S10).

When extending the genetic information of 73 samples with two additional loci (actin and COX) (Dataset A2; Table S5), we obtained an increased support for three of the *Trebouxia* lineages (“*T*. *simplex* clade 1,” “*T. simplex* clade 2,” and “*T. vagua* clade”) associated with *Thamnolia*. The photobionts from “*Trebouxia simplex”* and “*T. vagua”* clades showed no genetic variation in the three investigated loci, as shown by the ML phylogeny (Figure S12).

## 4 Discussion

Lichens are formed as a symbiosis between two main partners, a mycobiont and a photobiont. Recent lichen studies strive to integrate both morphological and molecular information from the symbiotic partners (e.g., Chen, Werth, & Sork, 2016; Fernandez‐Mendoza et al., 2011; Perez‐Ortega, Ortiz‐Alvarez, Allan Green, & de Los Rios, 2012; Spribille et al., 2016; Werth & Sork, 2010). In this study, we focused our efforts to describe and understand the mycobiont population structure and the associated photobionts in *Thamnolia*.

### 4.1 Three mycobiont lineages in Thamnolia

By analyzing molecular, morphological, and chemical variation among 253 specimens well covering the distribution range of the species, we revealed the existence of three mycobiont lineages—Lineages A, B, and C. Even if the rooted ML phylogeny did not resolve the phylogenetic relationships among the three lineages (Figure 3), the clear separation of them in the haplotype networks showed that they are distinct from each other and indicate that there is limited, or no, gene flow between them. Our finding is consistent with numerous reports of morphologically defined species of both free‐living and symbiotic fungi that include closely related “cryptic” species (e.g., Crespo & Lumbsch, 2010; Lumbsch & Leavitt, 2011; Taylor et al., 2000). Our data revealed partly overlapping geographic distributions of the three lineages (Figures 3 and 4). Lineage C is the most widely distributed and is in part sympatric with both A and B. The latter two lineages, however, are allopatric and found in two separate ecosystems. The comparatively narrow geographic ranges of Lineages A and B suggest an adaptation to arctic and alpine tundra, respectively, and/or that they have limited dispersal, possibly spreading only as thallus fragments over short distances. Yet another factor that may have influenced the present distributions of the lineages is their demographic history related to the Pleistocene (see below).

### 4.2 The reproductive mode of the three mycobiont lineages

Even if the lack of apothecia in *Thamnolia* suggests that this species is sterile, two different approaches to detect recombination revealed signs of recombination in both Lineages A and C. Based on the overall genetic structure, we propose that the reproductive strategies are mainly clonal with signatures of past or infrequent recombination events. Specifically, the low genetic variability in all six nuclear markers (described in Table S5), the widespread distribution of clones (i.e., identical genotypes for the markers investigated here), and the star‐like reticulation of the haplotypes are strong indications of a predominantly asexual mode of reproduction (Figures S3 and S4). Even when using the genetic information from six nuclear markers (Dataset F4; Table S5), we found that some regions, such as Iceland, Romania, Svalbard, Sweden (Öland) and Norway (Mo i Rana) were highly or even completely dominated by one mycobiont genotype (Figure 4). Identical genotypes were, however, also encountered in areas very far apart. Illustrative examples are haplotype H1c that was found in Australia and the Americas, and H1i, found in both North and South America and also in Central Asia (Figure S3). When comparing the proportion of clones (Gdiv) and nucleotide diversity (π) between the three mycobiont lineages, we found that Lineage B has a much lower genetic diversity compared with the similar ones of Lineages A and C (Table 1). Lineage A shows no reticulation of the network and the genetic distance between the five haplotypes of this lineage does increase with the amount of genetic information used (see Figure S3 in comparison with Figure S4). The finding of a predominant clonal propagation with a low level of recombination in *Thamnolia* is consistent with the emerging view that exclusive clonal reproduction in fungi does not exist (Taylor, Branco, Sylvain, & Ellison, 2015). The mechanisms for the detected recombination are still not understood, but may involve both meiotic and nonmeiotic processes (e.g., Ene & Bennett, 2014) and possibly a nonlichenized part of the life cycle (Tibell, 1997; Wedin, Döring, & Gilenstam, 2004).

### 4.3 *Demographic history of the* Thamnolia *lineages*

The divergence time estimates suggest that the split of the three lineages occurred before the onset of the last glacial period, and thus, the divergence itself is not related to ice age events of this period (Table 2). Nevertheless, according to Shafer, Cullingham, Cote, and Coltman (2010), organisms with limited dispersal abilities and habitat specialization frequently have a population structure that mirrors their Pleistocene distribution. We argue that the Pleistocene glaciation might have dramatically influenced the current distribution of each of the three lineages independently. The present‐day distributions of Lineages A and B are restricted to limited areas and specific habitats of the Northern Hemisphere (Figure 3 and Table S7), and this supports the hypothesis that their distribution during the last glaciation might have been restricted to refugia, one in the northern Eurasia (Lineage A) (*c.f*. Parducci et al., 2012; Tarasov et al., 2000) and one in southern Europe (Lineage B) (c.f. Schönswetter, Stehlik, Holderegger, & Tribsch, 2005; Widmer et al., 2012). The TCS haplotype networks suggest that the current distribution of Lineage C has its origin in the Northern Hemisphere (Figure S3), possibly in Scandinavia (Figure S4). The significantly negative values of Tajima's *D* and Fu's *F*_s_ suggest that Lineage C went through a bottleneck with recent population expansion (Table 1). Noteworthy, the lichen *Cetraria aculeata* that often co‐occurs with *T. vermicularis* has also been suggested to originate from the Northern Hemisphere and after a population size expansion disperses southward (Fernandez‐Mendoza & Printzen, 2013). We were with this dataset unable to detect any migration routes of the *Thamnolia* lineages (data not shown), and future studies including more samples and/or more genetic information are needed to fully understand their demographic histories.

### 4.4 *Thamnolia vermicularis* associates with different photobionts

Our understanding of the relationships between the symbiotic partners and their role within the lichen symbiosis is still fragmentary. A series of studies, however, strongly suggest that lichens, even if not closely related, often share photobionts (Rikkinen, Oksanen, & Lohtander, 2002). In this study, we showed that while Lineage A and Lineage B showed a high photobiont specificity, Lineage C was associated with four different photobiont lineages (Figure 5). These differences between the lineages could be attributed to generalist/specialist ecology of the different mycobiont lineages, geographic distribution, and/or samples size. There is little consensus on species recognition and naming in *Trebouxia*, but we were able to assign the *Thamnolia* photobionts to two closely related lineages from *T. simplex (“simplex 1”* and *“simplex 2”),* one lineage of the “*T. impressa”* clade and one of the “*T. vagua”* clade. This finding of lack of photobiont specificity is consistent with previous studies of photobiont switching in *Thamnolia* (Nelsen & Gargas, 2009b). Our sampling over a wide geographic range revealed a possible geographic pattern among the photobiont lineages. Figure 5 shows that large geographic areas are entirely or partially dominated by one of the algal lineages (e.g., Greenland where only *T. simplex 1* is present, or Central Asia where *T. impressa* is found in high frequency). This pattern is consistent with dispersal by symbiotic fragments, but may also be explained by the existence of locally adapted photobionts shared with other lichens, as *Thamnolia* photobionts from the clades of “*T. simplex 1*,” “*T. impressa,”* and “*T. vagua”* have also been found in other lichens (Figures S8–S10).

Our study showed that *Thamnolia* occasionally contains more than one photobiont within a single podetium. Specifically, in two cases, different photobionts were amplified from the same podetium: once in a sample from Nepal (with both *T. impressa* and “*T. simplex 2”*) and also in another sample from Sweden, contained both *T. simplex* lineages (Table S7). Carrying multiple photobionts within a lichen thallus might be an adaptive trait in *Thamnolia*, as was suggested for *Ramalina farinacea*, which always contains two different *Trebouxia* lineages with different physiological performances (Casano et al., 2011).

### 4.5 Chemistry and morphology of Thamnolia: biologic and taxonomic implications

Confidence in secondary chemistry as a reliable characteristic for species recognition has diminished with the increased number of phylogenetic studies showing that chemotypes of a species do not form monophyletic groups. This pattern was also shown in a previous study of *T. vermicularis* in which a smaller sampling (26 podetia) revealed that the two chemotypes do not form monophyletic groups (Nelsen & Gargas, 2009a). In our more comprehensive sampling, this finding was confirmed (Figures 2, 3, 4). Thus, the two chemotypes of *T. vermicularis* are not to be regarded as separate species. Both chemotypes of Lineage C occur in the same patch (i.e.*,* within a few centimeters), and hence, the chemotype does not seem to be a response to environmental conditions. Furthermore, individuals of different chemotypes sometimes contain the same algal genotype and the chemotype is thus also not determined by photobiont genotype (Table S7). Other possible explanations for differences in chemistry might be gene expression of either symbiont (Nelsen & Gargas, 2009a), or influence from other cohabitants (Spribille et al., 2016). Thallus morphology also provides little help in identifying genetically distinct mycobiont lineages. Our study shows that the morphologically circumscribed *T. papelillo* (with flat podetia) does not form a clade but groups with specimens with cylindrical podetia (Table S1). Hence, neither podetium shape is a good proxy to circumscribe species (Figures 2 and 3). In conclusion, we suggest that the chemotypes in *T. vermicularis* Lineage C should be seen as chemical variation within a species and *Thamnolia papelillo* included in this lineage in spite of its deviant podetium morphology.

## Conflict of Interest

None declared.

## Author Contributions

Ioana Onut‐Brännström contributed to the paper through study design, material collection, laboratory work, data analyses, and writing of the manuscript. Leif Tibell contributed to the paper through study design, material collection, and writing of the manuscript. Hanna Johannesson contributed to the paper through study design and writing of the manuscript.

## Data Accessibility

All the herbarium vouchers are deposited in UPS (The Museum of Evolution Herbarium—Uppsala, Sweden), and the herbarium accessions numbers are indicated in Table S2.

All the DNA sequences have been deposited in GenBank and included in Table S6.

The final DNA sequences alignments and the scaffolds containing the genes of interest are available from the Dryad Digital Repository: https://doi.org/10.5061/dryad.79d91.

Sampling locations (including GPS coordinates) and morphological data can be found in Table S2 and the shared map from Google maps: (https://drive.google.com/open?id=1JHKT8Hcaozr3Dxr2BPviOdZWNP8&usp=sharing).
