# Supplementary material for: A worldwide phylogeography of the whiteworm lichens Thamnolia reveals three lineages with distinct habitats and evolutionary histories
Source: Ecol Evol. 2017 Apr 13;7(10):3602–15. doi: 10.1002/ece3.2917 (PMC5433967; doi:10.1002/ece3.2917)
Supplement: Supplementary file 1 [file ECE3-7-3602-s001.pdf]

A. Supporting Information (Figures)

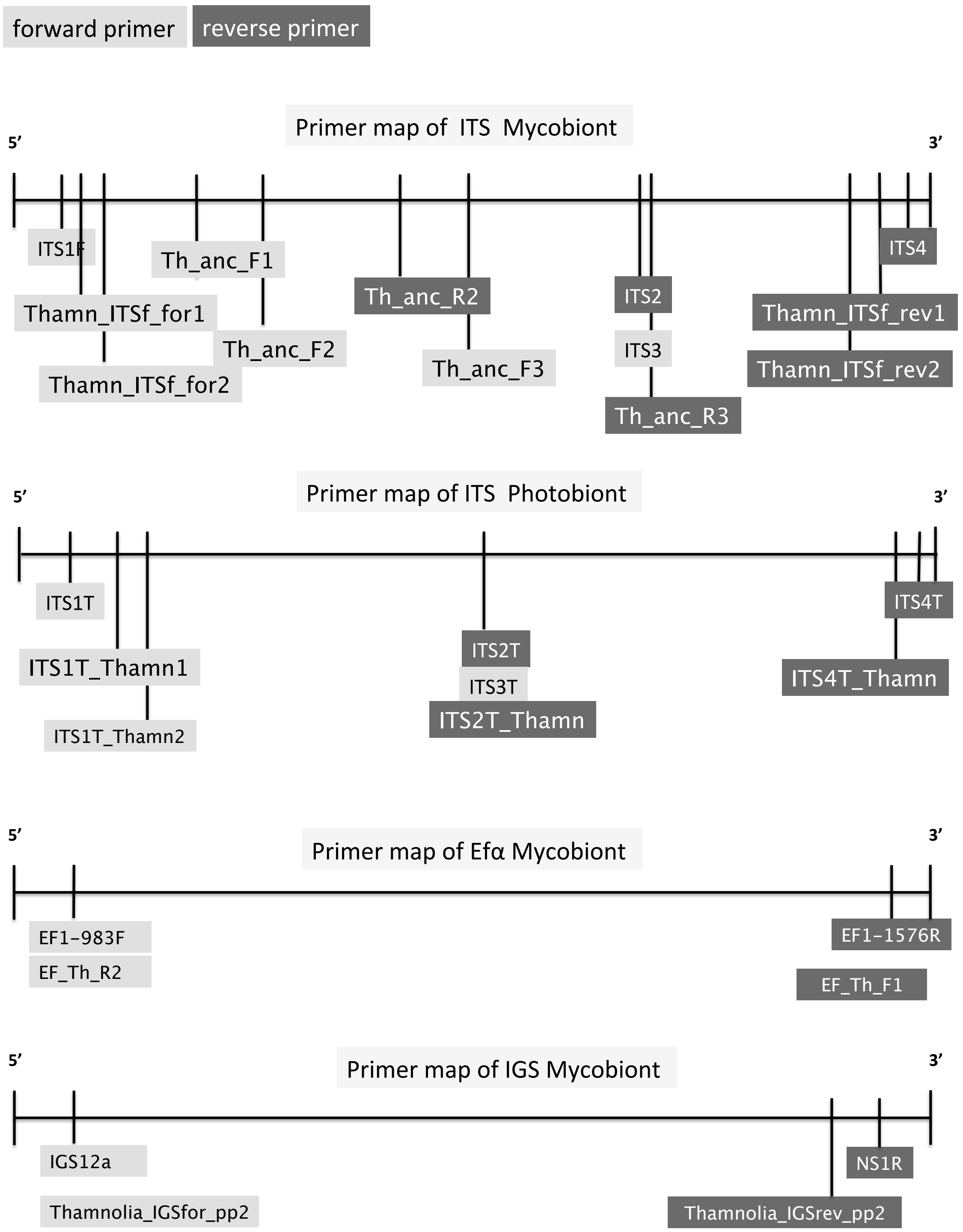

Fig S1 Primer maps of the markers for which more then one primer combination was used. Color highlight indicate primer direction: pink indicate forward direction and blue reverse direction. Marker used: EFα (translation elongation factor 1-alpha gene); ITS (internal transcribed spacer); IGS (intergenic spacer region).

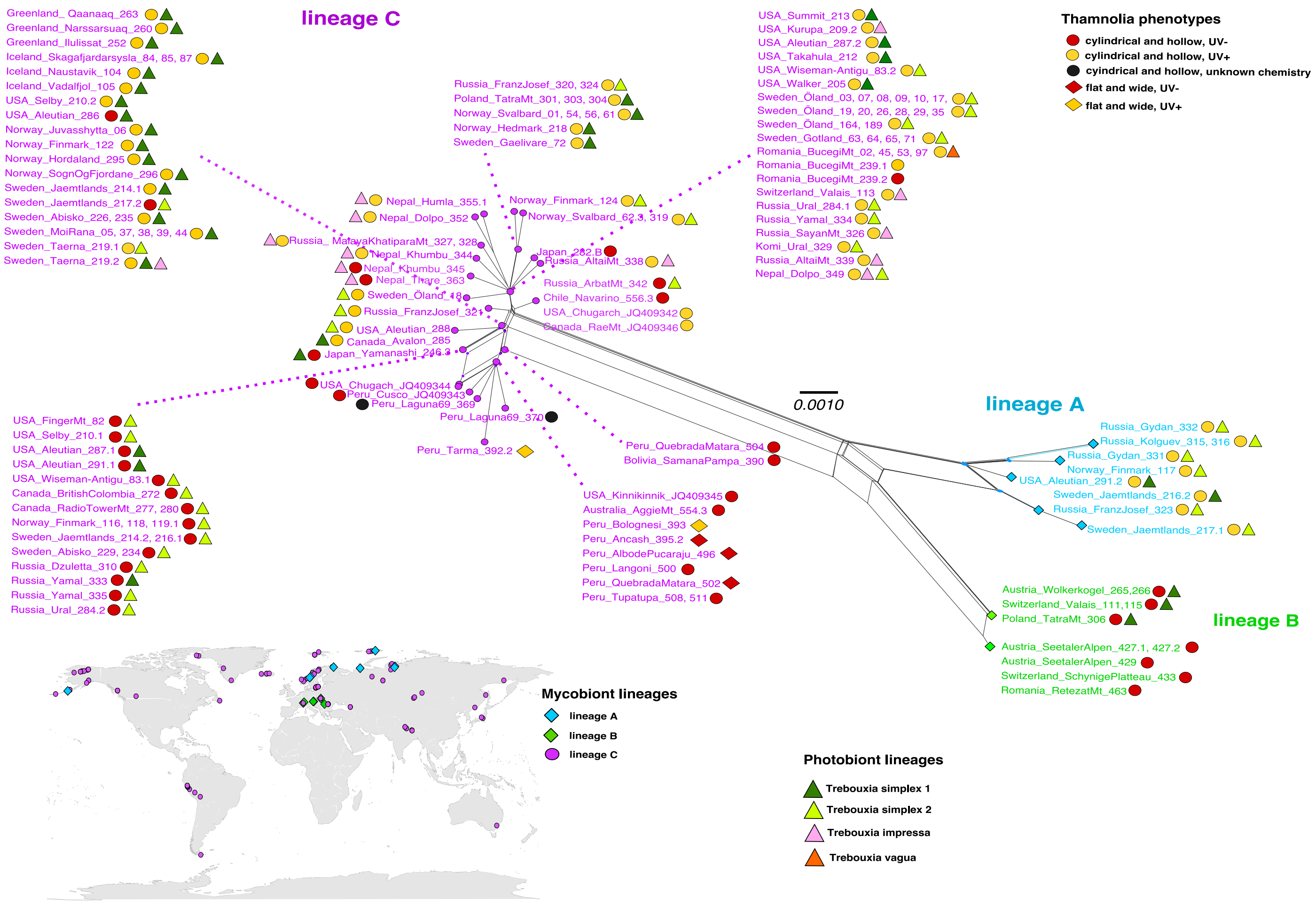

Fig S2 SplitsTree haplotype network of 154 *Thamnolia* samples, highlighting the the mycobiont lineages A (blue), B (green) and C (purple) of *Thamnolia*. Thallus features (chemistry and morphology) are indicated for each sample as colored **O** and ◆. The photobiont lineage is indicated by Δ. The scale bar indicates the branch lengths.

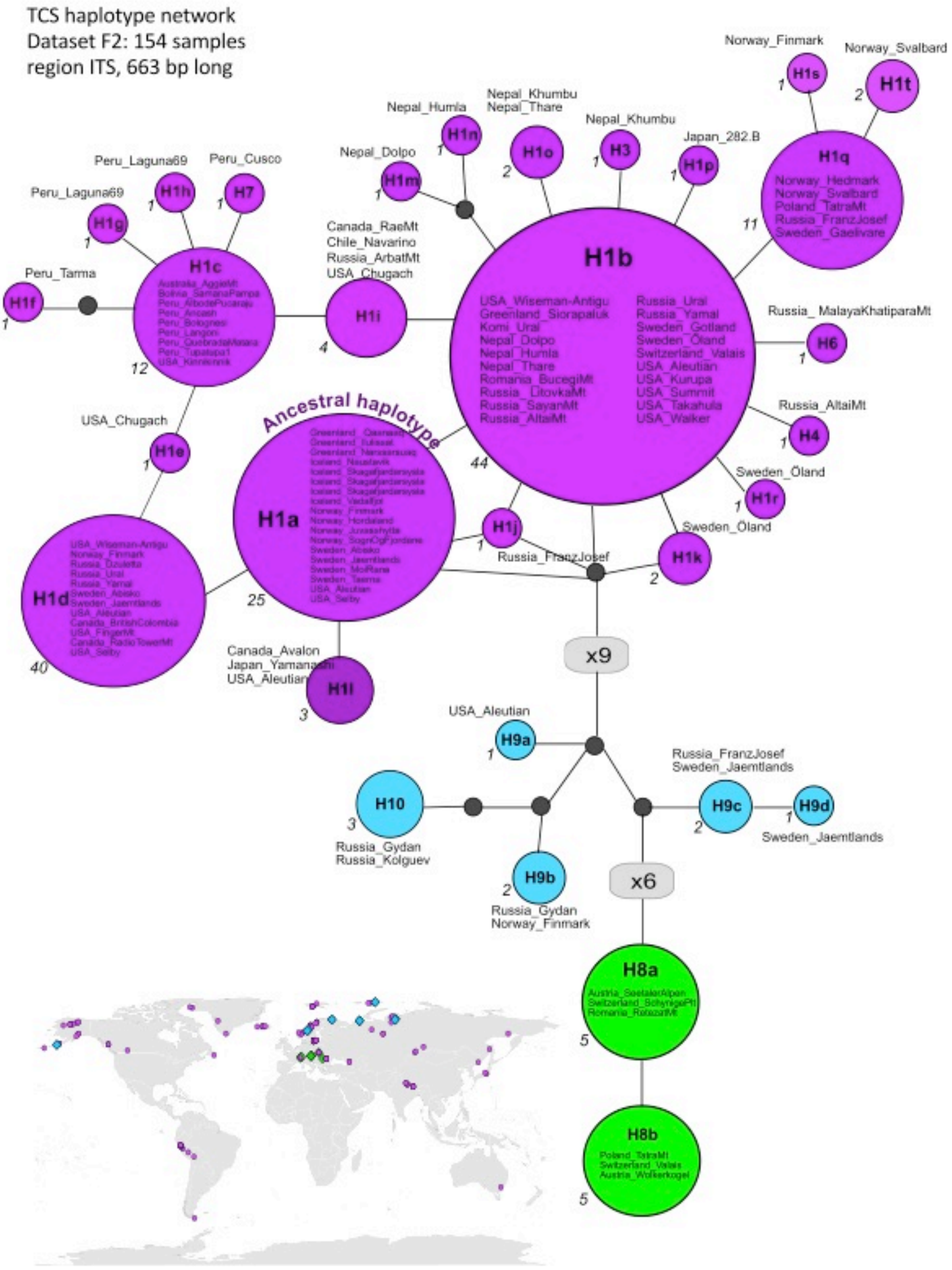

Fig S3 TCS haplotype network of 154 samples of *Thamnolia* with indication of lineage (lineages A (blue), B (green) and C (purple)) and geographic distribution. The size of each circle is proportional to the number of samples belonging to a haplogroup and the total number of samples is shown by each circle. The figure shows the haplogroup ID, the sample origin, and the designation of the ancestral haplotype (H1a).

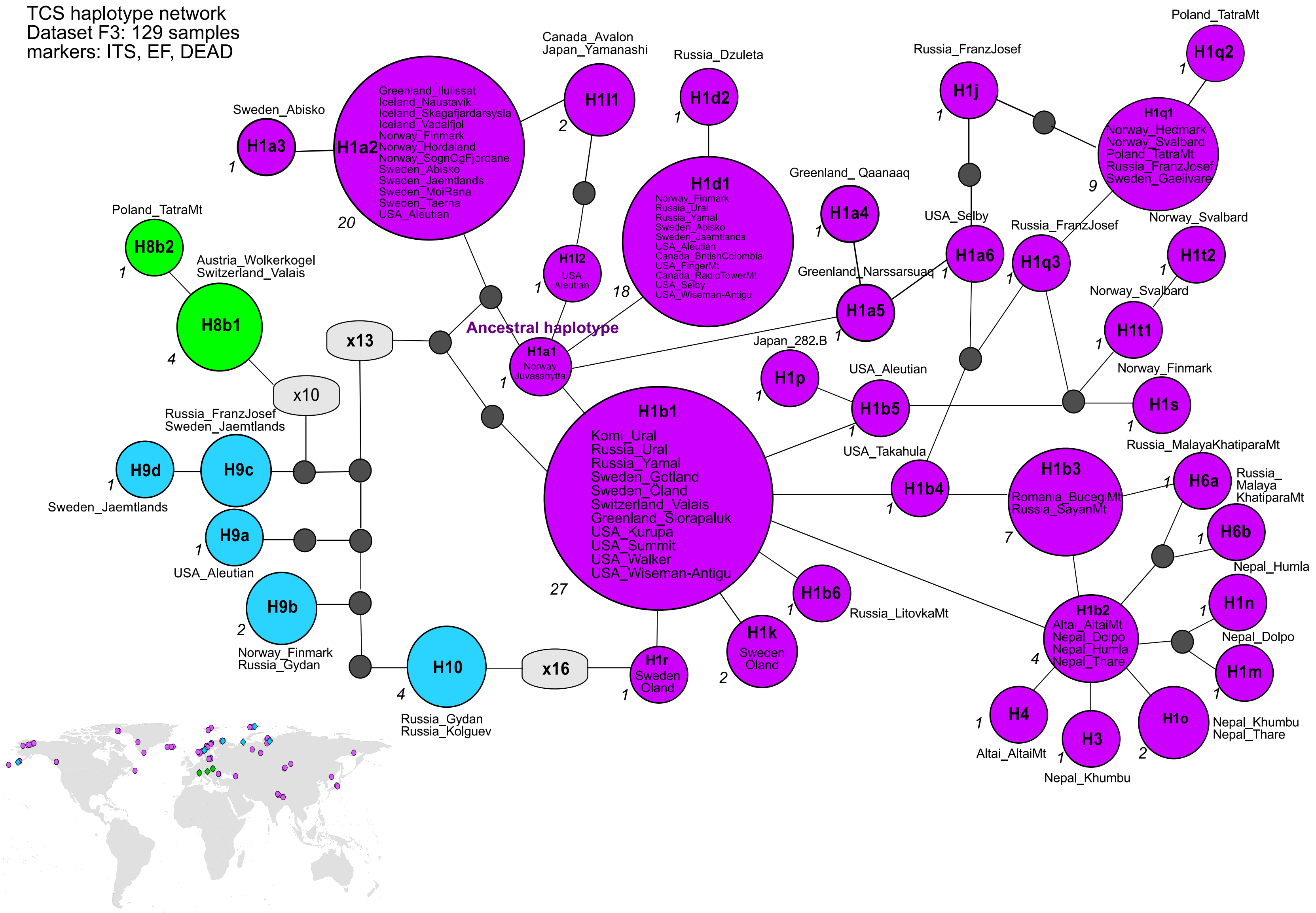

Fig S4 TCS haplotype network of 129 samples of *Thamnolia*, highlighting in different colors the haplogroups of the three lineages: A (blue), B (green) and C (purple). The size of each circle is proportional to the number of samples, and the total number of samples is shown by each circle. The figure shows the haplogroup ID, the sample origin and the designation of the ancestral haplotype (H1a1). A map shows the distribution of the samples.

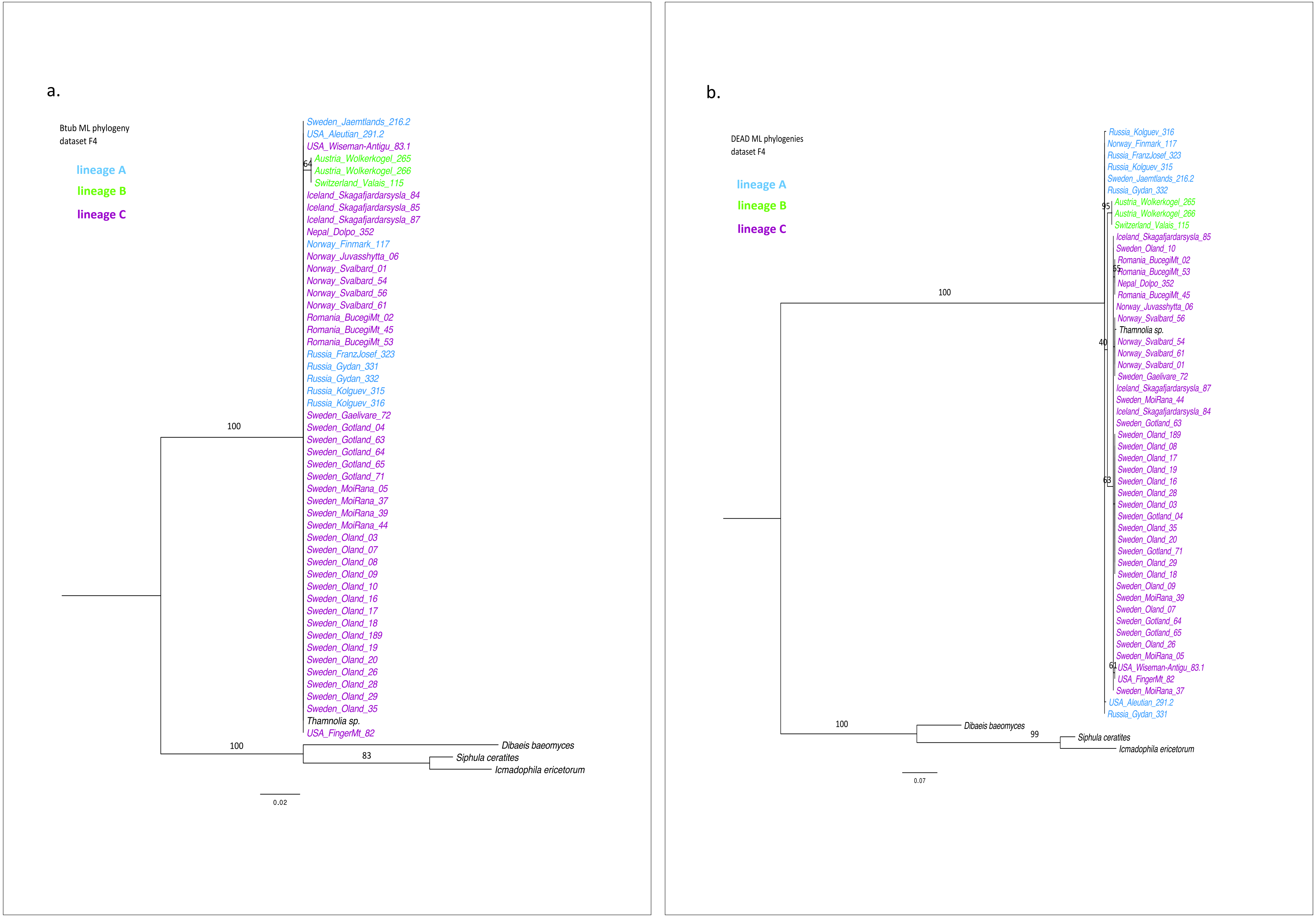
a.
Btub ML phylogeny
dataset F4
lineage A
lineage B
lineage C
Sweden_Jaemtlands_216.2
USA_Aleutian_291.2
USA_Wiseman-Antigu_83.1
64
Austria_Wolkerkogel_265
Austria_Wolkerkogel_266
Switzerland_Valais_115
Iceland_Skagafjardarsysla_84
Iceland_Skagafjardarsysla_85
Iceland_Skagafjardarsysla_87
Nepal_Dolpo_352
Norway_Finmark_117
Norway_Juvasshytta_06
Norway_Svalbard_01
Norway_Svalbard_54
Norway_Svalbard_56
Norway_Svalbard_61
Romania_BucegiMt_02
Romania_BucegiMt_45
Romania_BucegiMt_53
Russia_FranzJosef_323
Russia_Gydan_331
Russia_Gydan_332
Russia_Kolguev_315
Russia_Kolguev_316
Sweden_Gaelivare_72
Sweden_Gotland_04
Sweden_Gotland_63
Sweden_Gotland_64
Sweden_Gotland_65
Sweden_Gotland_71
Sweden_MoiRana_05
Sweden_MoiRana_37
Sweden_MoiRana_39
Sweden_MoiRana_44
Sweden_Oland_03
Sweden_Oland_07
Sweden_Oland_08
Sweden_Oland_09
Sweden_Oland_10
Sweden_Oland_16
Sweden_Oland_17
Sweden_Oland_18
Sweden_Oland_189
Sweden_Oland_19
Sweden_Oland_20
Sweden_Oland_26
Sweden_Oland_28
Sweden_Oland_29
Sweden_Oland_35
Thamnolia sp.
USA_FingerMt_82
100
100
Dibaeis baeomyces
83
Siphula ceratites
Icmadophila ericetorum
0.02
b.
DEAD ML phylogenies
dataset F4
lineage A
lineage B
lineage C
Russia_Kolguev_316
Norway_Finmark_117
Russia_FranzJosef_323
Russia_Kolguev_315
Sweden_Jaemtlands_216.2
Russia_Gydan_332
95
Austria_Wolkerkogel_265
Austria_Wolkerkogel_266
Switzerland_Valais_115
Iceland_Skagafjardarsysla_85
Sweden_Oland_10
Romania_BucegiMt_02
55
Romania_BucegiMt_53
Nepal_Dolpo_352
Romania_BucegiMt_45
Norway_Juvasshytta_06
Norway_Svalbard_56
Thamnolia sp.
40
Norway_Svalbard_54
Norway_Svalbard_61
Norway_Svalbard_01
Sweden_Gaelivare_72
Iceland_Skagafjardarsysla_87
Sweden_MoiRana_44
Iceland_Skagafjardarsysla_84
Sweden_Gotland_63
Sweden_Oland_189
Sweden_Oland_08
Sweden_Oland_17
Sweden_Oland_19
63
Sweden_Oland_16
Sweden_Oland_28
Sweden_Oland_03
Sweden_Gotland_04
Sweden_Oland_35
Sweden_Oland_20
Sweden_Gotland_71
Sweden_Oland_29
Sweden_Oland_18
Sweden_Oland_09
Sweden_MoiRana_39
Sweden_Oland_07
Sweden_Gotland_64
Sweden_Gotland_65
Sweden_Oland_26
Sweden_MoiRana_05
61
USA_Wiseman-Antigu_83.1
USA_FingerMt_82
Sweden_MoiRana_37
USA_Aleutian_291.2
Russia_Gydan_331
100
100
Dibaeis baeomyces
99
Siphula ceratites
Icmadophila ericetorum
0.07

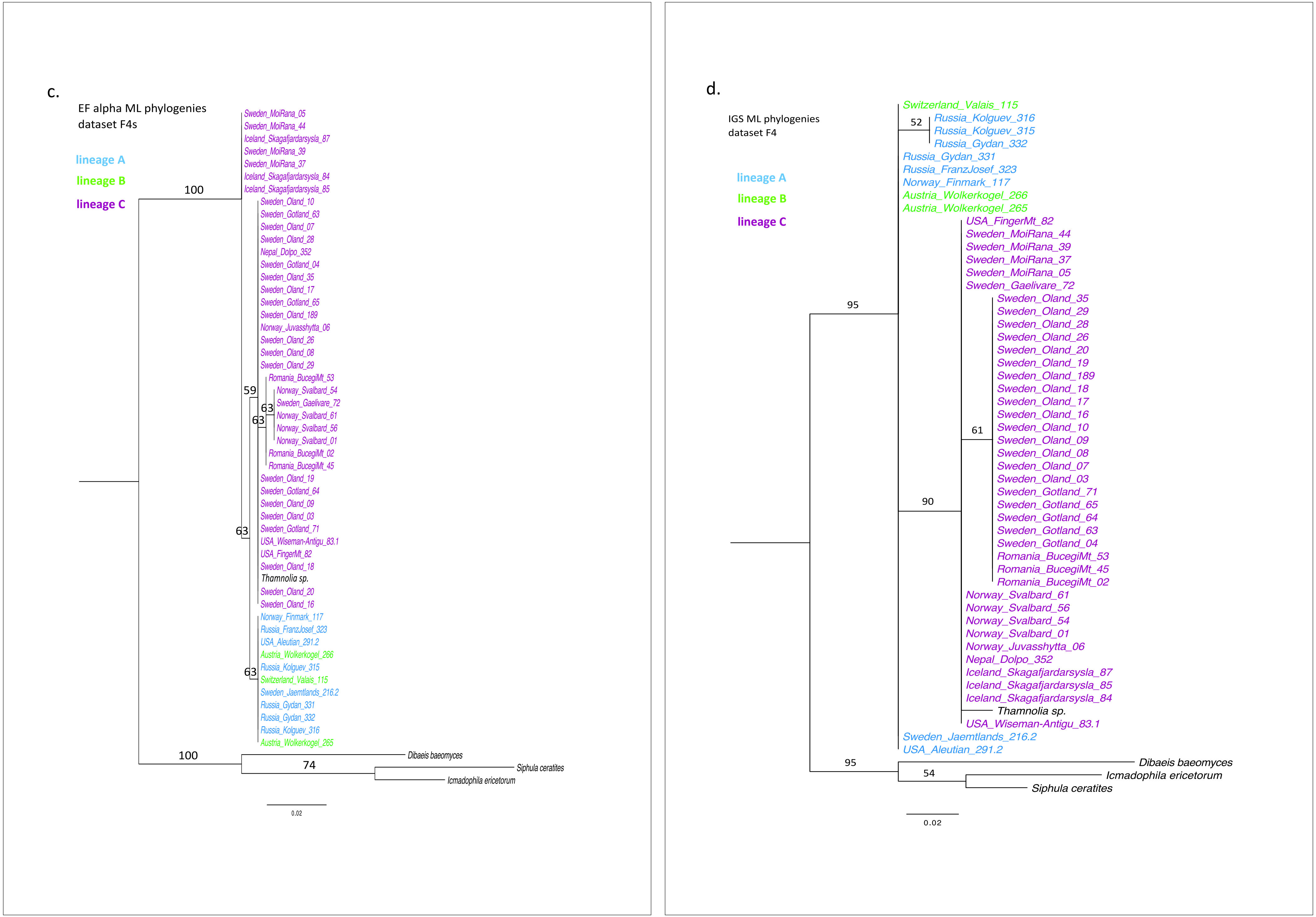

c.
EF alpha ML phylogenies
dataset F4s
lineage A
lineage B
lineage C
Sweden_MoiRana_05
Sweden_MoiRana_44
Iceland_Skagafjardarsysla_87
Sweden_MoiRana_39
Sweden_MoiRana_37
Iceland_Skagafjardarsysla_84
Iceland_Skagafjardarsysla_85
Sweden_Oland_10
Sweden_Gotland_63
Sweden_Oland_07
Sweden_Oland_28
Nepal_Dolpo_352
Sweden_Gotland_04
Sweden_Oland_35
Sweden_Oland_17
Sweden_Gotland_65
Sweden_Oland_189
Norway_Juvasshytta_06
Sweden_Oland_26
Sweden_Oland_08
Sweden_Oland_29
Romania_BucegiMt_53
Norway_Svalbard_54
Sweden_Gaelivare_72
Norway_Svalbard_61
Norway_Svalbard_56
Norway_Svalbard_01
Romania_BucegiMt_02
Romania_BucegiMt_45
Sweden_Oland_19
Sweden_Gotland_64
Sweden_Oland_09
Sweden_Oland_03
Sweden_Gotland_71
USA_Wiseman-Antigu_83.1
USA_FingerMt_82
Sweden_Oland_18
Thamnolia sp.
Sweden_Oland_20
Sweden_Oland_16
Norway_Finmark_117
Russia_FranzJosef_323
USA_Aleutian_291.2
Austria_Wolkerkogel_266
Russia_Kolguev_315
Switzerland_Valais_115
Sweden_Jaemtlands_216.2
Russia_Gydan_331
Russia_Gydan_332
Russia_Kolguev_316
Austria_Wolkerkogel_265
Dibaeis baeomyces
Siphula ceratites
Icmadophila ericetorum
100
59
63
63
63
63
100
74
0.02
d.
IGS ML phylogenies
dataset F4
lineage A
lineage B
lineage C
Switzerland_Valais_115
Russia_Kolguev_316
Russia_Kolguev_315
Russia_Gydan_332
Russia_Gydan_331
Russia_FranzJosef_323
Norway_Finmark_117
Austria_Wolkerkogel_266
Austria_Wolkerkogel_265
USA_FingerMt_82
Sweden_MoiRana_44
Sweden_MoiRana_39
Sweden_MoiRana_37
Sweden_MoiRana_05
Sweden_Gaelivare_72
Sweden_Oland_35
Sweden_Oland_29
Sweden_Oland_28
Sweden_Oland_26
Sweden_Oland_20
Sweden_Oland_19
Sweden_Oland_189
Sweden_Oland_18
Sweden_Oland_17
Sweden_Oland_16
Sweden_Oland_10
Sweden_Oland_09
Sweden_Oland_08
Sweden_Oland_07
Sweden_Oland_03
Sweden_Gotland_71
Sweden_Gotland_65
Sweden_Gotland_64
Sweden_Gotland_63
Sweden_Gotland_04
Romania_BucegiMt_53
Romania_BucegiMt_45
Romania_BucegiMt_02
Norway_Svalbard_61
Norway_Svalbard_56
Norway_Svalbard_54
Norway_Svalbard_01
Norway_Juvasshytta_06
Nepal_Dolpo_352
Iceland_Skagafjardarsysla_87
Iceland_Skagafjardarsysla_85
Iceland_Skagafjardarsysla_84
Thamnolia sp.
USA_Wiseman-Antigu_83.1
Sweden_Jaemtlands_216.2
USA_Aleutian_291.2
Dibaeis baeomyces
Icmadophila ericetorum
Siphula ceratites
52
95
61
90
95
54
0.02

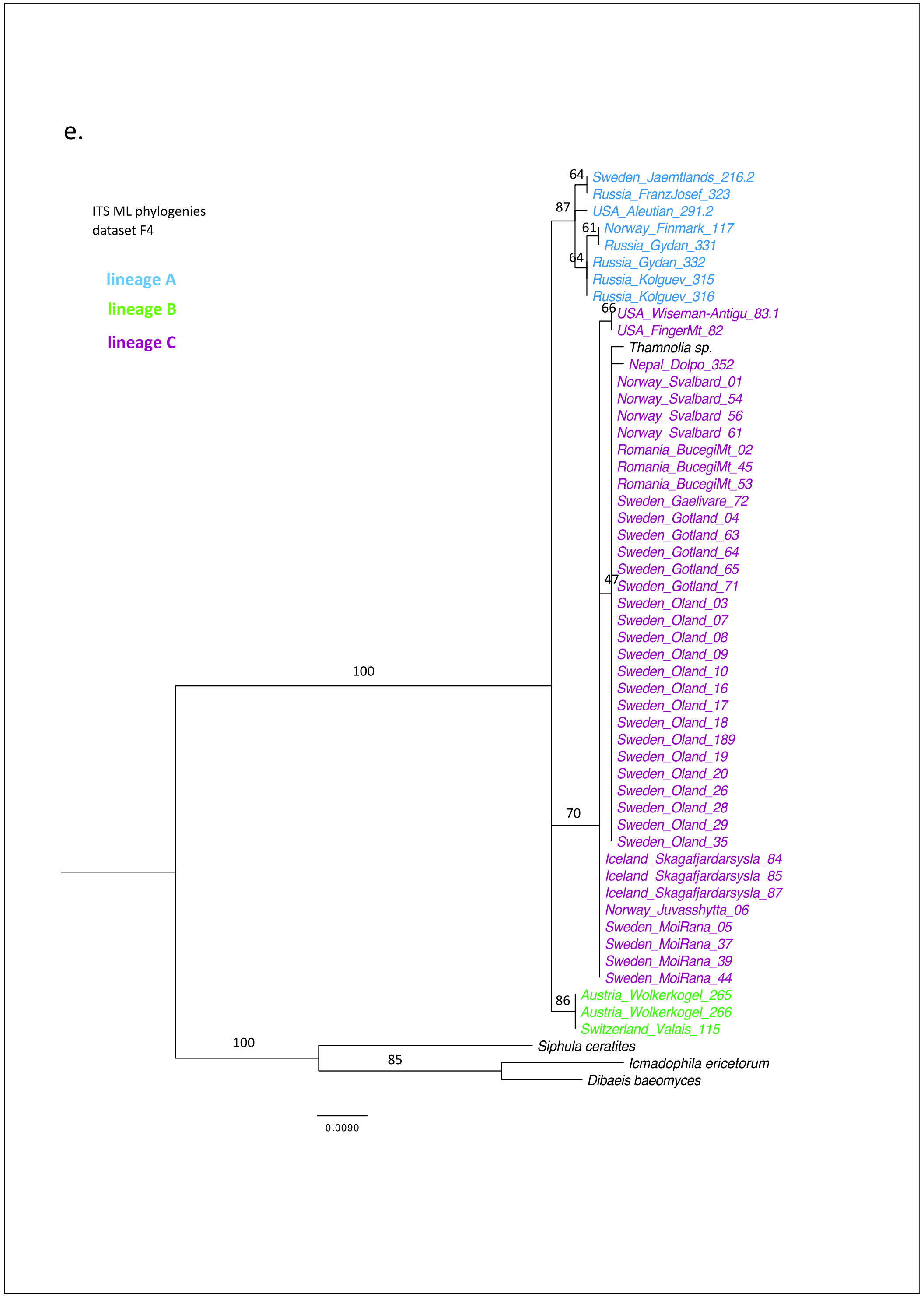
e.
ITS ML phylogenies
dataset F4
lineage A
lineage B
lineage C
Sweden_Jaemtlands_216.2
Russia_FranzJosef_323
USA_Aleutian_291.2
Norway_Finmark_117
Russia_Gydan_331
Russia_Gydan_332
Russia_Kolguev_315
Russia_Kolguev_316
USA_Wiseman-Antigu_83.1
USA_FingerMt_82
Thamnolia sp.
Nepal_Dolpo_352
Norway_Svalbard_01
Norway_Svalbard_54
Norway_Svalbard_56
Norway_Svalbard_61
Romania_BucegiMt_02
Romania_BucegiMt_45
Romania_BucegiMt_53
Sweden_Gaelivare_72
Sweden_Gotland_04
Sweden_Gotland_63
Sweden_Gotland_64
Sweden_Gotland_65
Sweden_Gotland_71
Sweden_Oland_03
Sweden_Oland_07
Sweden_Oland_08
Sweden_Oland_09
Sweden_Oland_10
Sweden_Oland_16
Sweden_Oland_17
Sweden_Oland_18
Sweden_Oland_189
Sweden_Oland_19
Sweden_Oland_20
Sweden_Oland_26
Sweden_Oland_28
Sweden_Oland_29
Sweden_Oland_35
Iceland_Skagafjardarsysla_84
Iceland_Skagafjardarsysla_85
Iceland_Skagafjardarsysla_87
Norway_Juvasshytta_06
Sweden_MoiRana_05
Sweden_MoiRana_37
Sweden_MoiRana_39
Sweden_MoiRana_44
Austria_Wolkerkogel_265
Austria_Wolkerkogel_266
Switzerland_Valais_115
Siphula ceratites
Icmadophila ericetorum
Dibaeis baeomyces
64
87
61
64
66
47
100
70
86
100
85
0.0090

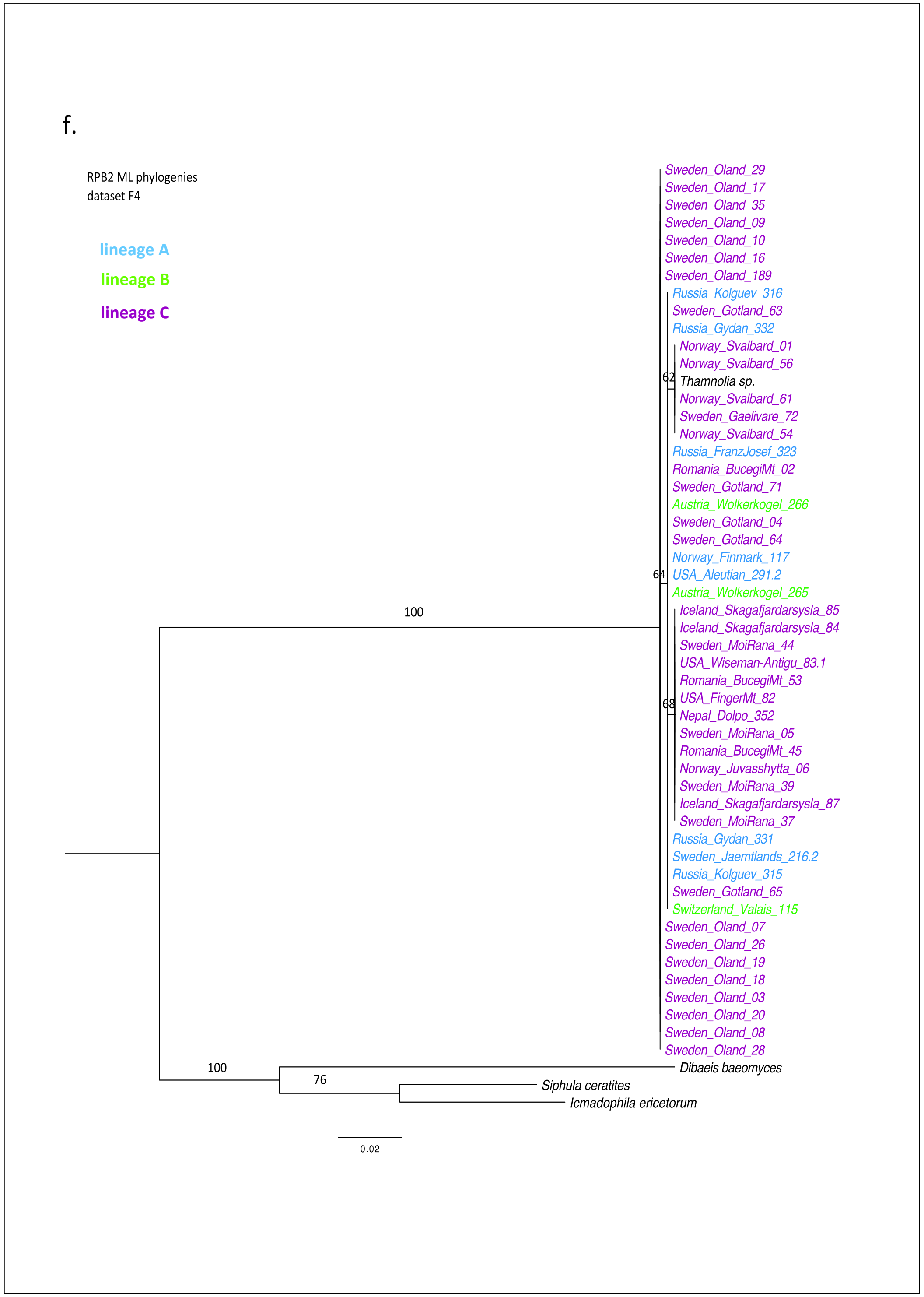
f.
RPB2 ML phylogenies
dataset F4
lineage A
lineage B
lineage C
Sweden_Oland_29
Sweden_Oland_17
Sweden_Oland_35
Sweden_Oland_09
Sweden_Oland_10
Sweden_Oland_16
Sweden_Oland_189
Russia_Kolguev_316
Sweden_Gotland_63
Russia_Gydan_332
Norway_Svalbard_01
Norway_Svalbard_56
Thamnolia sp.
Norway_Svalbard_61
Sweden_Gaelivare_72
Norway_Svalbard_54
Russia_FranzJosef_323
Romania_BucegiMt_02
Sweden_Gotland_71
Austria_Wolkerkogel_266
Sweden_Gotland_04
Sweden_Gotland_64
Norway_Finmark_117
USA_Aleutian_291.2
Austria_Wolkerkogel_265
Iceland_Skagafjardarsysla_85
Iceland_Skagafjardarsysla_84
Sweden_MoiRana_44
USA_Wiseman-Antigu_83.1
Romania_BucegiMt_53
USA_FingerMt_82
Nepal_Dolpo_352
Sweden_MoiRana_05
Romania_BucegiMt_45
Norway_Juvasshytta_06
Sweden_MoiRana_39
Iceland_Skagafjardarsysla_87
Sweden_MoiRana_37
Russia_Gydan_331
Sweden_Jaemtlands_216.2
Russia_Kolguev_315
Sweden_Gotland_65
Switzerland_Valais_115
Sweden_Oland_07
Sweden_Oland_26
Sweden_Oland_19
Sweden_Oland_18
Sweden_Oland_03
Sweden_Oland_20
Sweden_Oland_08
Sweden_Oland_28
Dibaeis baeomyces
Siphula ceratites
Icmadophila ericetorum
62
64
68
100
100
76
0.02

Fig S5 (a-f) ML phylogenies of six individual nuclear markers (β-tubulin, DEAD, EFα, , IGS, ITS and RPB2) of 50 samples of *Thamnolia* collected from the northern hemisphere. Each figure depicts one individual ML gene tree. The IDs of the samples show their provenience and are colored according to lineage: A (blue), B (green) and C (purple). The samples from which we used genetic information from the draft genomes are shown in black letters. The tree is rooted with the three outgroups: *Dibaeis baeomyces, Siphula ceratites* and *Icmadophila ericetorum*. Bootstrap values are shown on the branches. The scale bar indicates the branch lengths.

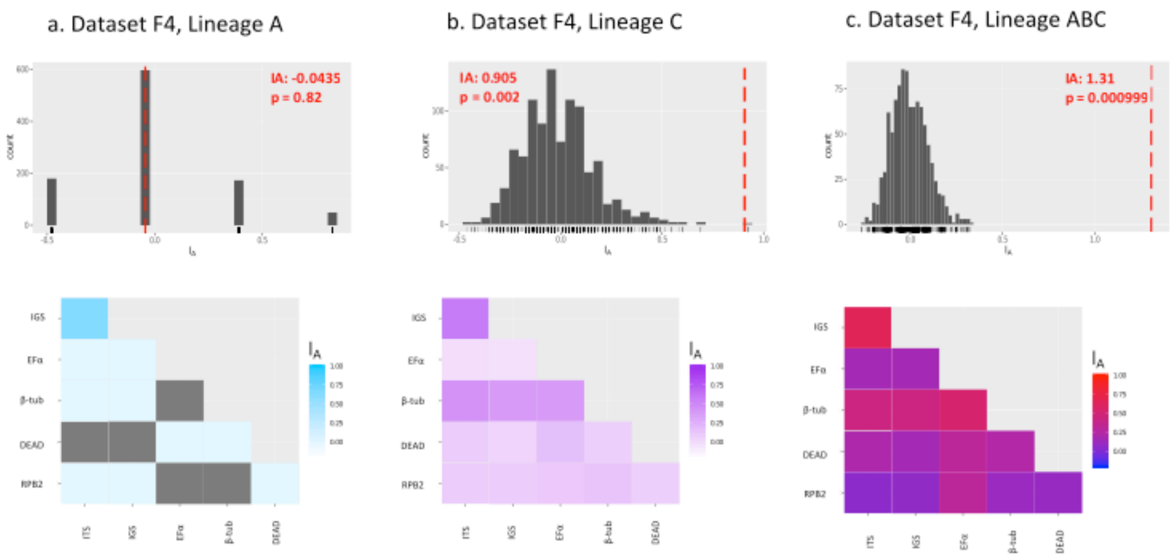

Fig. S6 The Index of Association (I_A) estimated on the dataset that contains genetic information from six fungal nuclear markers: ITS, IGS, EFα, DEAD, RPB2 and β-tubulin, in a total of 50 samples of *Thamnolia* collected predominantly from Scandinavia. I_A was calculated over all loci (histograms) and in a pairwise manner among all loci (heat maps). The gray histogram bars show the expected variance of genetic differences between individuals, while the red dashed line shows the observed variance. The colors of the heat maps are scaled to values between 0 (unlinked loci) and 1 (linked loci). Dark gray squares in the heat maps indicate that I_A could not be calculated between a pair of markers. Bonferroni corrected *P*-values of $\leq$ 0.002 indicate linkage disequilibrium. The test could be applied only to individuals assigned to Lineage A where I_A was not significantly different from 0 (p= 0.82) suggesting unlinked loci, and to Lineage C where I_A was significantly different from 0 (p= 0.002) suggesting linked loci. When all the individuals from the three lineages were considered together, I_A was significantly different from 0 (p= 0.0009) suggesting linked loci.

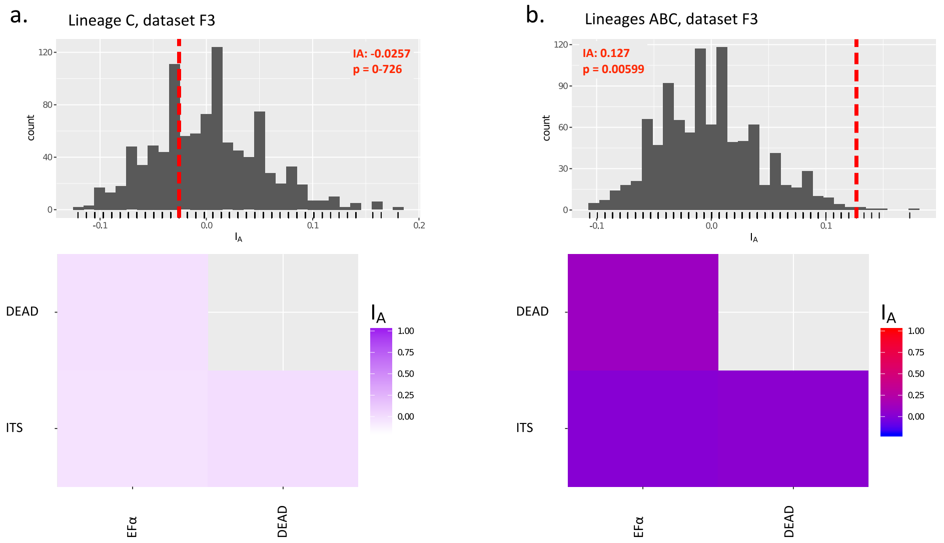

Fig. S7 The Index of Association (IA) for the Dataset F3 that uses genetic information from three fungal nuclear markers: ITS, EFα and DEAD, and a total of 129 samples collected across the Northern Hemisphere. I_A was calculated over all loci (histograms) and in a pairwise manner among all loci (heat maps). The gray histogram bars show the expected variance of genetic differences between individuals, and the red dashed line shows the observed variance. The colors of the heat maps are scaled to values between 0 (unlinked loci) and 1 (linked loci). Bonferroni corrected *P*-values of $\leq$ 0.008 indicate linkage disequilibrium, where the association between markers is significantly different than 0. The recombination test could be applied only to individuals belonging to Lineage C, where I_A was not significantly different from 0 (p= 0.726) suggesting linkage equilibrium. When all the individuals from the three lineages were considered together, I_A was significantly different from 0 (p= 0.0059) suggesting linked loci.

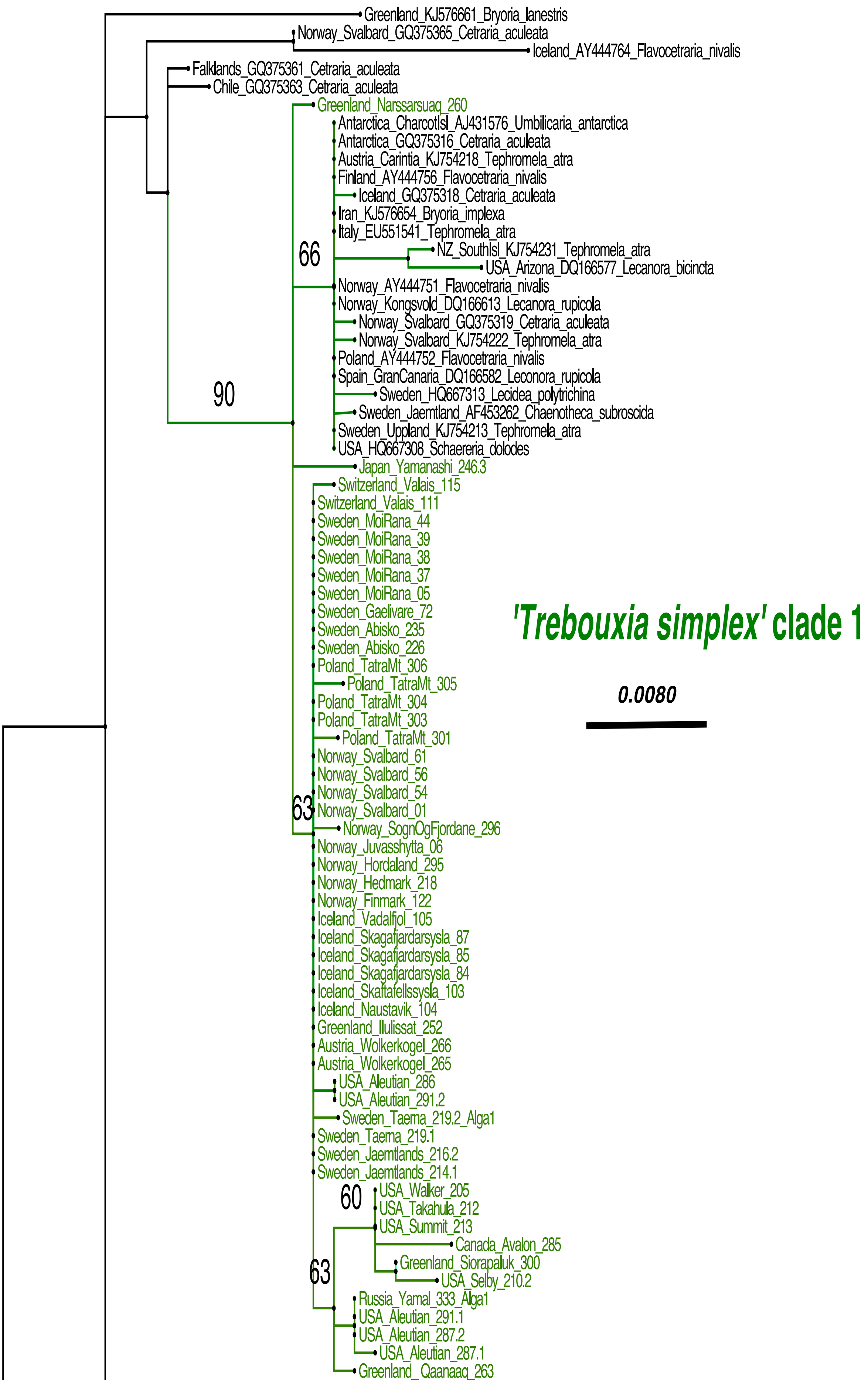

continue on next page

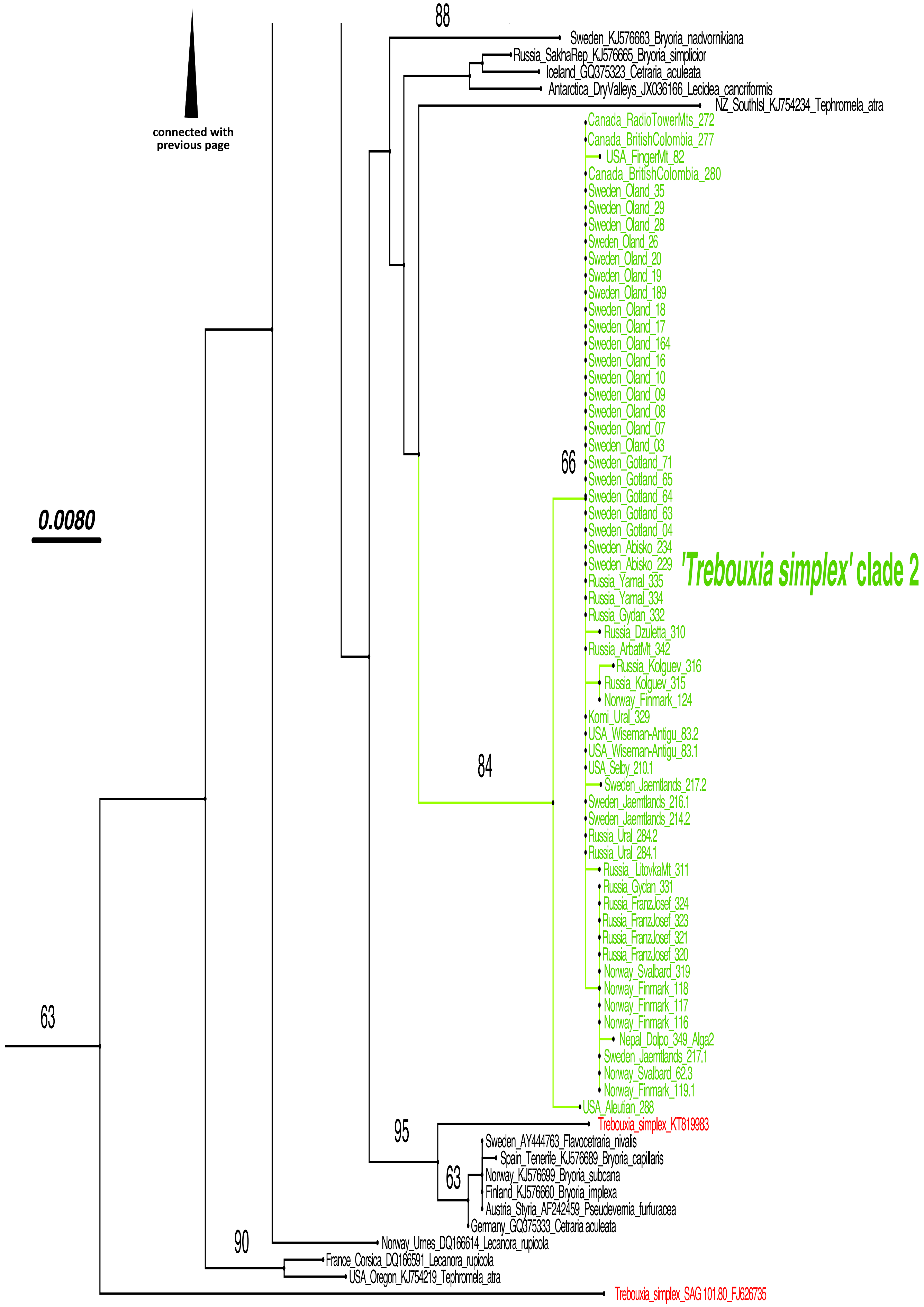
connected with previous page
0.0080
88
Sweden_KJ576663_Bryoria_nadvornikiana
Russia_SakhaRep_KJ576665_Bryoria_simplicior
Iceland_GQ375323_Cetraria_aculeata
Antarctica_DryValleys_JX036166_Lecidea_cancriformis
NZ_SouthIsl_KJ754234_Tephromela_atra
Canada_RadioTowerMts_272
Canada_BritishColombia_277
USA_FingerMt_82
Canada_BritishColombia_280
Sweden_Oland_35
Sweden_Oland_29
Sweden_Oland_28
Sweden_Oland_26
Sweden_Oland_20
Sweden_Oland_19
Sweden_Oland_189
Sweden_Oland_18
Sweden_Oland_17
Sweden_Oland_164
Sweden_Oland_16
Sweden_Oland_10
Sweden_Oland_09
Sweden_Oland_08
Sweden_Oland_07
Sweden_Oland_03
66
Sweden_Gotland_71
Sweden_Gotland_65
Sweden_Gotland_64
Sweden_Gotland_63
Sweden_Gotland_04
Sweden_Abisko_234
Sweden_Abisko_229
'Trebouxia simplex' clade 2
Russia_Yamal_335
Russia_Yamal_334
Russia_Gydan_332
Russia_Dzuletta_310
Russia_ArbatMt_342
Russia_Kolguev_316
Russia_Kolguev_315
Norway_Finmark_124
Komi_Ural_329
USA_Wiseman-Antigu_83.2
USA_Wiseman-Antigu_83.1
USA_Selby_210.1
84
Sweden_Jaemtlands_217.2
Sweden_Jaemtlands_216.1
Sweden_Jaemtlands_214.2
Russia_Ural_284.2
Russia_Ural_284.1
Russia_LitovkaMt_311
Russia_Gydan_331
Russia_FranzJosef_324
Russia_FranzJosef_323
Russia_FranzJosef_321
Russia_FranzJosef_320
Norway_Svalbard_319
Norway_Finmark_118
Norway_Finmark_117
Norway_Finmark_116
63
Nepal_Dolpo_349_Alga2
Sweden_Jaemtlands_217.1
Norway_Svalbard_62.3
Norway_Finmark_119.1
USA_Aleutian_288
95
Trebouxia_simplex_KT819983
Sweden_AY444763_Flavocetraria_nivalis
Spain_Tenerife_KJ576689_Bryoria_capillaris
63
Norway_KJ576699_Bryoria_subcana
Finland_KJ576660_Bryoria_implexa
Austria_Styria_AF242459_Pseudevernia_furfuracea
Germany_GQ375333_Cetraria aculeata
90
Norway_Umes_DQ166614_Lecanora_rupicola
France_Corsica_DQ166591_Lecanora_rupicola
USA_Oregon_KJ754219_Tephromela_atra
Trebouxia_simplex_SAG 101.80_FJ626735

Fig S8 Detailed resolution of the '*Trebouxia simplex* clade 1' and '*Trebouxia simplex* clade *2* from main Fig. 5. The photobionts amplified from *Thamnolia* are highlighted in dark green (clade 1) and light green (clade 2). GenBank photobiont sequences amplified from other lichens are in black and their ID includes information about origin, GenBank accession number, and species. The references photobiont sequences used for clade designation are highlighted in red. The scale bar indicates the branch lengths.

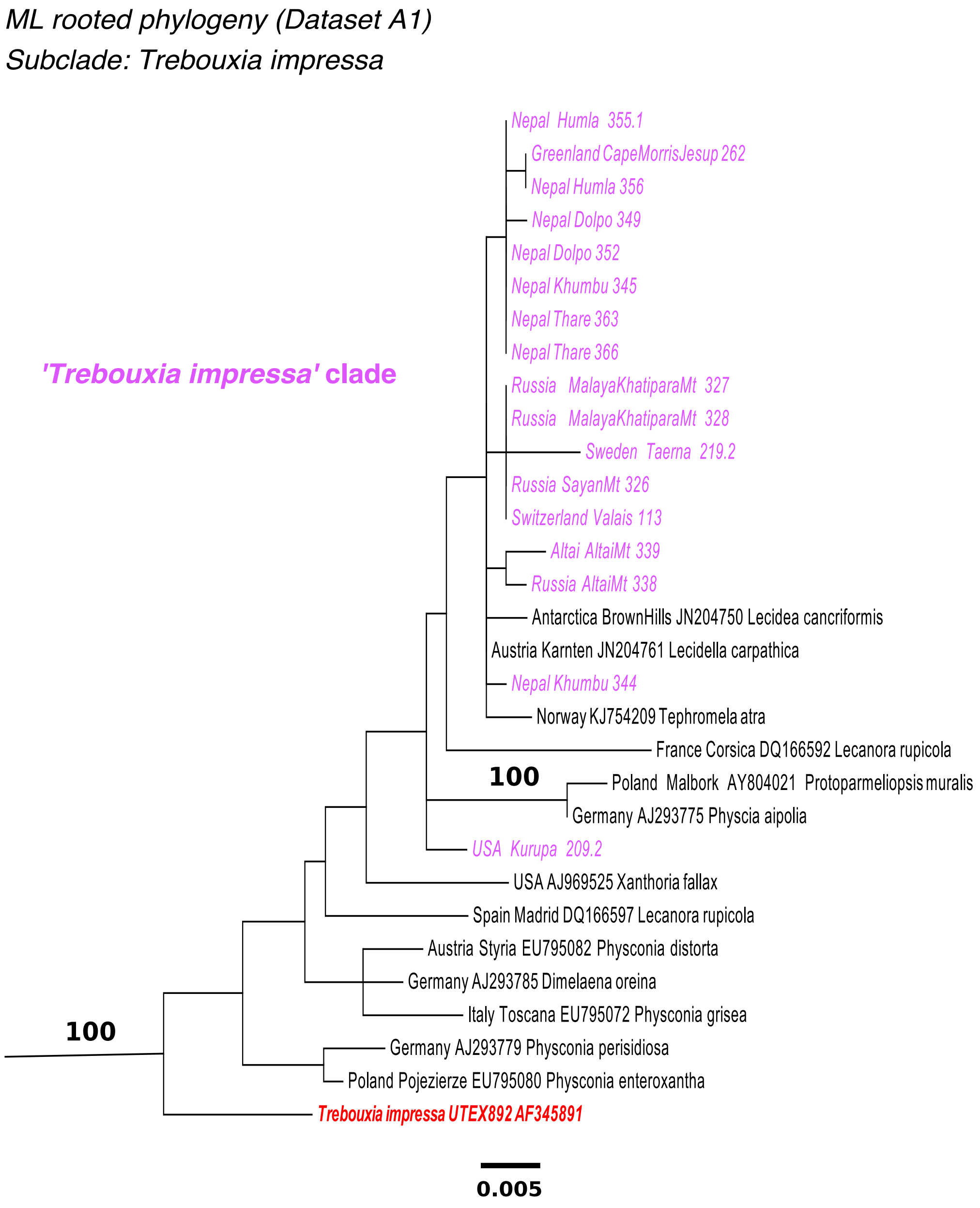

Fig S9 Detailed resolution of the '*Trebouxia impressa* clade' from main Fig. 5. The photobionts amplified from *Thamnolia* are highlighted in pink. The GenBank photobiont sequences amplified from other lichens are colored in black and their ID includes information about locality, the GenBank accession number, and the lichen species. The references photobiont sequence used for clade designation is highlighted in red. The scale bar indicates the branch lengths.

ML rooted phylogeny (Dataset A1)

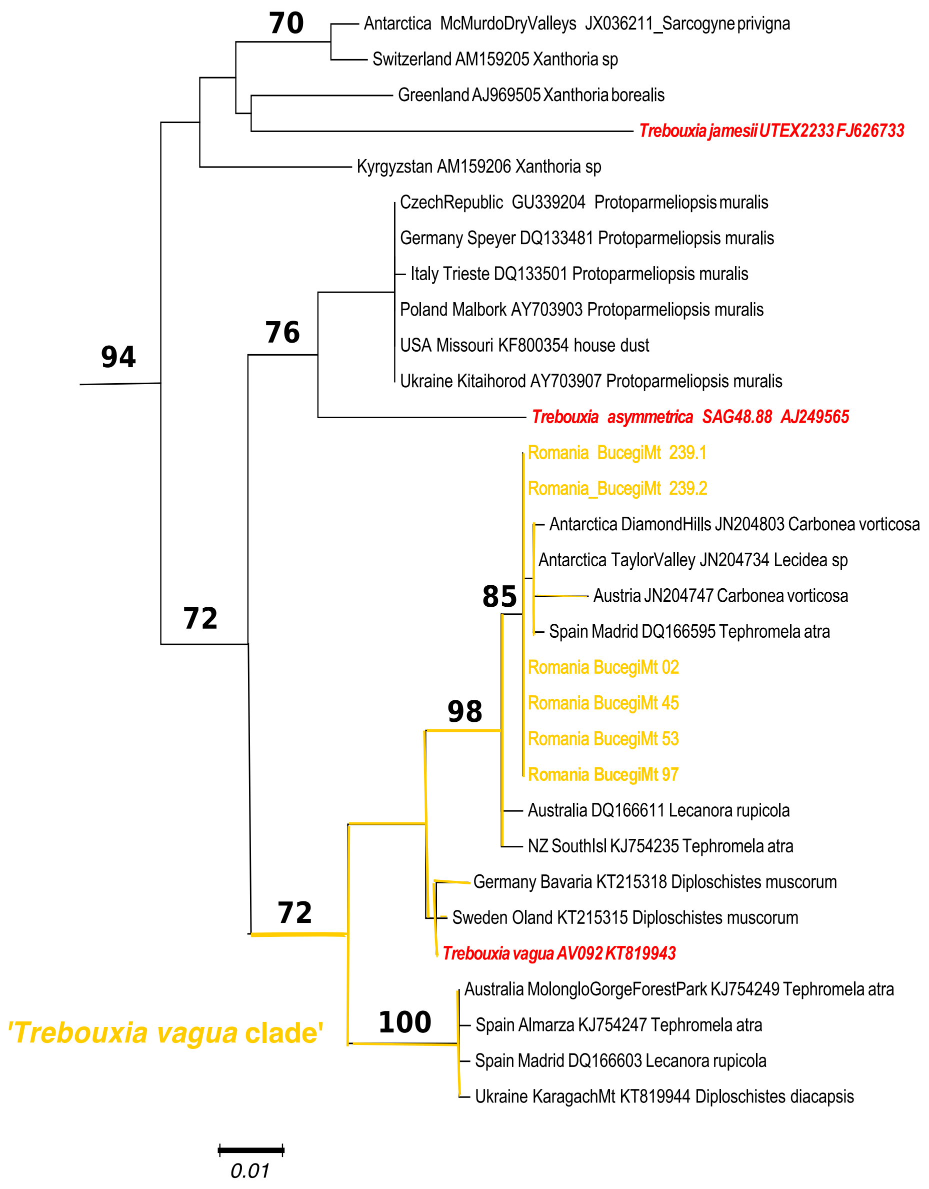

Fig S10 *Trebouxia vagua* clade. The figure shows in detail the *Trebouxia vagua* clade from the main Fig. 5. The photobionts amplified from *Thamnolia* are highlighted in orange. The GenBank photobiont sequences amplified from other lichens are in black and their ID includes information about origin, GenBank accession number, and species. The references photobiont sequences used for clade designation are highlighted in red. The scale bar indicates the branch lengths.

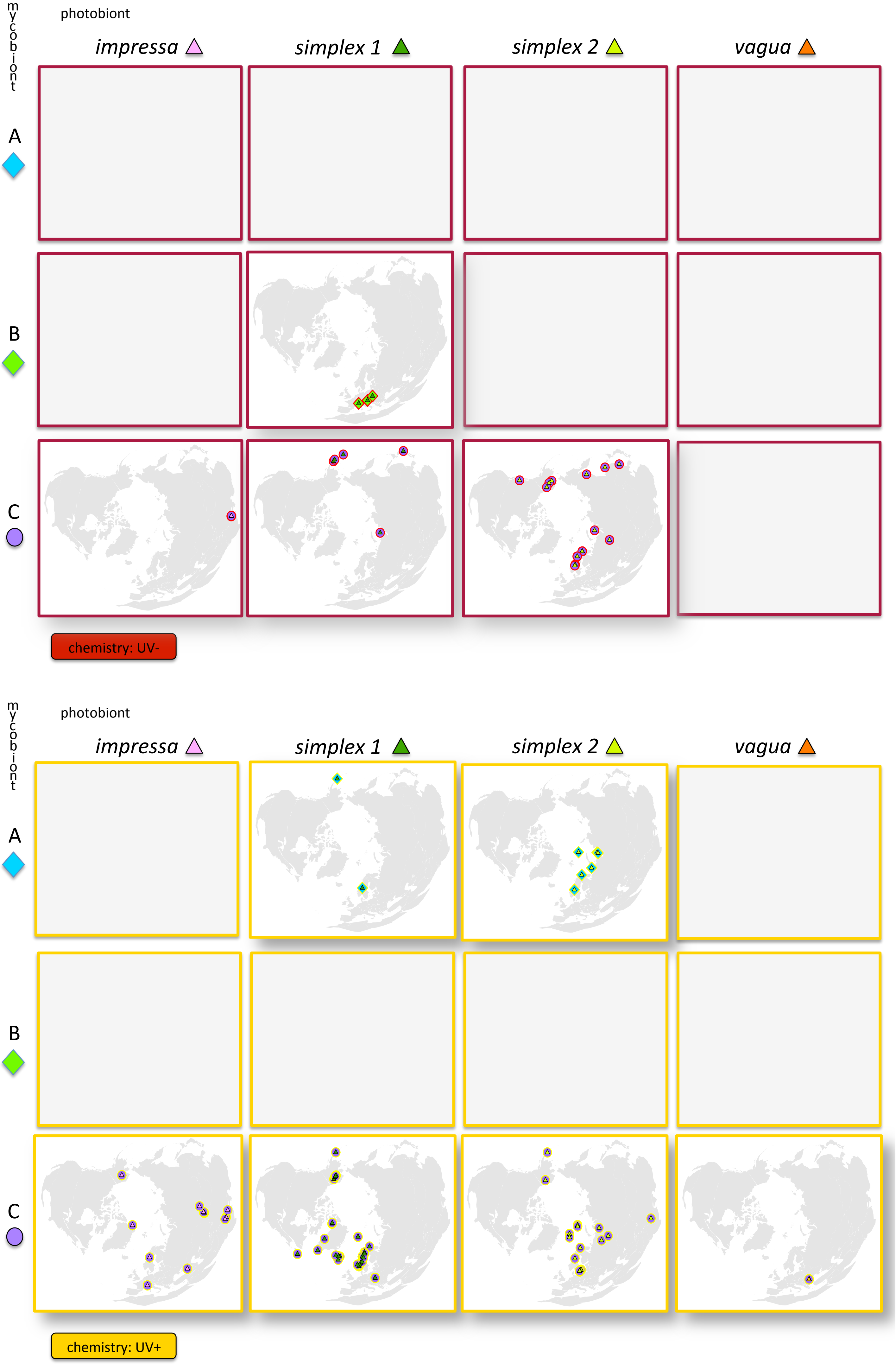

mycobiont
photobiont
impressa
simplex 1
simplex 2
vagua
A
B
C
chemistry: UV-
mycobiont
photobiont
impressa
simplex 1
simplex 2
vagua
A
B
C
chemistry: UV+

Fig S11 The range of photobionts associated with each mycobiont lineage and chemical phenotype. The figure depicts all possible combinations that may be formed by the three *T. vermicularis* mycobiont lineages (A: blue rhombs, B: green rhombs, and C: purple circles), the two sets of chemistries (UV+ highlighted in yellow frames and UV- as red frames) and all four *Trebouxia* photobiont lineages (*T. simplex* clade 1 as dark green triangles*, T. simplex* clade 2 as light green triangles*, T. vagua* clade as orange triangles, and *T. impressa* clade as pink triangles). The combinations observed are shown in distribution maps.

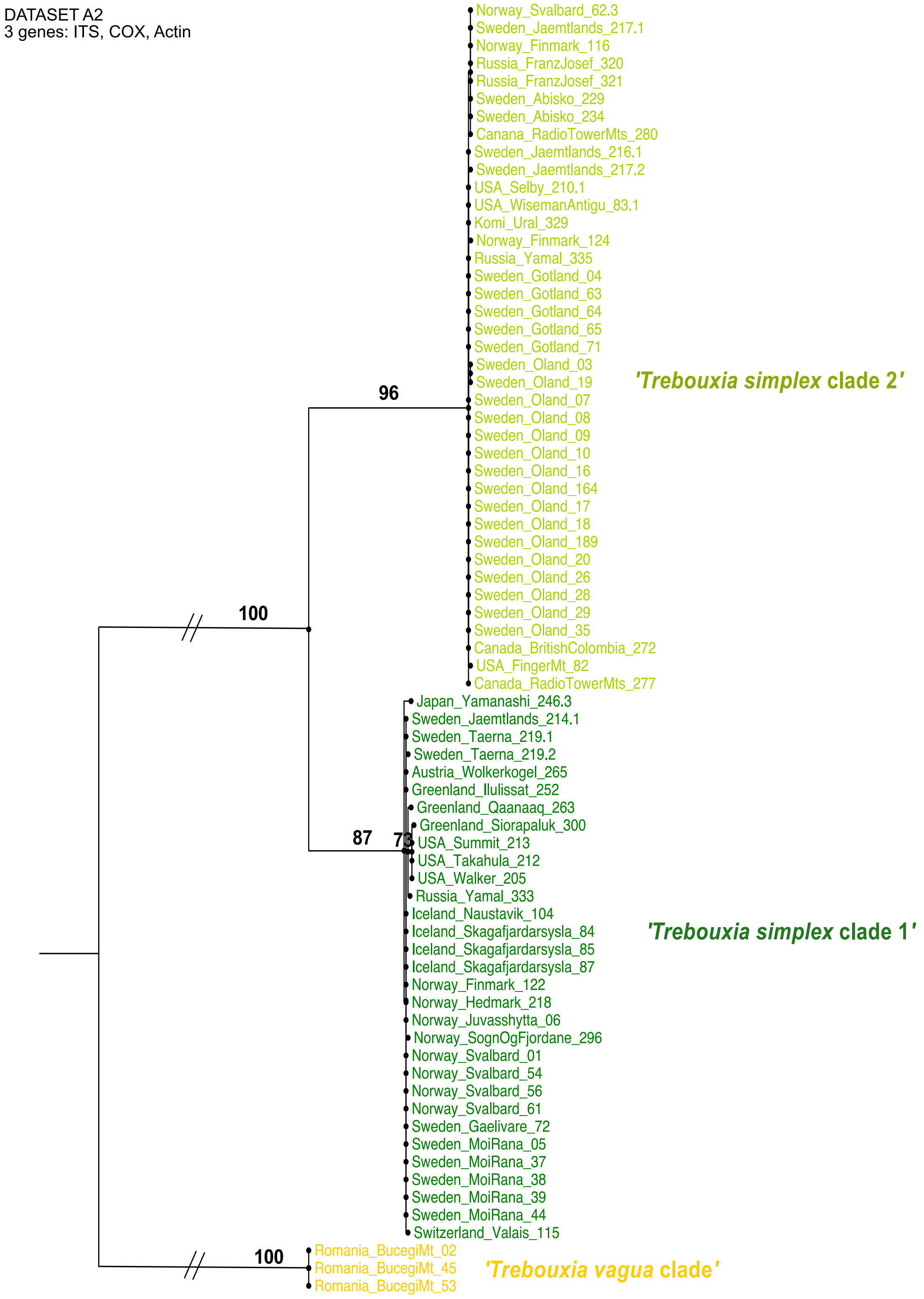

Fig S12 Rooted ML algal phylogeny of the concatenated sequences of three genes (Dataset A2) obtained from *Thamnolia*. The tree was rooted with the *Trebouxia vagua*. The scale bar indicates the branch lengths.
