# Supplementary material for: A worldwide phylogeography of the whiteworm lichens Thamnolia reveals three lineages with distinct habitats and evolutionary histories
Source: Ecol Evol. 2017 Apr 13;7(10):3602–15. doi: 10.1002/ece3.2917 (PMC5433967; doi:10.1002/ece3.2917)
Supplement: Supplementary file 2 [file ECE3-7-3602-s002.pdf]

B. Supporting Information (Tables)

Table S1. Summary of features traditionally used for taxon recognition in *Thamnolia*.
Thallus chemistry can be easily verified by exposure under UV light (3500 Å) where it shines brightly yellow (UV+) if it contains squamatic and baeomycesic acids and is dull red (UV-) if it contains thamnolic acid.

***Thamnolia* taxa**	**Podetium (thallus) chemistry**	**Podetium (thallus) morphology**	**References**
T. vermicularis var. *vermicularis*	thamnolic acid (UV-)	cylindrical and hollow	1
T. vermicularis var. *subuliformis*	squamatic and baeomycesic acid (UV+)	cylindrical and hollow	1
T. vermicularis unknown variety	unknown	cylindrical and hollow	1
T. papelillo var. *papelillo*	thamnolic acid (UV-)	flat and wide	2
T. papelillo var. *subsolida*	squamatic and baeomycesic acid (UV+)	flat and wide	2

[1]Culberson 1963, Nelsen & Gargas, 2009a; [2]Santesson, 2004

Table S2. Fungal samples and DNA sequences used in this study.

The fungal samples used in this study are divided into three groups. The first includes samples of *Thamnolia* for which new molecular markers were amplified and sequenced in this study; the second includes samples of *Dibaeis baeomyces, Icmadophila ericetorum, Siphula ceratites* and *Thamnolia sp.* for which we obtained the 6 nuclear markers from draft genomes; the third contains GenBank sequences from *Thamnolia* . The sample ID for *Thamnolia* samples is based on provenance and lab number (for newly generated sequences) or the accession number (for GenBank sequences). All samples that are notified as collected from discrete patches within at least one meter from each other are marked with 'transect' in the 'Sampling' section. For the samples marked as 'unknown' no information was provided. The table also provides for each sample the voucher and the collector information, together with podetium chemistry and morphology.

TOTAL	Sample ID	Sampling	Locality	Herbarium Voucher / culture collection	chemotype	morphology	collector
**Samples of *Thamnolia newly analyzed in this study***							
1	**Australia_AggieMt_554.3**	unknown	Brindabella Range, Mt. Aggie	CONN Plants of Australia, D.H. Vitt 816	UV-	cylindrical and hollow	D.H. Vitt
2	**Australia_AggieMt_567.1**	unknown	Brindabella Range, Mt. Aggie	UPS, Flora of Australia, Leif Tibell 11920	UV-	cylindrical and hollow	Leif Tibell
3	**Australia_AggieMt_567.2**	unknown	Brindabella Range, Mt. Aggie	UPS, Flora of Australia, Leif Tibell 11920	UV-	flat and wide	Leif Tibell
4	**Austria_SeetalerAlpen_427.1**	unknown	Seetaler Alpen	GZU, Walter Obermayer 13223	UV-	cylindrical and hollow	Walter Obermayer
5	**Austria_SeetalerAlpen_427.2**	unknown	Seetaler Alpen	GZU, Walter Obermayer 13223	UV-	cylindrical and hollow	Walter Obermayer
6	**Austria_SeetalerAlpen_428**	unknown	Seetaler Alpen	GZU, Walter Obermayer 13224	UV-	cylindrical and hollow	Walter Obermayer
7	**Austria_SeetalerAlpen_429**	unknown	Seetaler Alpen	GZU, Walter Obermayer 13225	UV-	cylindrical and hollow	Walter Obermayer
8	**Austria_SeetalerAlpen_430**	unknown	Seetaler Alpen	GZU, Walter Obermayer 13226	UV+	cylindrical and hollow	Walter Obermayer
9	**Austria_SeetalerAlpen_431**	unknown	Seetaler Alpen	GZU, Walter Obermayer 13227	UV+	cylindrical and hollow	Walter Obermayer
10	**Austria_SeetalerAlpen_432**	unknown	Seetaler Alpen	GZU, Walter Obermayer 13228	UV+	cylindrical and hollow	Walter Obermayer
11	**Austria_Wolkerkogel_265**	transect	Wölkerkogel	UPS, L-774097	UV-	cylindrical and hollow	Toby Spribille, Walter Obermayer
12	**Austria_Wolkerkogel_266**	transect	Wölkerkogel	UPS, L-774098	UV-	cylindrical and hollow	Toby Spribille, Walter Obermayer
13	**Bolivia_ApachetaChucura_381**	transect	Apacheta Chucura	UPS, L-774149	UV-	cylindrical and hollow	Eva Lievens
14	**Bolivia_ApachetaChucura_382**	transect	Apacheta Chucura	UPS, L-774150	UV-	cylindrical and hollow	Eva Lievens
15	**Bolivia_ApachetaChucura_383**	transect	Apacheta Chucura	UPS, L-774151	UV-	cylindrical and hollow	Eva Lievens
16	**Bolivia_LaCumbre_376**	transect	La Cumbre	UPS, L-774143	UV-	cylindrical and hollow	Eva Lievens
17	**Bolivia_LaCumbre_377**	transect	La Cumbre	UPS, L-774144	UV-	cylindrical and hollow	Eva Lievens
18	**Bolivia_LaCumbre_378**	transect	La Cumbre	UPS, L-774145	UV-	cylindrical and hollow	Eva Lievens
19	**Bolivia_LaCumbre_379**	transect	La Cumbre	UPS, L-774146	UV-	cylindrical and hollow	Eva Lievens
20	**Bolivia_LaCumbre_380**	transect	La Cumbre	UPS, L-774148	UV-	cylindrical and hollow	Eva Lievens

TOTAL	Sample ID	Sampling	Locality	Herbarium Voucher / culture collection	chemotype	morphology	collector
21	**Bolivia_SamanaPampa_386**	transect	Samana Pampa	UPS, L-774152	UV-	cylindrical and hollow	Eva Lievens
22	**Bolivia_SamanaPampa_387**	transect	Samana Pampa	UPS, L-774153	UV-	cylindrical and hollow	Eva Lievens
23	**Bolivia_SamanaPampa_388**	transect	Samana Pampa	UPS, L-774154	UV-	cylindrical and hollow	Eva Lievens
24	**Bolivia_SamanaPampa_389**	transect	Samana Pampa	UPS, L-774155	UV-	cylindrical and hollow	Eva Lievens
25	**Bolivia_SamanaPampa_390**	transect	Samana Pampa	UPS, L-774157	UV-	cylindrical and hollow	Eva Lievens
26	**Canada_Avalon_285**	unknown	Newfoundland, Avalon Peninsula	UPS, L-774107	UV+	cylindrical and hollow	Teuvo Ahti
27	**Canada_BritishColombia_272**	unknown	British Colombia, Radio Tower Mt	UPS, L-774099	UV-	cylindrical and hollow	Toby Spribille
28	**Canada_RadioTowerMt_277**	unknown	British Colombia, Radio Tower Mt	UPS, L-774100	UV-	cylindrical and hollow	Toby Spribille
29	**Canada_RadioTowerMt_280**	unknown	British Colombia, Radio Tower Mt	UPS, L-774103	UV-	cylindrical and hollow	Toby Spribille
30	**Chile_Brunswick_557.1**	transect	De Magallanes, Pen. de Brunswick	CONN BG no 5805	UV+	cylindrical and hollow	Bernard Goffinet
31	**Chile_Brunswick_557.2**	transect	De Magallanes, Pen. de Brunswick	CONN BG no 5805	UV+	cylindrical and hollow	Bernard Goffinet
32	**Chile_Brunswick_557.3**	transect	De Magallanes, Pen. de Brunswick	CONN BG no 5805	UV+	cylindrical and hollow	Bernard Goffinet
33	**Chile_Navarino_516.1**	transect	Navarino Island	CONN Flora of Chile, Bernard Goffinet 12919	UV-	cylindrical and hollow	Bernard Goffinet
34	**Chile_Navarino_516.2**	transect	Navarino Island	CONN Flora of Chile, Bernard Goffinet 12919	UV-	cylindrical and hollow	Bernard Goffinet
35	**Chile_Navarino_516.3**	transect	Navarino Island	CONN Flora of Chile, Bernard Goffinet 12919	UV-	cylindrical and hollow	Bernard Goffinet
36	**Chile_Navarino_556.3**	transect	Magallanes, Navarino Island	CONN, Flora of Chile, BG 6811	UV-	cylindrical and hollow	Bernard Goffinet & Cymion Cox
37	**China_Sichuan_566.1**	transect	Sichuan. Tibetan fringe, Shaluli Shan	UPS, Flora of China, W.O. 08447	UV-	cylindrical and hollow	Walter Obermayer
38	**China_Sichuan_566.2**	transect	Sichuan. Tibetan fringe, Shaluli Shan	UPS, Flora of China, W.O. 08447	UV-	cylindrical and hollow	Walter Obermayer
39	**Colombia_Boyara_473**	transect	Boyara, Cueva Grande	UPS, L-774170	UV-	cylindrical and hollow	Steve Behaeghel & Katrijn Van Nieuwenhuyse
40	**Colombia_Boyara_474**	transect	Boyara, Cueva Grande	UPS, L-774171	UV-	cylindrical and hollow	Steve Behaeghel & Katrijn Van Nieuwenhuyse
41	**Colombia_Boyara_476**	transect	Boyara, Cueva Grande	UPS, L-774172	UV-	cylindrical and hollow	Steve Behaeghel & Katrijn Van Nieuwenhuyse
42	**Colombia_LagoPanuelo_467**	transect	Lago Pañuelo	UPS, L-774167	UV-	cylindrical and hollow	Steve Behaeghel & Katrijn Van Nieuwenhuyse
43	**Colombia_LagoPanuelo_468**	transect	Lago Pañuelo	UPS, L-774168	UV-	cylindrical and hollow	Steve Behaeghel & Katrijn Van Nieuwenhuyse
44	**Colombia_LagoPanuelo_469**	transect	Lago Pañuelo	UPS, L-774169	UV-	cylindrical and hollow	Steve Behaeghel & Katrijn Van Nieuwenhuyse
45	**Falklands_KentMt_558.1**	unknown	East Falkland, Mt Kent	MSU, collection Alan Friday 10782a	UV-	cylindrical and hollow	Alan Friday
46	**Falklands_KentMt_558.3**	unknown	East Falkland, Mt Kent	MSU, collection Alan Friday 10782a	UV-	cylindrical and hollow	Alan Friday

TOTAL	Sample ID	Sampling	Locality	Herbarium Voucher / culture collection	chemotype	morphology	collector
47	**Falklands_MariaMt_559.1**	unknown	Port Howard, Mt Maria	MSU, collection Alan Friday 10916	UV-	cylindrical and hollow	Alan Friday
48	**Falklands_MariaMt_559.2**	unknown	Port Howard, Mt Maria	MSU, collection Alan Friday 10916	UV-	cylindrical and hollow	Alan Friday
49	**Falklands_MariaMt_559.3**	unknown	Port Howard, Mt Maria	MSU, collection Alan Friday 10916	UV-	cylindrical and hollow	Alan Friday
50	**Greenland_ Qaanaaq_263**	unknown	Qaanaaq	UPS L-617837	UV+	cylindrical and hollow	Eric Steen Hansen
51	**Greenland_CapeMorrisJesup_262**	unknown	Cape Morris Jesup	UPS L-617847	UV+	cylindrical and hollow	Eric Steen Hansen
52	**Greenland_Ilulissat_252**	unknown	Ilulissat, Jacobshavn	UPS L-617829	UV+	cylindrical and hollow	Eric Steen Hansen
53	**Greenland_Narssarsuaq_260**	unknown	Narssarsuaq	UPS L-617845	UV+	cylindrical and hollow	Eric Steen Hansen
54	**Greenland_Siorapaluk_300**	unknown	Siorapaluk	UPS, L-774108	UV+	cylindrical and hollow	Eric Steen Hansen
55	**Iceland_Naustavik_104**	unknown	Naustavik, S-Þingeyjarsysla	UPS, L-774061	UV+	cylindrical and hollow	Áugúst H. Bjarnason
56	**Iceland_Skaftafellssysla_103**	transect	Ljótarstaðaheiði, V-Skaftafellssysla	UPS, L-774060	UV+	cylindrical and hollow	Áugúst H. Bjarnason
57	**Iceland_Skagafjardarsysla_84**	transect	Skagafjardarsysla	UPS, L-774204	UV+	cylindrical and hollow	Starri Heidmarsson
58	**Iceland_Skagafjardarsysla_85**	transect	Skagafjardarsysla	UPS, L-774205	UV+	cylindrical and hollow	Starri Heidmarsson
59	**Iceland_Skagafjardarsysla_87**	transect	Skagafjardarsysla	UPS, L-774206	UV+	cylindrical and hollow	Starri Heidmarsson
60	**Iceland_Vadalfjol_105**	unknown	Vaðalfjöll, Austur-Barðastrandarsysla	UPS, L-774062	UV+	cylindrical and hollow	Áugúst H. Bjarnason
61	**Japan_282.B**	unknown	Mt. Tateyama	UPS, L-774104	UV-	cylindrical and hollow	unknown
62	**Japan_Yamanashi_246.3**	unknown	Yamanashi, Honshu, Kai Prov.	UPS L-593493	UV-	cylindrical and hollow	Göran Thor
63	**Komi_Ural_329**	unknown	Subpolar Urals, Yugid Va	UPS, L-774121	UV+	cylindrical and hollow	Natalia Semenova
64	**Nepal_Dolpo_349**	transect	Dolpo district	UPS, L-774130	UV+	cylindrical and hollow	Steve Behaeghel & Katrijn Van Nieuwenhuyse
65	**Nepal_Dolpo_352**	transect	Dolpo district	UPS, L-774132	UV+	cylindrical and hollow	Steve Behaeghel & Katrijn Van Nieuwenhuyse
66	**Nepal_Humla_355.1**	transect	Humla, Limi Valley, Nyula La Pass	UPS, L-774133	UV+	cylindrical and hollow	Steve Behaeghel & Katrijn Van Nieuwenhuyse
67	**Nepal_Humla_356**	transect	Humla, Limi Valley, Nyula La Pass	UPS, L-774134	UV+	cylindrical and hollow	Steve Behaeghel & Katrijn Van Nieuwenhuyse
68	**Nepal_Khumbu_344**	transect	Khumbu district	UPS, L-774128	UV+	cylindrical and hollow	Steve Behaeghel & Katrijn Van Nieuwenhuyse
69	**Nepal_Khumbu_345**	transect	Khumbu district	UPS, L-774129	UV-	cylindrical and hollow	Steve Behaeghel & Katrijn Van Nieuwenhuyse
70	**Nepal_Thare_363**	transect	Thare	UPS, L-774135	UV-	cylindrical and hollow	Steve Behaeghel & Katrijn Van Nieuwenhuyse
71	**Nepal_Thare_366**	transect	Thare	UPS, L-774136	UV-	cylindrical and hollow	Steve Behaeghel & Katrijn Van Nieuwenhuyse
72	**Norway_Finmark_116**	transect	Finmark County, Sør-Varanger	UPS, L-774066	UV-	cylindrical and hollow	Anders Larsson

TOTAL	Sample ID	Sampling	Locality	Herbarium Voucher / culture collection	chemotype	morphology	collector
73	**Norway_Finmark_117**	transect	Finmark County, Sør-Varanger	UPS, L-774067	UV+	cylindrical and hollow	Anders Larsson
74	**Norway_Finmark_118**	transect	Finmark County, Sør-Varanger	UPS, L-774067	UV-	cylindrical and hollow	Anders Larsson
75	**Norway_Finmark_119.1**	transect	Finmark County, Unjárga	Ioana Brännstrom 119	UV-	cylindrical and hollow	Anders Larsson
76	**Norway_Finmark_122**	transect	Finmark County, Krampenes	UPS, L-774068	UV+	cylindrical and hollow	Anders Larsson
77	**Norway_Finmark_124**	transect	Finmark County, Unjárga	UPS, L-774069	UV+	cylindrical and hollow	Anders Larsson
78	**Norway_Hedmark_218**	unknown	Hedmark County, Folldal	UPS, L-774088	UV+	cylindrical and hollow	Rui S. & Timdal E.
79	**Norway_Hordaland_295**	unknown	Hordaland County, Ulvik: Finse	HUO L-164260	UV+	cylindrical and hollow	Arne Pedersen
80	**Norway_Juvasshytta_06**	unknown	Oppland County, Juvasshytta	HUO, L-159902	UV+	cylindrical and hollow	Haugan R.
81	**Norway_SognOgFjordane_296**	unknown	Sogn og Fjordane, Helsethornet	HUO L-167260	UV+	cylindrical and hollow	Anders Breili
82	**Norway_Svalbard_01**	transect	Svalbard, Longyearbyen	UPS, L-774051	UV+	cylindrical and hollow	Beke Regelin
83	**Norway_Svalbard_319**	transect	Svalbard, Murchisonfjorden	UPS, L-774113	UV+	cylindrical and hollow	Liudmila Konoreva
84	**Norway_Svalbard_54**	transect	Svalbard, Longyearbyen	UPS, L-774194	UV+	cylindrical and hollow	Beke Regelin
85	**Norway_Svalbard_56**	transect	Svalbard, Longyearbyen	UPS, L-774195	UV+	cylindrical and hollow	Beke Regelin
86	**Norway_Svalbard_61**	transect	Svalbard, Longyearbyen	UPS, L-774196	UV+	cylindrical and hollow	Beke Regelin
87	**Norway_Svalbard_62.3**	transect	Svalbard, Longyearbyen	UPS, L-774197	UV+	cylindrical and hollow	Beke Regelin
88	**NZ_Cantebury_564.1**	unknown	Cantebury, Lake Lyndon	UPS, Flora of NZ, Leif Tibell 9733	UV-	cylindrical and hollow	Leif Tibell
89	**NZ_Cantebury_565.1**	unknown	Cantebury, NE Shore of Lake Lyndon	UPS, Flora of NZ, Leif Tibell 8972	UV-	cylindrical and hollow	Leif Tibell
90	**Peru_AlbodePucaraju_492**	transect	Albo de Pucaraju Pass	UPS, L-774181	UV+	flat and wide	Steve Behaeghel & Katrijn Van Nieuwenhuyse
91	**Peru_AlbodePucaraju_495**	transect	Albo de Pucaraju Pass	UPS, L-774182	UV-	cylindrical and hollow	Steve Behaeghel & Katrijn Van Nieuwenhuyse
92	**Peru_AlbodePucaraju_496**	transect	Albo de Pucaraju Pass	UPS, L-774183	UV-	cylindrical and hollow	Steve Behaeghel & Katrijn Van Nieuwenhuyse
93	**Peru_Ancash_395.2**	transect	Dept. Ancash	UPS, L-153301	UV-	flat and wide	R. Santensson & R. Moberg
94	**Peru_Bolognesi_393**	transect	Prov. Bolognesi	UPS, Flora of Peru, no. P53	UV+	flat and wide	R. Santensson & R. Moberg
95	**Peru_CordilleraBlanca_478**	transect	Cordillera Blanca, Vientunan Pass	UPS, L-774173	UV-	cylindrical and hollow	Steve Behaeghel & Katrijn Van Nieuwenhuyse
96	**Peru_CordilleraBlanca_479**	transect	Cordillera Blanca, Vientunan Pass	UPS, L-774174	UV-	cylindrical and hollow	Steve Behaeghel & Katrijn Van Nieuwenhuyse
97	**Peru_CordilleraBlanca_481**	transect	Cordillera Blanca, Vientunan Pass	UPS, L-774175	UV-	cylindrical and hollow	Steve Behaeghel & Katrijn Van Nieuwenhuyse
98	**Peru_Huaraz_394.2**	transect	Prov. Huaraz	UPS, Flora of Peru, n. P56:1	UV-	flat and wide	R. Santensson & R. Moberg

TOTAL	Sample ID	Sampling	Locality	Herbarium Voucher / culture collection	chemotype	morphology	collector
99	**Peru_Laguna69_369**	transect	Huaraz, Laguna 69	UPS, L-774137	ND	T. vermicularis	Eva Lievens
100	**Peru_Laguna69_370**	transect	Huaraz, Laguna 69	UPS, L-774139	ND	T. vermicularis	Eva Lievens
101	**Peru_Laguna69_371**	transect	Laguna 69	UPS, L-774140	ND	T. vermicularis	Eva Lievens
102	**Peru_Laguna69_372**	transect	Huaraz, Laguna 69	UPS, L-774141	ND	T. vermicularis	Eva Lievens
103	**Peru_Laguna69_373**	transect	Huaraz, Laguna 69	UPS, L-774142	UV-	cylindrical and hollow	Eva Lievens
104	**Peru_Laguna69_464**	transect	Laguna 69	UPS, L-774164	UV-	cylindrical and hollow	Eva Lievens
105	**Peru_Laguna69_465**	transect	Laguna 69	UPS, L-774165	UV-	cylindrical and hollow	Eva Lievens
106	**Peru_Laguna69_466**	transect	Laguna 69	UPS, L-774166	UV-	cylindrical and hollow	Eva Lievens
107	**Peru_LagunaSafunaBaja_482**	transect	Laguna Safuna Baja	UPS, L-774176	UV-	cylindrical and hollow	Steve Behaeghel & Katrijn Van Nieuwenhuyse
108	**Peru_LagunaSafunaBaja_484**	transect	Laguna Safuna Baja	UPS, L-774177	UV-	flat and wide	Steve Behaeghel & Katrijn Van Nieuwenhuyse
109	**Peru_LagunaSafunaBaja_485**	transect	Laguna Safuna Baja	UPS, L-774178	UV-	cylindrical and hollow	Steve Behaeghel & Katrijn Van Nieuwenhuyse
110	**Peru_Langoni_497**	transect	Langoni, Ishinca	UPS, L-774184	UV-	cylindrical and hollow	Steve Behaeghel & Katrijn Van Nieuwenhuyse
111	**Peru_Langoni_500**	transect	Langoni, Ishinca	UPS, L-774185	UV-	cylindrical and hollow	Steve Behaeghel & Katrijn Van Nieuwenhuyse
112	**Peru_Langoni_501**	transect	Langoni, Ishinca	UPS, L-774186	UV-	cylindrical and hollow	Steve Behaeghel & Katrijn Van Nieuwenhuyse
113	**Peru_QuebradaMatara_502**	transect	Quebrada Matara	UPS, L-774187	UV-	flat and wide	Steve Behaeghel & Katrijn Van Nieuwenhuyse
114	**Peru_QuebradaMatara_504**	transect	Quebrada Matara	UPS, L-774188	UV-	cylindrical and hollow	Steve Behaeghel & Katrijn Van Nieuwenhuyse
115	**Peru_QuebradaMatara_505**	transect	Quebrada Matara	UPS, L-774189	UV-	cylindrical and hollow	Steve Behaeghel & Katrijn Van Nieuwenhuyse
116	**Peru_Tarma_392.2**	transect	Prov. Tarma	UPS, Flora of Peru, 12th Regnellian Expedition, P26:2	UV+	flat and wide	R. Santensson & R. Moberg
117	**Peru_Tupatupa_507**	transect	Tupatupa	UPS, L-774190	UV-	cylindrical and hollow	Steve Behaeghel & Katrijn Van Nieuwenhuyse
118	**Peru_Tupatupa_508**	transect	Tupatupa	UPS, L-774191	UV-	cylindrical and hollow	Steve Behaeghel & Katrijn Van Nieuwenhuyse
119	**Peru_Tupatupa_511**	transect	Tupatupa	UPS, L-774192	UV-	cylindrical and hollow	Steve Behaeghel & Katrijn Van Nieuwenhuyse
120	**Peru_Yanapaccha_489**	transect	Yanapaccha Moraine Camp	UPS, L-774179	UV-	cylindrical and hollow	Steve Behaeghel & Katrijn Van Nieuwenhuyse
121	**Peru_Yanapaccha_491**	transect	Huascaran, Yanapaccha Moraine	UPS, L-774180	UV-	flat and wide	Steve Behaeghel & Katrijn Van Nieuwenhuyse
122	**Poland_TatraMt_301**	unknown	West Carpathians, Tatra Mts	KRAM-L 53024, voucher no 78	UV+	cylindrical and hollow	unknown
123	**Poland_TatraMt_303**	unknown	Tatra Mts., Żabi Szczyt Wyżni Mt	KRAM-L 53023, voucher no 80	UV+	cylindrical and hollow	unknown
124	**Poland_TatraMt_304**	unknown	Tatra Mts., Zawrat pass	KRAM-L 53022, voucher 81	UV+	cylindrical and hollow	unknown

TOTAL	Sample ID	Sampling	Locality	Herbarium Voucher / culture collection	chemotype	morphology	collector
125	**Poland_TatraMt_305**	unknown	Tatra Mts., Niżne Rysy Mt	KRAM-L 53021, herbarium voucher no 82	UV+	cylindrical and hollow	unknown
126	**Poland_TatraMt_306**	unknown	Tatra Mts., Wyżnia Spadowa Przełączka	KRAM-L 53027, herbarium voucher no 83	UV-	cylindrical and hollow	unknown
127	**Romania_BucegiMt_02**	transect	Sinaia, Bucegi Mts, Cota 2000	UPS, L-774052	UV+	cylindrical and hollow	Ioana Brännström & Lars Brännström
128	**Romania_BucegiMt_239.1**	transect	Bucegi Mts, Caraiman cottage	UPS, L-774094	UV+	cylindrical and hollow	Doina Iordachescu
129	**Romania_BucegiMt_239.2**	transect	Bucegi Mts, Caraiman cottage	UPS, L-774095	UV-	cylindrical and hollow	Doina Iordachescu
130	**Romania_BucegiMt_45**	transect	Sinaia, Bucegi Mts, Cota 2000	UPS, L-774162	UV+	cylindrical and hollow	Ioana Brännström & Lars Brännström
131	**Romania_BucegiMt_53**	transect	Sinaia, Bucegi Mts, Cota 2000	UPS, L-774193	UV+	cylindrical and hollow	Ioana Brännström & Lars Brännström
132	**Romania_BucegiMt_97**	transect	Bucegi Mts., Leaota pick	UPS, L-774207	UV+	cylindrical and hollow	Ioana Brännström & Lars Brännström
133	**Romania_RetezatMt_435**	transect	Portile Inchise, Retezat Mts	UPS, L-774159	UV+	cylindrical and hollow	Anca Dragu
134	**Romania_RetezatMt_449**	transect	Retezat Mts, Curmatura Bucurei	UPS, L-774161	UV+	cylindrical and hollow	Anca Dragu
135	**Romania_RetezatMt_463**	transect	Retezat Mts, Saua Judele	UPS, L-774163	UV-	cylindrical and hollow	Anca Dragu
136	**Russia_ LitovkaMt_311**	unknown	Shkotovskiy district, Litovka Mt.	UPS, L-774110	UV-	cylindrical and hollow	Lidia Yakovchenko
137	**Russia_ MalayaKhatiparaMt_327**	unknown	Malaya Khatipara Mt	UPS, L-774119	UV+	cylindrical and hollow	Mikhail P. Zhurbenko
138	**Russia_ MalayaKhatiparaMt_328**	unknown	Malaya Khatipara Mt	UPS, L-774120	UV+	cylindrical and hollow	Mikhail P. Zhurbenko
139	**Russia_AltaiMt_338**	transect	Distr. Kosch-Agac, Altai Mts	GZU, Herbarium Philipp Resl: 1136	UV+	cylindrical and hollow	Philipp Resl
140	**Russia_AltaiMt_339**	transect	Distr. Kosch-Agac, Altai Mts	UPS, L-774127	UV+	cylindrical and hollow	Philipp Resl
141	**Russia_ArbatMt_342**	unknown	Khabarovskiy Krai, Arbat Mt.	GZU, Toby Spribille 306963	UV-	cylindrical and hollow	Toby Spribille & L. Yakovchenko, C. Printzen, B. Kanz, E. Malashkina
142	**Russia_Dzuletta_310**	unknown	Dzuletta	UPS, L-774109	UV-	cylindrical and hollow	Lidia Yakovchenko
143	**Russia_FranzJosef_320**	unknown	Franz Josef Land, Champ Is., Cape Triest	UPS, L-774114	UV+	cylindrical and hollow	Sergei Kholod
144	**Russia_FranzJosef_321**	unknown	Franz Josef Land, Cape Tirol	UPS, L-774115	UV+	cylindrical and hollow	Sergei Kholod
145	**Russia_FranzJosef_323**	unknown	Franz Josef Land, Cape Rogatyi	UPS, L-774116	UV+	cylindrical and hollow	Sergei Kholod
146	**Russia_FranzJosef_324**	unknown	Franz Josef Land, Lovushka Bay	UPS, L-774117	UV+	cylindrical and hollow	Sergei Kholod
147	**Russia_Gydan_331**	unknown	Gydan peninsula	UPS, L-774122	UV+	cylindrical and hollow	S. Ektova, S. Abdulmanova, K. Ermokhina
148	**Russia_Gydan_332**	unknown	Gydan peninsula	UPS, L-774123	UV+	cylindrical and hollow	S. Ektova, S. Abdulmanova, K. Ermokhina
149	**Russia_Kolguev_315**	unknown	Kolguev Island	UPS, L-774111	UV+	cylindrical and hollow	Sergei Kholod

TOTAL	Sample ID	Sampling	Locality	Herbarium Voucher / culture collection	chemotype	morphology	collector
150	**Russia_Kolguev_316**	unknown	Kolguev Island	UPS, L-774112	UV+	cylindrical and hollow	Sergei Kholod
151	**Russia_SayanMt_326**	unknown	Krasnoyarsk Territory, Sayan Mts	UPS, L-774118	UV+	cylindrical and hollow	Mikhail P. Zhurbenko
152	**Russia_Ural_284.1**	unknown	Polar Urals, Mt. Bezymyannaya	UPS, L-774105	UV+	cylindrical and hollow	S. Ektova, S. Abdulmanova
153	**Russia_Ural_284.2**	unknown	Polar Urals, Mt. Bezymyannaya	UPS, L-774106	UV-	cylindrical and hollow	S. Ektova, S. Abdulmanova
154	**Russia_Yamal_333**	unknown	Yamal peninsula	UPS, L-774124	UV-	cylindrical and hollow	S. Ektova, S. Abdulmanova
155	**Russia_Yamal_334**	unknown	Yamal peninsula	UPS, L-774125	UV+	cylindrical and hollow	S. Ektova, S. Abdulmanova
156	**Russia_Yamal_335**	unknown	Yamal peninsula	UPS, L-774126	UV-	cylindrical and hollow	S. Ektova, S. Abdulmanova, K. Ermokhina
157	**Sweden_Abisko_226**	transect	Abisko, Njulla Peak	UPS, L-774090	UV+	cylindrical and hollow	Una Brännström
158	**Sweden_Abisko_229**	transect	Abisko, Njulla Peak	UPS, L-774091	UV-	cylindrical and hollow	Stela Onut, Gheorghe Onut
159	**Sweden_Abisko_234**	transect	Abisko, Njulla Peak	UPS, L-774092	UV-	cylindrical and hollow	Ioana Brännström
160	**Sweden_Abisko_235**	transect	Abisko, Njulla Peak	UPS, L-774093	UV+	cylindrical and hollow	Lars Brännström
161	**Sweden_Gaelivare_72**	transect	Lule Lappmark, Gälivare	HUP L-520827	UV+	cylindrical and hollow	Anders Nordin
162	**Sweden_Gotland_04**	transect	Gotland Island	UPS, L-774054	UV+	cylindrical and hollow	Ioana Brännström & Lars Brännström
163	**Sweden_Gotland_63**	transect	Gotland Island	UPS, L-774198	UV+	cylindrical and hollow	Ioana Brännström & Lars Brännström
164	**Sweden_Gotland_64**	transect	Gotland Island	UPS, L-774199	UV+	cylindrical and hollow	Ioana Brännström & Lars Brännström
165	**Sweden_Gotland_65**	transect	Gotland Island	UPS, L-774200	UV+	cylindrical and hollow	Ioana Brännström & Lars Brännström
166	**Sweden_Gotland_71**	transect	Gotland Island	UPS, L-774201	UV+	cylindrical and hollow	Ioana Brännström & Lars Brännström
167	**Sweden_Jaemtlands_214.1**	transect	Jämtlands County, Åre Municipality	UPS, L-774082	UV+	cylindrical and hollow	Anders Larsson
168	**Sweden_Jaemtlands_214.2**	transect	Jämtlands County, Åre Municipality	UPS, L-774083	UV-	cylindrical and hollow	Anders Larsson
169	**Sweden_Jaemtlands_216.1**	transect	Jämtlands County, Åre Municipality	UPS, L-774084	UV-	cylindrical and hollow	Anders Larsson
170	**Sweden_Jaemtlands_216.2**	transect	Jämtlands County, Åre Municipality	UPS, L-774085	UV+	cylindrical and hollow	Anders Larsson
171	**Sweden_Jaemtlands_217.1**	transect	Jämtlands County,Täljstensvalen	UPS, L-774086	UV+	cylindrical and hollow	Anders Larsson
172	**Sweden_Jaemtlands_217.2**	transect	Jämtlands County,Täljstensvalen	UPS, L-774087	UV-	cylindrical and hollow	Anders Larsson
173	**Sweden_MoiRana_05**	transect	Lappland, Mo i Rana pass	UPS, L-774055	UV+	cylindrical and hollow	Ioana Brännström & Lars Brännström
174	**Sweden_MoiRana_37**	transect	Lappland, Mo i Rana pass	UPS, L-774138	UV+	cylindrical and hollow	Ioana Brännström & Lars Brännström

TOTAL	Sample ID	Sampling	Locality	Herbarium Voucher / culture collection	chemotype	morphology	collector
175	**Sweden_MoiRana_38**	transect	Lappland, Mo i Rana pass	UPS, L-774147	UV+	cylindrical and hollow	Ioana Brännström & Lars Brännström
176	**Sweden_MoiRana_39**	transect	Lappland, Mo i Rana pass	UPS, L-774156	UV+	cylindrical and hollow	Ioana Brännström & Lars Brännström
177	**Sweden_MoiRana_44**	transect	Lappland, Mo i Rana pass	UPS, L-774160	UV+	cylindrical and hollow	Ioana Brännström & Lars Brännström
178	**Sweden_Öland_03**	transect	Öland, Stora Alvaret	UPS, L-774053	UV+	cylindrical and hollow	Ioana Brännström & Lars Brännström
179	**Sweden_Öland_07**	transect	Öland, Ehertop	UPS, L-774056	UV+	cylindrical and hollow	Amanda Brännström
180	**Sweden_Öland_08**	transect	Öland, Ehertop	UPS, L-774057	UV+	cylindrical and hollow	Noah Brännström
181	**Sweden_Öland_09**	transect	Öland, Ehertop	UPS, L-774058	UV+	cylindrical and hollow	Ioana Brännström & Lars Brännström
182	**Sweden_Öland_10**	transect	Öland, Ehertop	UPS, L-774059	UV+	cylindrical and hollow	Ioana Brännström & Lars Brännström
183	**Sweden_Öland_16**	transect	Öland, Ehertop	UPS, L-774070	UV+	cylindrical and hollow	Ioana Brännström & Lars Brännström
184	**Sweden_Öland_164**	transect	Öland, Stora Alvaret	UPS, L-774071	UV+	cylindrical and hollow	Ioana Brännström & Julia York
185	**Sweden_Öland_17**	transect	Öland, Stora Alvaret	UPS, L-774072	UV+	cylindrical and hollow	Ioana Brännström & Lars Brännström
186	**Sweden_Öland_18**	transect	Öland, Stora Alvaret	Ioana Brännström 18	UV+	cylindrical and hollow	Ioana Brännström & Lars Brännström
187	**Sweden_Öland_189**	transect	Öland, Grynge Natural Reserve	UPS, L-774073	UV+	cylindrical and hollow	Ioana Brännström & Lars Brännström
188	**Sweden_Öland_19**	transect	Öland, Stora Alvaret	UPS, L-774074	UV+	cylindrical and hollow	Ioana Brännström & Lars Brännström
189	**Sweden_Öland_20**	transect	Öland, Stora Alvaret	UPS, L-774075	UV+	cylindrical and hollow	Ioana Brännström & Lars Brännström
190	**Sweden_Öland_26**	transect	Öland, Stora Alvaret	UPS, L-774096	UV+	cylindrical and hollow	Ioana Brännström & Lars Brännström
191	**Sweden_Öland_28**	transect	Öland, Stora Alvaret	UPS, L-774101	UV+	cylindrical and hollow	Ioana Brännström & Lars Brännström
192	**Sweden_Öland_29**	transect	Öland, Stora Alvaret	UPS, L-774102	UV+	cylindrical and hollow	Ioana Brännström & Lars Brännström
193	**Sweden_Öland_35**	transect	Öland, Stora Alvaret	UPS, L-774131	UV+	cylindrical and hollow	Ioana Brännström & Lars Brännström
194	**Sweden_Taerna_219.1**	transect	Lappland, Tärna, Strokittelhoben	UPS, L-774089	UV+	cylindrical and hollow	Mats Thulin
195	**Sweden_Taerna_219.2**	transect	Lappland, Tärna, Strokittelhoben	UPS, L-774089	UV+	cylindrical and hollow	Mats Thulin
196	**Switzerland_SchynigePlatteau_433**	unknown	Botanischer Schynige Platteau	UPS, L-774158	UV-	cylindrical and hollow	Loredana Ament
197	**Switzerland_Valais_111**	unknown	Valais, Col des Gentianes	UPS, L-774063	UV-	cylindrical and hollow	Paul Diederich
198	**Switzerland_Valais_113**	unknown	Valais, Col des Gentianes	UPS, L-774064	UV+	cylindrical and hollow	Paul Diederich
199	**Switzerland_Valais_115**	unknown	Valais, Col des Gentianes	UPS, L-774065	UV-	cylindrical and hollow	Paul Diederich
200	**USA_Aleutian_286**	unknown	Aleutian Islands, Amila Island	AKFWS, BM Helsinki #AML 86	UV-	cylindrical and hollow	Stephan S. Talbot

TOTAL	Sample ID	Sampling	Locality	Herbarium Voucher / culture collection	chemotype	morphology	collector
201	**USA_Aleutian_287.1**	unknown	Aleutian Islands, Unimak Issland	BMH Teuvo Ahti 700023	UV-	cylindrical and hollow	Teuvo Ahti & Stephan S. Talbot
202	**USA_Aleutian_287.2**	unknown	Aleutian Islands, Unimak Issland	BMH Teuvo Ahti 700023	UV+	cylindrical and hollow	Teuvo Ahti & Stephan S. Talbot
203	**USA_Aleutian_288**	unknown	Aleutian Islands, Unimak Issland	AKFWS, BM Helsinki #UNI034-24	UV+	cylindrical and hollow	Stephan S. Talbot & John Mayers
204	**USA_Aleutian_291.1**	unknown	Aleutian Islands, Unimak Issland	AKFWS, BM Helsinki #TIG003-X-1A	UV-	cylindrical and hollow	Stephan S. Talbot & W.B. Schofield
205	**USA_Aleutian_291.2**	unknown	Aleutian Islands, Tigalda Island	AKFWS, BM Helsinki #TIG003-X-1A	UV+	cylindrical and hollow	Stephan S. Talbot & W.B. Schofield
206	**USA_FingerMt_82**	unknown	Finger Mt	UPS, L-774202	UV-	cylindrical and hollow	Sanja Savic
207	**USA_Kurupa_209.2**	unknown	Kurupa Lake	UPS, L-774077	UV+	cylindrical and hollow	Peter Nellson
208	**USA_Selby_210.1**	unknown	Selby and Narvak Lakes	UPS, L-774078	UV-	cylindrical and hollow	Peter Nellson
209	**USA_Selby_210.2**	unknown	Selby and Narvak Lakes	UPS, L-774079	UV+	cylindrical and hollow	Peter Nellson
210	**USA_Summit_213**	unknown	Summit Lake	UPS, L-774081	UV+	cylindrical and hollow	Peter Nellson
211	**USA_Takahula_212**	unknown	Takahula Lake	UPS, L-774080	UV+	cylindrical and hollow	Peter Nellson
212	**USA_Walker_205**	unknown	Walker Lake	UPS, L-774076	UV+	cylindrical and hollow	Peter Nellson
213	**USA_Wiseman-Antigu_83.1**	unknown	Wiseman-Antigu Pass to Galbreat L.	UPS, L-774203	UV-	cylindrical and hollow	Sanja Savic
214	**USA_Wiseman-Antigu_83.2**	unknown	Wiseman-Antigu Pass to Galbreat L.	UPS, L-774203	UV+	cylindrical and hollow	Sanja Savic
**Nuclear markers extracted from draft genomes**							
215	***Thamnolia sp.***	unknown	Japan, Mt. Tateyama	Fungal culture #0043M (Laboratory of Advanced Bio-Production Science, Akita Prefectural University)	UV-	cylindrical and hollow	Yoshikasu Yamamoto
216	***Dibaeis baeomyces***	unknown	unknown	Fungal culture #0196M (Laboratory of Advanced Bio-Production Science, Akita Prefectural University)	ND	Dibaeis baeomyces	Yoshikasu Yamamoto
217	***Icmadophila ericetorum***	unknown	Austria, Gurktaler Alpen, Hochrind	Fungal culture #05.06.13 (Institute of Plant Sciences,vUniversity of Graz)	ND	Icmadophila ericetorum	Philipp Resl
218	***Siphula ceratites***	unknown	Norway, Rogaland	Fungal culture BG-L-95765	ND	Siphula ceratites	Tor Tønsberg
**GenBank sequences**							
219	**Australia_NewSouthWales_AY961602**	unknown	New South Wales	other studies	UV-	cylindrical and hollow	other studies
220	**Australia_NewSouthWales_AY961603**	unknown	New South Wales	other studies	ND	Thamnolia sp.	other studies
221	**Canada_PlateauMt_JQ409348**	unknown	Alberta, Mt. Plateau	other studies	UV+	cylindrical and hollow	other studies
222	**Canada_RaeMt_JQ409346**	unknown	Mt. Rae	other studies	UV+	cylindrical and hollow	other studies
223	**China_Yunnan_EU714432**	unknown	Yunnan	other studies	UV+	cylindrical and hollow	other studies

TOTAL	Sample ID	Sampling	Locality	Herbarium Voucher / culture collection	chemotype	morphology	collector
224	**China_Yunnan_EU714433**	unknown	Yunnan	other studies	UV+	cylindrical and hollow	other studies
225	**China_Yunnan_EU714436**	unknown	Yunnan	other studies	UV-	cylindrical and hollow	other studies
226	**China_Yunnan_EU714437**	unknown	Yunnan	other studies	UV-	cylindrical and hollow	other studies
227	**CostaRica_SanJose_EU714434**	unknown	San Jose	other studies	UV-	cylindrical and hollow	other studies
228	**CostaRica_SanJose_EU714435**	unknown	San Jose	other studies	UV-	cylindrical and hollow	other studies
229	**Norway_Troms_EU714430**	unknown	Norway, Troms	other studies	UV+	cylindrical and hollow	other studies
230	**Norway_Troms_EU714431**	unknown	Norway, Troms	other studies	UV+	cylindrical and hollow	other studies
231	**NZ_AY961604**	unknown	New Zeeland	other studies	UV+	cylindrical and hollow	other studies
232	**NZ_AY961605**	unknown	New Zeeland	other studies	UV+	cylindrical and hollow	other studies
233	**Peru_Cusco_JQ409343**	unknown	Cusco	other studies	UV-	cylindrical and hollow	other studies
234	**Peru_Cusco_JQ409349**	unknown	Cusco	other studies	UV+	cylindrical and hollow	other studies
235	**USA_Aleutian_EU714413**	unknown	Aleutian Islands, Kiska Island	other studies	UV+	cylindrical and hollow	other studies
236	**USA_Aleutian_EU714414**	unknown	Aleutian Islands, Kiska Island	other studies	UV-	cylindrical and hollow	other studies
237	**USA_Aleutian_EU714415**	unknown	Aleutian Islands, Kiska Island	other studies	UV-	cylindrical and hollow	other studies
238	**USA_Aleutian_EU714416**	unknown	Aleutian Islands, Kiska Island	other studies	UV+	cylindrical and hollow	other studies
239	**USA_Aleutian_EU714417**	unknown	Aleutian Islands, Kiska Island	other studies	UV-	cylindrical and hollow	other studies
240	**USA_Aleutian_EU714418**	unknown	Aleutian Islands, Kiska Island	other studies	UV+	cylindrical and hollow	other studies
241	**USA_Aleutian_EU714419**	unknown	Aleutian Islands, Adugak Island	other studies	UV-	cylindrical and hollow	other studies
242	**USA_Aleutian_EU714420**	unknown	Aleutian Islands, Chagulak Island	other studies	UV-	cylindrical and hollow	other studies
243	**USA_Aleutian_EU714421**	unknown	Aleutian Islands, Chagulak Island	other studies	UV+	cylindrical and hollow	other studies
244	**USA_Aleutian_EU714422**	unknown	Aleutian Islands, Nizki Island	other studies	UV+	cylindrical and hollow	other studies
245	**USA_Aleutian_EU714423**	unknown	Aleutian Islands, Nizki Island	other studies	UV+	cylindrical and hollow	other studies
246	**USA_Aleutian_EU714424**	unknown	Aleutian Islands, Little Kiska Island	other studies	UV+	cylindrical and hollow	other studies
247	**USA_Aleutian_EU714427**	unknown	Aleutian Islands, Ogangen Island	other studies	UV-	cylindrical and hollow	other studies
248	**USA_Aleutian_EU714428**	unknown	Aleutian Islands, Ogangen Island	other studies	UV+	cylindrical and hollow	other studies
249	**USA_Aleutian_EU714429**	unknown	Aleutian Islands, Ogangen Island	other studies	UV-	cylindrical and hollow	other studies

TOTAL	Sample ID	Sampling	Locality	Herbarium Voucher / culture collection	chemotype	morphology	collector
250	**USA_Chugarch_JQ409342**	unknown	Chugach State Park, Anchorage	other studies	UV+	cylindrical and hollow	other studies
251	**USA_Chugarch_JQ409344**	unknown	Chugach State Park, Anchorage	other studies	UV-	cylindrical and hollow	other studies
252	**USA_Kinnikinnik_JQ409345**	unknown	Kinnikinnik	other studies	UV-	cylindrical and hollow	other studies
253	**USA_Kinnikinnik_JQ409347**	unknown	Kinnikinnik	other studies	UV+	cylindrical and hollow	other studies

UV + samples contain baeomycesic and squamatic acids; UV- samples contain thamnolic acid; ND= samples are podetia for which the chemotype was not determined.

Table S3. Primers used for this study.

A total of 6 nuclear markers were amplified for the mycobiont of *Thamnolia*: β-tubulin (β-tub), dead-box helicase (DEAD), translation elongation factor 1-alpha gene (EFα), intergenic spacer region (IGS), internal transcribed spacer (ITS), RNA polymerase II core subunit (RPB2). For the photobiont we used three markers: two nuclear regions (ITS and actin) and one mitochondrial (Cytochrome c oxidase subunit I, COX). The table shows which primer combination(s) were used for each marker, the primer name, the primer sequence, the degree of specificity for each organism and in which study (References) the primers were suggested.

Organism	Marker	Type	Primer name	Primersequence(5'-3')	Specificity	References
MYCOBIONT	β-tub	nuclear	Bt2a	TTCCCCCGTCTCCACTTCTTCATG	Mycobiont	Glass & Donaldson, 1995
		nuclear	Bt2b	GACGAGATCGTTCATGTTGAACTC	Mycobiont	Glass & Donaldson, 1995
	DEAD	nuclear	X-geneFor1	CGCAATTAGTTGGGGGTA	*Thamnolia*	designed for this study
		nuclear	X-geneRev2	CGAGGTAGCGATAGACCA	*Thamnolia*	designed for this study
	EF	nuclear	EF1-983F	GCYCCYGGHCAYCGTGAYTTYAT	Mycobiont	http://www.aftol.org/pdfs/EF1primer.pdf
		nuclear	EF1-1576R	ACHGTRCCRATACCACCRATCTT	Mycobiont	http://www.aftol.org/pdfs/EF1primer.pdf
			EF_Th_F1	TCTTGTAGACATCCTGGAGA	*Thamnolia*	designed for this study
			EF_Th_R2	AGAACATGATTACTGGTACCTCCC	*Thamnolia*	designed for this study
	IGS	nuclear	IGS12a	AGTCTGTGGATTAGTGGCCG	Mycobiont	Carbone & Kohn, 1999
		nuclear	NS1R	GAGACAAGCATATGACTAC	Mycobiont	Carbone & Kohn, 1999
		nuclear	Thamnolia_IGSfor_pp2	ATGGCCATCGCTACGTA	Thamnolia	designed for this study
		nuclear	Thamnolia_IGSrev_pp2	ATGACTACTGGCAGA	Thamnolia	designed for this study
	ITS	nuclear	ITS 1F	CTTGGTCATTTAGAGGAAGTAA	Mycobiont	Gardes & Bruns 1991
		nuclear	ITS 4	TCCTCCGCTTATTGATATGC	Mycobiont	Gardes & Bruns 1991
		nuclear	ITS2	GCTGCGTTCTTCATCGATGC	Mycobiont	Gardes & Bruns 1991
		nuclear	ITS3	GCATCGATGAAGAACGCAGC	Mycobiont	Gardes & Bruns 1991
		nuclear	Thamn_ITSf_for1	TCCTTCCGCGGTGAAGCTA	*Thamnolia*	designed for this study
		nuclear	Thamn_ITSf_for2	GAAGCTACCGAAGCCTTTGC	*Thamnolia*	designed for this study
		nuclear	Thamn_ITSf_rev1	ATGCTTAAGTTCAGCGGGTA	*Thamnolia*	designed for this study
		nuclear	Thamn_ITSf_rev2	CCTACCTGATCCGAGGTCAA	*Thamnolia*	designed for this study
		nuclear	Th_anc_F1	TGGTATCTCGTGCGAAAGAC	*Thamnolia*	designed for this study
		nuclear	Th_anc_R1	CGTATCCTGGGACTTAGAGC	*Thamnolia*	designed for this study
		nuclear	Th_anc_F2	AGCGAAGGCTCTAAGTCCCA	*Thamnolia*	designed for this study
		nuclear	Th_anc_R2	CTTCCGCAGGTTCACCTACG	*Thamnolia*	designed for this study
		nuclear	Th_anc_F3	GAACTGTTACCCGGTTGCTT	*Thamnolia*	designed for this study
		nuclear	Th_anc_R3	GCATTTCGCTGCGTTCTTCA	*Thamnolia*	designed for this study
	RPB2	nuclear	RPB2-A-MNTH	TGGCAAGAAGCGACTGGAT	Mycobiont	Nelsen & Gargas, 2009
		nuclear	RPB2-B-MNTH	ATCGGCTGGGAGGTCTTTGTC	Mycobiont	Nelsen & Gargas, 2009
PHOTOBIONT	Actin	nuclear	ACT1 T	CACACRGTRCCCATCTAYGAGG	Photobiont	Kroken & Taylor, 2000
		nuclear	ACT2 T	AGGTAGCTCATAGTTCTTCTCAAT	Photobiont	Kroken & Taylor, 2000
		nuclear	ACT3T	ACYATTGAGAAGAACTATGAGCTA	Photobiont	Kroken & Taylor, 2000
		nuclear	ACT4T	GTTGAACAGCACCTCAGGGCA	Photobiont	Kroken & Taylor, 2000
	COX2	mithocondrial	Cox2-P2fw-5'	GGCATGAAAGCATGGTTAGC	Photobiont	Fernandez-Mendoza et al, 2011
		mithocondrial	Cox2-P2rv-3'	TCTGGATGTTAGCAAGAACTTTGT	Photobiont	Fernandez-Mendoza et al, 2011
	ITS	nuclear	ITS 1T	GAAGGATCATTGAATCTATCGT	Photobiont	Kroken & Taylor 2000
		nuclear	ITS 4T	GGTTCGCTCGCCGCTACTA	Photobiont	Kroken & Taylor 2000
		nuclear	ITS3T	AACGATGAAGAACGCAGCGAA	Photobiont	Kroken & Taylor 2000
		nuclear	ITS2T	TTCGCTGCGTTCTTCATCGTT	Photobiont	Kroken & Taylor 2000
		nuclear	ITS2T_Thamn	GGAATTCTGCAATTCACACT	*Thamnolia* photobiont	designed for this study
		nuclear	ITS1T_Thamn2	TGGGCGGGTCCAATAYCARY	*Thamnolia* photobiont	designed for this study
		nuclear	ITS1T_Thamn1	TTTCCGTAGGTGAACCTGCG	*Thamnolia* photobiont	designed for this study
		nuclear	ITS4T_Thamn1	CTTAAGTTCAGCGGGTGTTC	*Thamnolia* photobiont	designed for this study

Table S4. Photobiont GenBank sequences used for this study. When possible the ID of each sequence incorporates the name of the country/region of origin and the locality. The table shows: GenBank accession number; the taxon name as given by the authors of the sequence (GenBank species ID); the source of the photobiont - either from a lichen, an axenic algal culture, or an environmental sample. When information on the lichen species was available it was included (lichen species).

No	sequence ID	GenBank Accession number	GenBank species ID	photobiont source	lichen species
1	Antarctica_BrownHills_JN204750_Lecidea_cancriformis	JN204750	*Trebouxia sp.*	lichen	*Lecidea cancriformis*
2	Antarctica_CharcotIsl_AJ431576_Umbilicaria_antarctica	AJ431576	*Trebouxia jamesii*	lichen	*Umbilicaria antarctica*
3	Antarctica_DiamondHills_JN204803_Carbonea_vorticosa	JN204803	*Trebouxia sp.*	lichen	*Carbonea vorticosa*
4	Antarctica_DryValleys_JX036166_Lecidea_cancriformis	JX036166	*Trebouxia sp.*	lichen	*Lecidea cancriformis*
5	Antarctica_GQ375316_Cetraria_aculeata	GQ375316	*Trebouxia jamesii*	lichen	*Cetraria aculeata*
6	Antarctica_McMurdoDryValleys_JX036211_Sarcogyne privigna	JX036211	*Trebouxia sp.*	lichen	*Sarcogyne privigunknown*
7	Antarctica_TaylorValley_JN204734_Lecidea_sp	JN204734	*Trebouxia sp.*	lichen	*Lecidea sp*
8	Australia_DQ166611_Lecanora_rupicola	DQ166611	*Trebouxia sp.*	lichen	*Lecanora rupicola*
9	Australia_MolongloGorgeForestPark_KJ754249_Tephromela_atra	KJ754249	*Trebouxia sp.*	lichen	*Tephromela atra*
10	Austria_Carintia_KJ754218_Tephromela_atra	KJ754218	*Trebouxia simplex*	lichen	*Tephromela atra*
11	Austria_JN204747_Carbonea_vorticosa	JN204747	*Trebouxia sp.*	lichen	*Carbonea vorticosa*
12	Austria_Karnten_JN204761_Lecidella_carpathica	JN204761	*Trebouxia sp.*	lichen	*Lecidella carpathica*
13	Austria_Styria_AF242459i_Pseudevernia_furfuracea	AF242459	*Trebouxia jamesii*	lichen	*Pseudevernia furfuracea*
14	Austria_Styria_EU795082_Physconia_distorta	EU795082	*Trebouxia sp.*	lichen	*Physconia distorta*
15	Chile_GQ375363i_Cetraria_aculeata	GQ375363	*Trebouxia jamesii*	lichen	*Cetraria aculeata*
16	Trebouxia_erici_SAG 32.85_FJ626729	FJ626729	*Trebouxia erici*	axenic culture SAG 32.85	*Cladonia cristatella*
17	Asterochloris_irregularis_SAG 33.85_FJ626732	FJ626732	*Asterochloris irregularis*	axenic culture SAG 33.85	*Stereocaulon evolutoides*
18	Trebouxia_asymmetrica_SAG48.88_AJ249565	AJ249565	*Trebouxia asymmetrica*	axenic culture SAG48.88	*Diploschistes diacapsis*
19	Czech Republic_StudenecMt_AM905998_Diploschistes_muscorum	AM905998	*Asterochloris glomerata*	lichen	*Diploschistes muscorum*
20	CzechRepublic_GU339204_Protoparmeliopsis muralis	GU339204	*Trebouxia sp.*	lichen	*Protoparmeliopsis muralis*
21	Falklands_GQ375361i_Cetraria_aculeata	GQ375361	*Trebouxia jamesii*	lichen	*Cetraria aculeata*
22	Finland_AY444756_Flavocetraria_nivalis	AY444756	*Trebouxia jamesii*	lichen	*Flavocetraria nivalis*

No	sequence ID	GenBank Accession number	GenBank species ID	photobiont source	lichen species
23	Finland_KJ576660_Bryoria_implexa	KJ576660	*Trebouxia sp.*	lichen	*Bryoria implexa*
24	France_Corsica_DQ166591_Lecanora_rupicola	DQ166591	*Trebouxia sp.*	lichen	*Lecanora rupicola*
25	France_Corsica_DQ166592_Lecanora_rupicola	DQ166592	*Trebouxia sp.*	lichen	*Lecanora rupicola*
26	Germany_AJ293775_Physcia_aipolia	AJ293775	*Trebouxia sp.*	lichen	*Physcia aipolia*
27	Germany_AJ293779_Physconia_perisidiosa	AJ293779	*Trebouxia sp.*	lichen	*Physconia perisidiosa*
28	Germany_AJ293785_Dimelaena_oreina	AJ293785	*Trebouxia sp.*	lichen	*Dimelaena oreina*
29	Germany_Bavaria_KT215318_Diploschistes_muscorum	KT215318	*Trebouxia sp.*	lichen	*Diploschistes muscorum*
30	Germany_Gossenheim_KT215307_Cladonia_symphycarpa	KT215307	*Asterochloris sp.*	lichen	*Cladonia symphycarpa*
31	Germany_GQ375333_Cetraria_aculeata	GQ375333	*Trebouxia jamesii*	lichen	*Cetraria aculeata*
32	Germany_Speyer_DQ133481_Protoparmeliopsis_muralis	DQ133481	*Trebouxia sp.*	lichen	*Protoparmeliopsis muralis*
33	Greenland_AJ969505_Xanthoria_borealis	AJ969505	*Trebouxia sp.*	lichen	*Xanthoria borealis*
34	Greenland_KJ576661_Bryoria_lanestris	KJ576661	*Trebouxia sp.*	lichen	*Bryoria lanestris*
35	Iceland_AY444764_Flavocetraria_nivalis	AY444764	*Trebouxia jamesii*	lichen	*Flavocetraria nivalis*
36	Iceland_GQ375318_Cetraria_aculeata	GQ375318	*Trebouxia jamesii*	lichen	*Cetraria aculeata*
37	Iceland_GQ375323_Cetraria_aculeata	GQ375323	*Trebouxia jamesii*	lichen	*Cetraria aculeata*
38	Trebouxia_irregularis_UTEX2236_AF345411	AF345411	*Trebouxia irregularis*	axenic culture UTEX2236, Holotype	*Stereucaulon sp*
39	Iran_KJ576654_Bryoria_implexa	KJ576654	*Trebouxia sp.*	lichen	*Bryoria implexa*
40	Italy_EU551541_Tephromela_atra	EU551541	*Trebouxia sp.*	lichen	*Tephromela atra*
41	Italy_Toscana_EU795072_Physconia_grisea	EU795072	*Trebouxia sp.*	lichen	*Physconia grisea*
42	Italy_Trento_GU017647_Varicellaria_carneonivea	GU017647	*Asterochloris phycobiontica*	lichen	*Varicellaria carneonivea*
43	Italy_Trieste_DQ133501_Protoparmeliopsis_muralis	DQ133501	*Trebouxia sp.*	lichen	*Protoparmeliopsis muralis*
44	Kyrgyzstan_AM159206_Xanthoria_sp	AM159206	*Trebouxia sp.*	lichen	*Xanthoria sp*
45	Norway_AY444751_Flavocetraria_nivalis	AY444751	*Trebouxia jamesii*	lichen	*Flavocetraria nivalis*
46	Norway_KJ576699_Bryoria_subcana	KJ576699	*Trebouxia sp.*	lichen	*Bryoria subcana*
47	Norway_KJ754209_T_impressa_Tephromela_atra	KJ754209	*Trebouxia impressa*	axenic culture	*Tephromela atra*

No	sequence ID	GenBank Accession number	GenBank species ID	photobiont source	lichen species
48	Norway_Kongsvold_DQ166613_Lecanora_rupicola	DQ166613	*Trebouxia sp.*	lichen	*Lecanora rupicola*
49	Norway_Svalbard_GQ375319_Cetraria_aculeata	GQ375319	*Trebouxia jamesii*	lichen	*Cetraria aculeata*
50	Norway_Svalbard_GQ375365_Cetraria_aculeata	GQ375365	*Trebouxia jamesii*	lichen	*Cetraria aculeata*
51	Norway_Svalbard_KJ754222_Tephromela_atra	KJ754222	*Trebouxia simplex*	lichen	*Tephromela atra*
52	Norway_Urnes_DQ166614_Lecanora_rupicola	DQ166614	*Trebouxia sp.*	lichen	*Lecanora rupicola*
53	NZ_SouthIsl_KJ754231_Tephromela_atra	KJ754231	*Trebouxia simplex*	lichen	*Tephromela atra*
54	NZ_SouthIsl_KJ754234_Tephromela_atra	KJ754234	*Trebouxia simplex*	lichen	*Tephromela atra*
55	NZ_SouthIsl_KJ754235_Tephromela_atra	KJ754235	*Trebouxia simplex*	lichen	*Tephromela atra*
56	Poland_AY444752_Flavocetraria_nivalis	AY444752	*Trebouxia jamesii*	lichen	*Flavocetraria nivalis*
57	Poland_Malbork_AY703903_Protoparmeliopsis_muralis	AY703903	*Trebouxia sp.*	lichen	*Protoparmeliopsis muralis*
58	Poland_Malbork_AY804021_Protoparmeliopsis muralis	AY804021	*Trebouxia sp.*	lichen	*Protoparmeliopsis muralis*
59	Poland_Pojezierze_EU795080_Physconia_enteroxantha	EU795080	*Trebouxia sp.*	lichen	*Physconia enteroxantha*
60	Portugal_TrasOsMontes_FM205728_Cladonia_foliacea	FM205728	*Asterochloris sp.*	lichen	*Cladonia foliacea*
61	Russia_SakhaRep_KJ576665_Bryoria_simplicior	KJ576665	*Trebouxia sp.*	lichen	*Bryoria simplicior*
62	Spain_Almarza_KJ754247_Tephromela_atra	KJ754247	*Trebouxia sp.*	axenic culture	*Tephromela atra*
63	Spain_GranCanaria_DQ166582_Leconora_rupicola	DQ166582	*Trebouxia sp.*	lichen	*Leconora rupicola*
64	Spain_Madrid_DQ166595_Tephromela_atra	DQ166595	*Trebouxia sp.*	lichen	*Lecanora rupicola*
65	Spain_Madrid_DQ166597_Lecanora_rupicola	DQ166597	*Trebouxia sp.*	lichen	*Lecanora rupicola*
66	Spain_Madrid_DQ166603_Lecanora_rupicola	DQ166603	*Trebouxia sp.*	lichen	*Lecanora rupicola*
67	Spain_Soria_FM205726_Cladonia_foliacea	FM205726	*Asterochloris sp.*	lichen	*Cladonia foliacea*
68	Spain_Tarragona_FM205725_Cladonia_foliacea	FM205725	*Asterochloris sp.*	lichen	*Cladonia foliacea*
69	Spain_Tenerife_KJ576689_Bryoria_capillaris	KJ576689	*Trebouxia sp.*	lichen	*Bryoria capillaris*
70	Sweden_AY444763_Flavocetraria_nivalis	AY444763	*Trebouxia jamesii*	lichen	*Flavocetraria nivalis*
71	Sweden_HQ667313_Lecidea_polytrichina	HQ667313	*Trebouxia jamesii*	lichen	*Lecidea polytrichiunknown*
72	Sweden_Jaemtland_AF453262_Chaenotheca_subroscida	AF453262	*Trebouxia simplex*	lichen	*Chaenotheca subroscida*

No	sequence ID	GenBank Accession number	GenBank species ID	photobiont source	lichen species
73	Sweden_KJ576663_Bryoria_nadvornikiana	KJ576663	*Trebouxia sp.*	lichen	*Bryoria nadvornikiana*
74	Sweden_Oland_KT215305_Diploschistes_muscorum	KT215305	*Asterochloris sp.*	lichen	*Diploschistes muscorum*
75	Sweden_Oland_KT215311_Cladonia_symphycarpa	KT215311	*Asterochloris sp.*	lichen	*Cladonia symphycarpa*
76	Sweden_Oland_KT215315_Diploschistes_muscorum	KT215315	*Trebouxia sp.*	lichen	*Diploschistes muscorum*
77	Sweden_Uppland_KJ754213_Tephromela_atra	KJ754213	*Trebouxia simplex*	lichen	*Tephromela atra*
78	Switzerland_AM159205_Xanthoria_sp	AM159205	*Trebouxia sp.*	lichen	*Xanthoria sp*
79	Trebouxia_corticola_UTEX909_AJ249566	AJ249566	*Trebouxia corticola*	axenic culture UTEX909	unknown
80	Trebouxia_impressa_UTEX892_AF345891	AF345891	*Trebouxia impressa*	axenic culture UTEX892	*Physcia stellaris*
81	Trebouxia_jamesii_UTEX2233_FJ626733	FJ626733	*Pseudotrebouxia jamesii*	axenic culture UTEX2233, Holotype	*Schaereria tenebrosa*
82	Trebouxia_simplex_SAG101.80_FJ626735	FJ626735	*Trebouxia simplex*	axenic culture SAG 101.80	*unknown*
83	Ukraine_KaragachMt_KT819944_Diploschistes_diacapsis	KT819944	*Trebouxia sp.*	lichen	*Diploschistes diacapsis*
84	Ukraine_Kitaihorod_AY703907_Protoparmeliopsis_muralis	AY703907	*Trebouxia sp.*	lichen	*Protoparmeliopsis muralis*
85	Trebouxia_vagua_AV092_KT819943	KT819943	*Trebouxia vagua*	lichen	*Circinaria contorta*
86	Trebouxia_simplex_KT819983	KT819983	*Trebouxia simplex*	lichen	*Umbilicaria grisea*
87	USA_AJ969525_Xanthoria_fallax	AJ969525	*Trebouxia sp.*	lichen	*Xanthoria fallax*
88	USA_Arizona_DQ166577_Lecanora_bicincta	DQ166577	*Trebouxia sp.*	lichen	*Lecanora bicincta*
89	USA_HQ667308i_Schaereria_dolodes	HQ667308	*Trebouxia jamesii*	lichen	*Schaereria dolodes*
90	USA_Missouri_KF800354_uncultured_eukariote_house_dust	KF800354	*uncultured eukaryote*	environmental sample	NA
91	USA_Oregon_KJ754219_Tephromela_atra	KJ754219	*Trebouxia simplex*	lichen	*Tephromela atra*

Table S5. Summary of datasets used for phylogenetic inference. The table shows the name of the datasets, the lichen symbiont (symbiont), the markers considered for each dataset, the species used as outgroups for rooting the phylogenies (OG), the length in base pairs of the trimmed alignment (TAL), id number of GenBank sequences (#GB), the number of parsimony-informative sites (PI), and the nucleotide substitution model chosen for each of the dataset

Dataset	symbiont	markers used	OG	Total no of sequences	# GB	AL (bp)	PI	NSM
**F1**	*Thamnolia*	ITS1	n/a	249	35	166	8	SYM + I
**F2**	*Thamnolia*	ITS1+ITS2	n/a	154	5	669	30	TrNef + I
**F3**	*Thamnolia*	ITS<br>DEAD<br>EFα	n/a	129	0	1347	ITS = 28<br>DEAD = 10<br>EF=6	ITS = TrNef + G<br>DEAD = JC<br>EF=JC
**F4**	*Thamnolia*	ITS<br>IGS<br>DEAD<br>EFα<br>RPB2<br>β-tub	*D. baeomyces; I. ericetorum; S. ceratites*	54	0	1982	ITS = 44<br>IGS = 12<br>DEAD = 12<br>EF = 36<br>RPB2 = 59<br>β-tub = 28	ITS = K80 + G<br>IGS =K80<br>DEAD = TPM2 + G<br>EF = TrNef + G<br>RPB2 = TIM1ef+G<br>β-tub = TIM2ef+G
**A1**	*Trebouxia*	ITS	*T. corticola*	214	91	680	220	K80 + G
**A2**	*Trebouxia*	ITS<br>Actin<br>COX	n/a<br>n/a<br>n/a	73	0	1788	ITS = 118<br>Actin = 123<br>COX = 60	ITS = TPM1 + G<br>Actin = TPM3 + G;<br>COX = TrN

ITS = Internal transcribed spacer; IGS = Intergenic transcribed spacer; DEAD = Dead-box helicase; EFα = elongation factor alpha; RPB2 = RNA polymerase II second largest subunit; β-tub = beta tubulin; Actin = Actin gene; COX = Cytochrome c oxidase;

Table S6. GenBank accession numbers of the newly generated sequences. The table shows how many markers were sequenced with Sanger technology for each of the samples of *Thamnolia* for both mycobiont and photobiont. We obtained one copy for all mycobiont and photobiont loci, except for in two samples (Nepal_Dolpo_349 and Sweden_Taerna_219), where we obtained two different copies of the photbiont ITS. The sequences for the last four samples in the table were obtained from preliminary fungal genomes of *D. baeomyces, I. ericetorum, S. ceratites* and *Thamnolia sp*. through a blast search.

Sample ID	MYCOBIONT markers						PHOTOBIONT markers		
	ITS	DEAD	EF	RPB2	β-tub	IGS	ITS	Actin	COX
Australia_AggieMt_554.3	KY550205	n/a	n/a	n/a	n/a	n/a	n/a	n/a	n/a
Australia_AggieMt_567.1	KY550075	n/a	n/a	n/a	n/a	n/a	n/a	n/a	n/a
Australia_AggieMt_567.2	KY550076	n/a	n/a	n/a	n/a	n/a	n/a	n/a	n/a
Austria_SeetalerAlpen_427.1	KY550188	n/a	n/a	n/a	n/a	n/a	n/a	n/a	n/a
Austria_SeetalerAlpen_427.2	KY550189	n/a	n/a	n/a	n/a	n/a	n/a	n/a	n/a
Austria_SeetalerAlpen_428	KY550034	n/a	n/a	n/a	n/a	n/a	n/a	n/a	n/a
Austria_SeetalerAlpen_429	KY550190	n/a	n/a	n/a	n/a	n/a	n/a	n/a	n/a
Austria_SeetalerAlpen_430	KY550035	n/a	n/a	n/a	n/a	n/a	n/a	n/a	n/a
Austria_SeetalerAlpen_431	KY550036	n/a	n/a	n/a	n/a	n/a	n/a	n/a	n/a
Austria_SeetalerAlpen_432	KY550037	n/a	n/a	n/a	n/a	n/a	n/a	n/a	n/a
Austria_Wolkerkogel_265	KY550127	KY634070	KY634021	KY633970	KY634132	KY633915	KY559147	KY634180	KY633818
Austria_Wolkerkogel_266	KY550128	KY634071	KY634020	KY633971	KY634133	KY633916	KY559148	n/a	n/a
Bolivia_ApachetaChucura_381	KY550027	n/a	n/a	n/a	n/a	n/a	n/a	n/a	n/a
Bolivia_ApachetaChucura_382	KY550028	n/a	n/a	n/a	n/a	n/a	n/a	n/a	n/a
Bolivia_ApachetaChucura_383	KY550029	n/a	n/a	n/a	n/a	n/a	n/a	n/a	n/a
Bolivia_LaCumbre_376	KY550023	n/a	n/a	n/a	n/a	n/a	n/a	n/a	n/a
Bolivia_LaCumbre_377	KY550024	n/a	n/a	n/a	n/a	n/a	n/a	n/a	n/a
Bolivia_LaCumbre_378	KY550025	n/a	n/a	n/a	n/a	n/a	n/a	n/a	n/a
Bolivia_LaCumbre_379	KY550026	n/a	n/a	n/a	n/a	n/a	n/a	n/a	n/a
Bolivia_LaCumbre_380	KY612989	n/a	n/a	n/a	n/a	n/a	n/a	n/a	n/a
Bolivia_SamanaPampa_386	KY550030	n/a	n/a	n/a	n/a	n/a	n/a	n/a	n/a
Bolivia_SamanaPampa_387	KY550031	n/a	n/a	n/a	n/a	n/a	n/a	n/a	n/a

Sample ID	MYCOBIONT markers						PHOTOBIONT markers		
	**ITS**	**DEAD**	**EF**	**RPB2**	**β-tub**	**IGS**	**ITS**	**Actin**	**COX**
Bolivia_SamanaPampa_388	KY550032	n/a	n/a	n/a	n/a	n/a	n/a	n/a	n/a
Bolivia_SamanaPampa_389	KY550033	n/a	n/a	n/a	n/a	n/a	n/a	n/a	n/a
Bolivia_SamanaPampa_390	KY550184	n/a	n/a	n/a	n/a	n/a	n/a	n/a	n/a
Canada_Avalon_285	KY550136	KY654447	KY654355	n/a	n/a	n/a	KY559149	n/a	n/a
Canada_BritishColombia_272	KY550129	KY654441	KY654352	n/a	n/a	n/a	KY559207	KY634218	KY633869
Canada_RadioTowerMt_277	KY550130	KY654442	KY654353	n/a	n/a	n/a	KY559224	KY634197	KY633870
Canada_RadioTowerMt_280	KY550132	KY654443	KY654354	n/a	n/a	n/a	KY559225	KY634220	KY633871
Chile_Brunswick_557.3	KY550066	n/a	n/a	n/a	n/a	n/a	n/a	n/a	n/a
Chile_Navarino_516.1	KY550061	n/a	n/a	n/a	n/a	n/a	n/a	n/a	n/a
Chile_Navarino_516.2	KY550062	n/a	n/a	n/a	n/a	n/a	n/a	n/a	n/a
Chile_Navarino_516.3	KY550063	n/a	n/a	n/a	n/a	n/a	n/a	n/a	n/a
Chile_Navarino_556.3	KY550206	n/a	n/a	n/a	n/a	n/a	n/a	n/a	n/a
China_Sichuan_566.1	KY550074	n/a	n/a	n/a	n/a	n/a	n/a	n/a	n/a
China_Sichuan_566.2	KY550208	n/a	n/a	n/a	n/a	n/a	n/a	n/a	n/a
Colombia_Boyara_473	KY550046	n/a	n/a	n/a	n/a	n/a	n/a	n/a	n/a
Colombia_Boyara_474	KY550047	n/a	n/a	n/a	n/a	n/a	n/a	n/a	n/a
Colombia_Boyara_476	KY550048	n/a	n/a	n/a	n/a	n/a	n/a	n/a	n/a
Colombia_LagoPanuelo_467	KY550043	n/a	n/a	n/a	n/a	n/a	n/a	n/a	n/a
Colombia_LagoPanuelo_468	KY550044	n/a	n/a	n/a	n/a	n/a	n/a	n/a	n/a
Colombia_LagoPanuelo_469	KY550045	n/a	n/a	n/a	n/a	n/a	n/a	n/a	n/a
Falklands_KentMt_558.1	KY550067	n/a	n/a	n/a	n/a	n/a	n/a	n/a	n/a
Falklands_KentMt_558.3	KY550068	n/a	n/a	n/a	n/a	n/a	n/a	n/a	n/a
Falklands_MariaMt_559.1	KY550069	n/a	n/a	n/a	n/a	n/a	n/a	n/a	n/a
Falklands_MariaMt_559.2	KY550070	n/a	n/a	n/a	n/a	n/a	n/a	n/a	n/a
Falklands_MariaMt_559.3	KY550071	n/a	n/a	n/a	n/a	n/a	n/a	n/a	n/a

Sample ID	MYCOBIONT markers						PHOTOBIONT markers		
	ITS	DEAD	EF	RPB2	β-tub	IGS	ITS	Actin	COX
Greenland_ Qaanaaq_263	KY550126	KY654440	KY654351	n/a	n/a	n/a	KY559146	KY634179	KY633817
Greenland_CapeMorrisJesup_262	KY612987	n/a	n/a	n/a	n/a	n/a	KY559110	n/a	n/a
Greenland_Ilulissat_252	KY550123	KY654438	KY634056	n/a	n/a	n/a	KY559144	KY634178	KY633815
Greenland_Naarssarsuaq_260	KY550125	KY654439	KY634042	n/a	n/a	n/a	KY559145	n/a	n/a
Greenland_Siorapaluk_300	KY550146	KY654455	KY654359	n/a	n/a	n/a	KY559156	KY634182	KY633828
Iceland_Naustavik_104	KY550083	KY654407	KY634056	n/a	n/a	n/a	KY559128	KY634195	KY633797
Iceland_Skaftafellssysla_103	KY612986	n/a	n/a	n/a	n/a	n/a	KY559127	n/a	n/a
Iceland_Skagafjardarsysla_84	KY550219	KY634078	KY634055	KY633990	KY634159	KY633949	KY559170	KY634192	KY633843
Iceland_Skagafjardarsysla_85	KY550220	KY634079	KY634056	KY633985	KY634160	KY633950	KY559171	KY634193	KY633844
Iceland_Skagafjardarsysla_87	KY550221	KY634080	KY634057	KY633986	KY634161	KY633951	KY559172	KY634194	KY633841
Iceland_Vadalfjol_105	KY550084	KY654408	KY634056	n/a	n/a	n/a	KY559129	n/a	n/a
Japan_282.B	KY550133	KY654444	KY654398	n/a	n/a	n/a	n/a	n/a	n/a
Japan_Yamanashi_246.3	KY550122	KY654437	KY654397	n/a	n/a	n/a	KY559143	KY634177	KY633814
Komi_Ural_329	KY550162	KY654469	KY654376	n/a	n/a	n/a	KY559196	KY634198	KY633883
Nepal_Dolpo_349	KY550172	KY634097	KY654381	n/a	n/a	n/a	KY559118	n/a	n/a
Nepal_Dolpo_349	n/a	n/a	n/a	n/a	n/a	n/a	KY559234	n/a	n/a
Nepal_Dolpo_352	KY550174	KY634097	KY634041	KY633992	KY634143	KY633923	KY559119	n/a	n/a
Nepal_Humla_355.1	KY550175	KY654477	KY654404	n/a	n/a	n/a	KY559120	n/a	n/a
Nepal_Humla_356	KY550176	KY654478	KY654382	n/a	n/a	n/a	KY559121	n/a	n/a
Nepal_Khumbu_344	KY550170	KY654475	KY654379	n/a	n/a	n/a	KY559116	n/a	n/a
Nepal_Khumbu_345	KY550171	KY654476	KY654380	n/a	n/a	n/a	KY559117	n/a	n/a
Nepal_Thare_363	KY550177	KY654479	KY654383	n/a	n/a	n/a	KY559122	n/a	n/a
Nepal_Thare_366	KY550178	KY654480	KY654384	n/a	n/a	n/a	KY559123	n/a	n/a
Norway_Finmark_116	KY550088	KY654411	KY634024	n/a	n/a	n/a	KY559209	KY634207	KY633850
Norway_Finmark_117	KY550089	KY634066	KY634017	KY633966	KY634123	KY633922	KY559212	n/a	n/a

Sample ID	MYCOBIONT markers						PHOTOBIONT markers		
	**ITS**	**DEAD**	**EF**	**RPB2**	**β-tub**	**IGS**	**ITS**	**Actin**	**COX**
Norway_Finmark_118	KY550090	KY654412	KY634024	n/a	n/a	n/a	KY559210	n/a	n/a
Norway_Finmark_119.1	KY550091	KY654413	KY654385	n/a	n/a	n/a	KY559211	n/a	n/a
Norway_Finmark_122	KY550092	KY654414	KY634056	n/a	n/a	n/a	KY559132	KY634169	KY633802
Norway_Finmark_124	KY550093	KY654415	KY634024	n/a	n/a	n/a	KY559231	KY634200	KY633854
Norway_Hedmark_218	KY550113	KY654428	KY634050	n/a	n/a	n/a	KY559139	KY634174	KY633809
Norway_Hordaland_295	KY550144	KY654453	KY654358	n/a	n/a	n/a	KY559173	n/a	n/a
Norway_Juvasshytta_06	KY550078	KY634084	KY634024	KY633987	KY634117	KY633952	KY559126	KY634167	KY633794
Norway_SognOgFjordane_296	KY550145	KY654454	KY654360	n/a	n/a	n/a	KY559155	KY634181	KY633827
Norway_Svalbard_01	KY497256	KY634073	KY634050	KY633973	KY634112	KY633953	KY559124	KY634165	KY633792
Norway_Svalbard_319	KY550154	KY654462	KY654367	n/a	n/a	n/a	KY559214	n/a	n/a
Norway_Svalbard_54	KY550204	KY634077	KY634051	KY633974	KY634149	KY633954	KY559166	KY634188	KY633837
Norway_Svalbard_56	KY550207	KY634074	KY634052	KY633975	KY634150	KY633955	KY559167	KY634189	KY633837
Norway_Svalbard_61	KY550209	KY634075	KY634054	KY633976	KY634151	KY633956	KY559168	KY634190	KY633838
Norway_Svalbard_62.3	KY550210	KY654481	KY654405	n/a	n/a	n/a	KY559219	KY634225	KY633889
NZ_Cantebury_564.1	KY550072	n/a	n/a	n/a	n/a	n/a	n/a	n/a	n/a
NZ_Cantebury_565.1	KY550073	n/a	n/a	n/a	n/a	n/a	n/a	n/a	n/a
Peru_AlbodePucaraju_492	KY550057	n/a	n/a	n/a	n/a	n/a	n/a	n/a	n/a
Peru_AlbodePucaraju_495	KY550058	n/a	n/a	n/a	n/a	n/a	n/a	n/a	n/a
Peru_AlbodePucaraju_496	KY550195	n/a	n/a	n/a	n/a	n/a	n/a	n/a	n/a
Peru_Ancash_395.2	KY550187	n/a	n/a	n/a	n/a	n/a	n/a	n/a	n/a
Peru_Bolognesi_393	KY550185	n/a	n/a	n/a	n/a	n/a	n/a	n/a	n/a
Peru_CordilleraBlanca_478	KY550049	n/a	n/a	n/a	n/a	n/a	n/a	n/a	n/a
Peru_CordilleraBlanca_479	KY550050	n/a	n/a	n/a	n/a	n/a	n/a	n/a	n/a
Peru_CordilleraBlanca_481	KY550051	n/a	n/a	n/a	n/a	n/a	n/a	n/a	n/a
Peru_Huaraz_394.2	KY550186	n/a	n/a	n/a	n/a	n/a	n/a	n/a	n/a

Sample ID	MYCOBIONT markers						PHOTOBIONT markers		
	ITS	DEAD	EF	RPB2	β-tub	IGS	ITS	Actin	COX
Peru_Lagun/a69_369	KY550179	n/a	n/a	n/a	n/a	n/a	n/a	n/a	n/a
Peru_Lagun/a69_370	KY550181	n/a	n/a	n/a	n/a	n/a	n/a	n/a	n/a
Peru_Lagun/a69_371	KY550020	n/a	n/a	n/a	n/a	n/a	n/a	n/a	n/a
Peru_Lagun/a69_372	KY550021	n/a	n/a	n/a	n/a	n/a	n/a	n/a	n/a
Peru_Lagun/a69_373	KY550022	n/a	n/a	n/a	n/a	n/a	n/a	n/a	n/a
Peru_Lagun/a69_464	KY550040	n/a	n/a	n/a	n/a	n/a	n/a	n/a	n/a
Peru_Lagun/a69_465	KY550041	n/a	n/a	n/a	n/a	n/a	n/a	n/a	n/a
Peru_Lagun/a69_466	KY550042	n/a	n/a	n/a	n/a	n/a	n/a	n/a	n/a
Peru_LagunaSafunaBaja_482	KY550052	n/a	n/a	n/a	n/a	n/a	n/a	n/a	n/a
Peru_LagunaSafunaBaja_484	KY550053	n/a	n/a	n/a	n/a	n/a	n/a	n/a	n/a
Peru_LagunaSafunaBaja_485	KY550054	n/a	n/a	n/a	n/a	n/a	n/a	n/a	n/a
Peru_Langoni_497	KY550059	n/a	n/a	n/a	n/a	n/a	n/a	n/a	n/a
Peru_Langoni_500	KY550196	n/a	n/a	n/a	n/a	n/a	n/a	n/a	n/a
Peru_Langoni_501	KY550060	n/a	n/a	n/a	n/a	n/a	n/a	n/a	n/a
Peru_QuebradaMatara_502	KY550197	n/a	n/a	n/a	n/a	n/a	n/a	n/a	n/a
Peru_QuebradaMatara_504	KY550198	n/a	n/a	n/a	n/a	n/a	n/a	n/a	n/a
Peru_QuebradaMatara_505	KY550199	n/a	n/a	n/a	n/a	n/a	n/a	n/a	n/a
Peru_Tarma_392.2	KY612985	n/a	n/a	n/a	n/a	n/a	n/a	n/a	n/a
Peru_Tupatupa_507	KY550200	n/a	n/a	n/a	n/a	n/a	n/a	n/a	n/a
Peru_Tupatupa_508	KY550201	n/a	n/a	n/a	n/a	n/a	n/a	n/a	n/a
Peru_Tupatupa_511	KY550202	n/a	n/a	n/a	n/a	n/a	n/a	n/a	n/a
Peru_Yanapaccha_489	KY550055	n/a	n/a	n/a	n/a	n/a	n/a	n/a	n/a
Peru_Yanapaccha_491	KY550056	n/a	n/a	n/a	n/a	n/a	n/a	n/a	n/a
Poland_TatraMt_301	KY550147	KY654456	KY654361	n/a	n/a	n/a	KY559157	n/a	n/a
Poland_TatraMt_303	KY550148	KY654457	KY654362	n/a	n/a	n/a	KY559158	n/a	n/a

Sample ID	MYCOBIONT markers						PHOTOBIONT markers		
	ITS	DEAD	EF	RPB2	β-tub	IGS	ITS	Actin	COX
Poland_TatraMt_304	KY612990	KY654458	KY654363	n/a	n/a	n/a	KY559159	n/a	n/a
Poland_TatraMt_305	KY612988	n/a	n/a	n/a	n/a	n/a	KY559174	n/a	n/a
Poland_TatraMt_306	KY550149	KY654459	KY654364	n/a	n/a	n/a	KY559160	n/a	n/a
Romania_BucegiMt_02	KY497257	KY634095	KY634047	KY633979	KY634113	KY633930	KY559175	KY634164	KY633788
Romania_BucegiMt_239.1	KY550120	KY654435	KY654395	n/a	n/a	n/a	KY559176	n/a	n/a
Romania_BucegiMt_239.2	KY550121	KY654436	KY654396	n/a	n/a	n/a	KY559179	n/a	n/a
Romania_BucegiMt_45	KY550193	KY634096	KY634048	KY633991	KY634147	KY633931	KY559177	KY634162	KY633787
Romania_BucegiMt_53	KY550203	KY634098	KY634049	KY633994	KY634148	KY633932	KY559178	KY634163	KY633790
Romania_BucegiMt_97	KY550222	KY634096	KY634047	n/a	n/a	n/a	KY559180	n/a	n/a
Romania_RetezatMt_435	KY550038	n/a	n/a	n/a	n/a	n/a	n/a	n/a	n/a
Romania_RetezatMt_449	KY550039	n/a	n/a	n/a	n/a	n/a	n/a	n/a	n/a
Romania_RetezatMt_463	KY550194	n/a	n/a	n/a	n/a	n/a	n/a	n/a	n/a
Russia_ LitovkaMt_311	KY550151	KY654461	KY654366	n/a	n/a	n/a	KY559229	n/a	n/a
Russia_ MalayaKhatiparaMt_327	KY550160	KY654467	KY654372	n/a	n/a	n/a	KY559112	n/a	n/a
Russia_ MalayaKhatiparaMt_328	KY550161	KY654468	KY654373	n/a	n/a	n/a	KY559113	n/a	n/a
Russia_AltaiMt_338	KY550168	KY654473	KY654378	n/a	n/a	n/a	KY559114	n/a	n/a
Russia_AltaiMt_339	KY550169	KY654474	KY654377	n/a	n/a	n/a	KY559115	n/a	n/a
Russia_ArbatMt_342	KY612984	n/a	n/a	n/a	n/a	n/a	KY559198	n/a	n/a
Russia_Dzuletta_310	KY550150	KY654460	KY654365	n/a	n/a	n/a	KY559230	n/a	n/a
Russia_FranzJosef_320	KY550155	KY654463	KY654368	n/a	n/a	n/a	KY559215	KY634222	KY633879
Russia_FranzJosef_321	KY550156	KY654464	KY654369	n/a	n/a	n/a	KY559216	KY634199	KY633880
Russia_FranzJosef_323	KY550157	KY634065	KY634016	KY633965	KY634139	KY633921	KY559217	n/a	n/a
Russia_FranzJosef_324	KY550158	KY654465	KY654370	n/a	n/a	n/a	KY559218	n/a	n/a
Russia_Gydan_331	KY550163	KY634062	KY634012	KY633964	KY634140	KY633918	KY559228	n/a	n/a
Russia_Gydan_332	KY550164	KY634067	KY634013	KY633963	KY634141	KY633912	KY559237	n/a	n/a

Sample ID	MYCOBIONT markers						PHOTOBIONT markers		
	ITS	DEAD	EF	RPB2	β-tub	IGS	ITS	Actin	COX
Russia_Kolguev_315	KY550152	KY634064	KY634018	KY633968	KY634137	KY633913	KY559232	n/a	n/a
Russia_Kolguev_316	KY550153	KY634069	KY634014	KY633962	KY634138	KY633914	KY559233	n/a	n/a
Russia_SayanMt_326	KY550159	KY654466	KY654371	n/a	n/a	n/a	KY559111	n/a	n/a
Russia_Ural_284.1	KY550134	KY654445	KY654399	n/a	n/a	n/a	KY559226	n/a	n/a
Russia_Ural_284.2	KY550135	KY654446	KY654400	n/a	n/a	n/a	KY559194	n/a	n/a
Russia_Yamal_333	KY550165	KY654470	KY654374	n/a	n/a	n/a	KY559161	KY634183	KY633833
Russia_Yamal_334	KY550166	KY654471	KY654375	n/a	n/a	n/a	KY559197	n/a	n/a
Russia_Yamal_335	KY550167	KY654472	KY654376	n/a	n/a	n/a	KY559227	KY634223	KY633887
Sweden_Abisko_226	KY550116	KY654431	KY634056	n/a	n/a	n/a	KY559141	n/a	n/a
Sweden_Abisko_229	KY550117	KY654432	KY634040	n/a	n/a	n/a	KY559220	KY634196	KY633867
Sweden_Abisko_234	KY550118	KY654433	KY634040	n/a	n/a	n/a	KY559221	KY634216	KY633868
Sweden_Abisko_235	KY550119	KY654434	KY634056	n/a	n/a	n/a	KY559142	n/a	n/a
Sweden_Gaelivare_72	KY550215	KY634076	KY634053	KY633977	KY634156	KY633957	KY559169	KY634191	KY633839
Sweden_Gotland_04	KY497259	KY634101	KY634042	KY633980	KY634115	KY633942	KY559182	KY634202	KY633846
Sweden_Gotland_63	KY550211	KY634090	KY634044	KY633978	KY634152	KY633943	KY559200	KY634226	KY633890
Sweden_Gotland_64	KY550212	KY634092	KY634040	KY633981	KY634153	KY633944	KY559201	KY634227	KY633891
Sweden_Gotland_65	KY550213	KY634091	KY634046	KY633982	KY634154	KY633945	KY559202	KY634228	KY633892
Sweden_Gotland_71	KY550214	KY634109	KY634036	KY633983	KY634155	KY633946	KY559203	KY634229	KY633893
Sweden_Jaemtlands_214.1	KY550107	KY654423	KY654388	n/a	n/a	n/a	KY559137	KY634173	KY633807
Sweden_Jaemtlands_214.2	KY550108	KY654424	KY654389	n/a	n/a	n/a	KY559205	n/a	n/a
Sweden_Jaemtlands_216.1	KY550109	KY654425	KY654390	n/a	n/a	n/a	KY559193	KY634201	KY633864
Sweden_Jaemtlands_216.2	KY550110	KY634063	KY634015	n/a	KY634130	KY633920	KY559138	n/a	n/a
Sweden_Jaemtlands_217.1	KY550111	KY654426	KY654391	n/a	n/a	n/a	KY559213	KY634214	KY633865
Sweden_Jaemtlands_217.2	KY550112	KY654427	KY654392	n/a	n/a	n/a	KY559235	KY634215	KY633866
Sweden_MoiRana_05	KY550077	KY634088	KY634059	KY633993	KY634116	KY633958	KY559125	KY634166	KY633793

Sample ID	MYCOBIONT markers						PHOTOBIONT markers		
	ITS	DEAD	EF	RPB2	β-tub	IGS	ITS	Actin	COX
Sweden_MoiRana_37	KY550180	KY634089	KY634060	KY633989	KY634144	KY633959	KY559162	KY634184	KY633842
Sweden_MoiRana_38	KY550182	KY634089	KY634059	n/a	n/a	n/a	KY559163	KY634185	KY633834
Sweden_MoiRana_39	KY550183	KY634085	KY634061	KY633995	KY634145	KY633960	KY559164	KY634186	KY633835
Sweden_MoiRana_44	KY550192	KY634082	KY634058	KY633996	KY634146	KY633961	KY559165	KY634187	KY633836
Sweden_Öland_03	KY497258	KY634103	KY634027	KY634003	KY634114	KY633933	KY559181	KY634234	KY633845
Sweden_Öland_07	KY550079	KY634083	KY634025	KY633997	KY634118	KY633924	KY559183	KY634203	KY633897
Sweden_Öland_08	KY550080	KY634099	KY634045	KY633998	KY634119	KY633928	KY559184	KY634204	KY633847
Sweden_Öland_09	KY550081	KY634081	KY634038	KY634010	KY634120	KY633925	KY559185	KY634205	KY633848
Sweden_Öland_10	KY550082	KY634086	KY634043	KY634007	KY634121	KY633926	KY559186	KY634206	KY633849
Sweden_Öland_16	KY550094	KY634102	KY634026	KY633999	KY634124	KY633929	KY559187	KY634208	KY633857
Sweden_Öland_164	KY550095	KY654416	KY634024	n/a	n/a	n/a	KY559222	KY634209	KY633858
Sweden_Öland_17	KY550096	KY634110	KY634028	KY634002	KY634125	KY633934	KY559188	KY634210	KY633898
Sweden_Öland_18	KY550097	KY634104	KY634029	KY634004	KY634126	KY633927	KY559189	KY634211	KY633899
Sweden_Öland_189	KY550098	KY634108	KY634037	KY634006	KY634127	KY633941	KY559223	KY634212	KY633859
Sweden_Öland_19	KY550099	KY634100	KY634030	KY634009	KY634128	KY633935	KY559190	KY634235	KY633860
Sweden_Öland_20	KY550100	KY634105	KY634031	KY634001	KY634129	KY633936	KY559191	KY634213	KY633861
Sweden_Öland_26	KY550124	KY634087	KY634032	KY634005	KY634131	KY633937	KY559206	KY634217	KY633896
Sweden_Öland_28	KY550131	KY634111	KY634033	KY634008	KY634134	KY633938	KY559208	KY634219	KY633900
Sweden_Öland_29	KY550141	KY634106	KY634034	KY634011	KY634135	KY633939	KY559195	KY634221	KY633874
Sweden_Öland_35	KY550173	KY634107	KY634035	KY634000	KY634142	KY633940	KY559199	KY634224	KY633888
Sweden_Taerna_219.1	KY550114	KY654429	KY654393	n/a	n/a	n/a	KY559140	KY634175	KY633810
Sweden_Taerna_219.2	KY550115	KY654430	KY654394	n/a	n/a	n/a	KY559240	KY634176	KY633811
Sweden_Taerna_219.2	n/a	n/a	n/a	n/a	n/a	n/a	KY559109	n/a	n/a
Switzerland_SchynigePlatteau_433	KY550191	n/a	n/a	n/a	n/a	n/a	n/a	n/a	n/a
Switzerland_Valais_111	KY550085	KY654409	KY634022	n/a	n/a	n/a	KY559130	n/a	n/a

Sample ID	MYCOBIONT markers						PHOTOBIONT markers		
	ITS	DEAD	EF	RPB2	β-tub	IGS	ITS	Actin	COX
Switzerland_Valais_113	KY550086	KY654410	KY634026	n/a	n/a	n/a	KY559107	n/a	n/a
Switzerland_Valais_115	KY550087	KY634072	KY634022	KY633972	KY634122	KY633917	KY559131	KY634168	KY633801
USA_Aleutian_286	KY550137	KY654448	KY654356	n/a	n/a	n/a	KY559150	n/a	n/a
USA_Aleutian_287.1	KY550138	KY654449	KY654401	n/a	n/a	n/a	KY559151	n/a	n/a
USA_Aleutian_287.2	KY550139	KY654450	KY654402	n/a	n/a	n/a	KY559152	n/a	n/a
USA_Aleutian_288	KY550140	KY654451	KY654357	n/a	n/a	n/a	KY559239	n/a	n/a
USA_Aleutian_291.1	KY550142	KY654452	KY654403	n/a	n/a	n/a	KY559153	n/a	n/a
USA_Aleutian_291.2	KY550143	KY634068	KY634019	KY633967	KY634136	KY633919	KY559154	n/a	n/a
USA_FingerMt_82	KY550216	KY634093	KY634023	KY633984	KY634157	KY633947	KY559236	KY634230	KY633902
USA_Kurupa_209.2	KY550102	KY654418	KY634040	n/a	n/a	n/a	KY559108	n/a	n/a
USA_Selby_210.1	KY550103	KY654419	KY654386	n/a	n/a	n/a	KY559192	KY634233	KY633862
USA_Selby_210.2	KY550104	KY654420	KY654387	n/a	n/a	n/a	KY559134	n/a	n/a
USA_Summit_213	KY550106	KY654422	KY634040	n/a	n/a	n/a	KY559136	KY634172	KY633806
USA_Takahula_212	KY550105	KY654421	KY634047	n/a	n/a	n/a	KY559135	KY634171	KY633805
USA_Walker_205	KY550101	KY654417	KY634031	n/a	n/a	n/a	KY559133	KY634170	KY633803
USA_Wiseman-Antigu_83.1	KY550217	KY634094	KY634039	KY633988	KY634158	KY633948	KY559204	KY634231	KY633894
USA_Wiseman-Antigu_83.2	KY550218	KY654482	KY654406	n/a	n/a	n/a	KY559238	n/a	n/a
T. vermicularis	*	*	*	*	*	*	n/a	n/a	n/a
D. baeomyces	*	*	*	*	*	*	n/a	n/a	n/a
I. ericetorum	*	*	*	*	*	*	n/a	n/a	n/a
S. ceratites	*	*	*	*	*	*	n/a	n/a	n/a

ITS = Intern/al transcribed spacer; IGS = Intergenic transcribed spacer; DEAD = Dead-box helicase; EFα = elongation factor alpha; RPB2 = Rn/a polymerase II second largest subunit; β-tub = beta tubulin; Actin = Actin gene; COX = Cytochrome c oxidase; n/a = not aplicable

*the sequences are deposited as fasta files in the Dryad repository: http://dx.doi.org/10.5061/dryad.79d91

Table S7. Mycobiont haplotype numbers for each TCS haplotype network and the associated photobiont lineage. The table summarize which *Thamnolia* sequences (Sample ID) used to obtain the three haplotype networks, the mycobiont haplotypes numbers and lineages, and the lineage of the associated photobiont. For each sample the podetium morphotype and chemotype [UV+ (baeomycesic and squamatic acids), UV- (thamnolic acid)] is stated.

Sample ID	Morphology	Chemistry	Lineage	Dataset F1	Dataset F2	Dataset F3	Dataset A1
			mycobiont				photobiont
Australia_AggieMt_554.3	cylindrical and hollow	UV-	C	H1	H1c	na	na
Australia_AggieMt_567.1	cylindrical and hollow	UV-	C	H2	n/a	n/a	n/a
Australia_AggieMt_567.2	flat and wide	UV-	C	H2	n/a	n/a	n/a
Australia_NewSouthWales_AY961602	cylindrical and hollow	UV-	C	H2	n/a	n/a	n/a
Australia_NewSouthWales_AY961603	cylindrical and hollow	nd	C	H2	n/a	n/a	n/a
Austria_SeetalerAlpen_427.1	cylindrical and hollow	UV-	B	H8	H8a	n/a	n/a
Austria_SeetalerAlpen_427.2	cylindrical and hollow	UV-	B	H8	H8a	n/a	n/a
Austria_SeetalerAlpen_428	cylindrical and hollow	UV-	B	H8	n/a	n/a	n/a
Austria_SeetalerAlpen_429	cylindrical and hollow	UV-	B	H8	H8a	n/a	n/a
Austria_SeetalerAlpen_430	cylindrical and hollow	UV+	C	H1	n/a	n/a	n/a
Austria_SeetalerAlpen_431	cylindrical and hollow	UV+	C	H1	n/a	n/a	n/a
Austria_SeetalerAlpen_432	cylindrical and hollow	UV+	C	H1	n/a	n/a	n/a
Austria_Wolkerkogel_265	cylindrical and hollow	UV-	B	H8	H8b	H8b1	simplex 1
Austria_Wolkerkogel_266	cylindrical and hollow	UV-	B	H8	H8b	H8b1	simplex 1
Bolivia_ApachetaChucura_381	cylindrical and hollow	UV-	C	H1	n/a	n/a	n/a
Bolivia_ApachetaChucura_382	cylindrical and hollow	UV-	C	H1	n/a	n/a	n/a
Bolivia_ApachetaChucura_383	cylindrical and hollow	UV-	C	H1	n/a	n/a	n/a
Bolivia_LaCumbre_376	cylindrical and hollow	UV-	C	H1	n/a	n/a	n/a
Bolivia_LaCumbre_377	cylindrical and hollow	UV-	C	H1	n/a	n/a	n/a
Bolivia_LaCumbre_378	cylindrical and hollow	UV-	C	H1	n/a	n/a	n/a
Bolivia_LaCumbre_379	cylindrical and hollow	UV-	C	H1	n/a	n/a	n/a
Bolivia_LaCumbre_380	cylindrical and hollow	UV-	C	H1	n/a	n/a	n/a
Bolivia_SamanaPampa_386	cylindrical and hollow	UV-	C	H1	n/a	n/a	n/a
Bolivia_SamanaPampa_387	cylindrical and hollow	UV-	C	H1	n/a	n/a	n/a
Bolivia_SamanaPampa_388	cylindrical and hollow	UV-	C	H1	n/a	n/a	n/a
Bolivia_SamanaPampa_389	cylindrical and hollow	UV-	C	H1	n/a	n/a	n/a
Bolivia_SamanaPampa_390	cylindrical and hollow	UV-	C	H1	H1c	n/a	n/a
Canada_Avalon_285	cylindrical and hollow	UV+	C	H1	H1l	H1l1	simplex 1
Canada_PlateauMt_JQ409348	cylindrical and hollow	UV+	C	H1	n/a	n/a	n/a
Canada_RaeMt_JQ409346	cylindrical and hollow	UV+	C	H1	H1i	n/a	n/a
Chile_Brunswick_557.1	cylindrical and hollow	UV+	C	H1	n/a	n/a	n/a
Chile_Brunswick_557.2	cylindrical and hollow	UV+	C	H1	n/a	n/a	n/a
Chile_Brunswick_557.3	cylindrical and hollow	UV+	C	H1	n/a	n/a	n/a

Sample ID	Morphology	Chemistry	Lineage	Dataset F1	Dataset F2	Dataset F3	Dataset A1
				mycobiont			photobiont
Chile_Navarino_516.1	**cylindrical and hollow**	**UV-**	C	H1	n/a	n/a	n/a
Chile_Navarino_516.2	**cylindrical and hollow**	**UV-**	C	H1	n/a	n/a	n/a
Chile_Navarino_516.3	**cylindrical and hollow**	**UV-**	C	H1	n/a	n/a	n/a
Chile_Navarino_556.3	**cylindrical and hollow**	**UV-**	C	H1	H1i	n/a	n/a
China_Sichuan_566.1	**cylindrical and hollow**	**UV-**	C	H1	n/a	n/a	n/a
China_Sichuan_566.2	**cylindrical and hollow**	**UV-**	C	H1	n/a	n/a	n/a
China_Yunnan_EU714432	**cylindrical and hollow**	**UV+**	C	H1	n/a	n/a	n/a
China_Yunnan_EU714433	**cylindrical and hollow**	**UV+**	C	H1	n/a	n/a	n/a
China_Yunnan_EU714436	**cylindrical and hollow**	**UV-**	C	H1	n/a	n/a	n/a
China_Yunnan_EU714437	**cylindrical and hollow**	**UV-**	C	H1	n/a	n/a	n/a
Colombia_Boyara_473	**cylindrical and hollow**	**UV-**	C	H1	n/a	n/a	n/a
Colombia_Boyara_474	**cylindrical and hollow**	**UV-**	C	H1	n/a	n/a	n/a
Colombia_Boyara_476	**cylindrical and hollow**	**UV-**	C	H1	n/a	n/a	n/a
Colombia_LagoPanuelo_467	**cylindrical and hollow**	**UV-**	C	H1	n/a	n/a	n/a
Colombia_LagoPanuelo_468	**cylindrical and hollow**	**UV-**	C	H1	n/a	n/a	n/a
Colombia_LagoPanuelo_469	**cylindrical and hollow**	**UV-**	C	H1	n/a	n/a	n/a
CostaRica_SanJose_EU714434	**cylindrical and hollow**	**UV+**	C	H1	n/a	n/a	n/a
CostaRica_SanJose_EU714435	**cylindrical and hollow**	**UV-**	C	H1	n/a	n/a	n/a
Falklands_KentMt_558.1	**cylindrical and hollow**	**UV-**	C	H1	n/a	n/a	n/a
Falklands_KentMt_558.3	**cylindrical and hollow**	**UV-**	C	H1	n/a	n/a	n/a
Falklands_MariaMt_559.1	**cylindrical and hollow**	**UV-**	C	H1	n/a	n/a	n/a
Falklands_MariaMt_559.2	**cylindrical and hollow**	**UV-**	C	H1	n/a	n/a	n/a
Falklands_MariaMt_559.3	**cylindrical and hollow**	**UV-**	C	H1	n/a	n/a	n/a
Greenland_CapeMorrisJesup_262	**cylindrical and hollow**	**UV+**	C	H5	n/a	n/a	impressa
Greenland_Ilulissat_252	**cylindrical and hollow**	**UV+**	C	H1	H1a	H1a2	simplex 1
Greenland_Narssarsuaq_260	**cylindrical and hollow**	**UV+**	C	H1	H1a	H1a5	simplex 1
Greenland_ Qaanaaq_263	**cylindrical and hollow**	**UV+**	C	H1	H1a	H1a4	simplex 1
Greenland_Siorapaluk_300	**cylindrical and hollow**	**UV+**	C	H1	H1b	H1b1	simplex 1
Iceland_Naustavik_104	**cylindrical and hollow**	**UV+**	C	H1	H1a	H1a2	simplex 1
Iceland_Skaftafellssysla_103	**cylindrical and hollow**	**UV+**	C	H5	n/a	n/a	simplex 1
Iceland_Skagafjardarsysla_84	**cylindrical and hollow**	**UV+**	C	H1	H1a	H1a2	simplex 1
Iceland_Skagafjardarsysla_85	**cylindrical and hollow**	**UV+**	C	H1	H1a	H1a2	simplex 1
Iceland_Skagafjardarsysla_87	**cylindrical and hollow**	**UV+**	C	H1	H1a	H1a2	simplex 1
Iceland_Vadalfjol_105	**cylindrical and hollow**	**UV+**	C	H1	H1a	H1a2	simplex 1
Japan_282.B	**cylindrical and hollow**	**UV-**	C	H1	H1p	H1p	n/a
Japan_Yamanashi_246.3	**cylindrical and hollow**	**UV-**	C	H1	H1l	H1l1	simplex 1

Sample ID	Morphology	Chemistry	Lineage	Dataset F1	Dataset F2	Dataset F3	Dataset A1
			mycobiont				photobiont
Komi_Ural_329	**cylindrical and hollow**	**UV+**	C	H1	H1b	H1b1	simplex 2
Nepal_Dolpo_349	**cylindrical and hollow**	**UV+**	C	H1	H1b	H1b2	impressa simplex 2
Nepal_Dolpo_352	**cylindrical and hollow**	**UV+**	C	H1	H1m	H1m	impressa
Nepal_Humla_355.1	**cylindrical and hollow**	**UV+**	C	H1	H1n	H1n	impressa
Nepal_Humla_356	**cylindrical and hollow**	**UV+**	C	H1	H1b	H1b2	impressa
Nepal_Khumbu_344	**cylindrical and hollow**	**UV+**	C	H3	H3	H3	impressa
Nepal_Khumbu_345	**cylindrical and hollow**	**UV-**	C	H1	H1o	H1o	impressa
Nepal_Thare_363	**cylindrical and hollow**	**UV-**	C	H1	H1o	H1o	impressa
Nepal_Thare_366	**cylindrical and hollow**	**UV-**	C	H1	H1b	H1b2	impressa
Norway_Finmark_116	**cylindrical and hollow**	**UV-**	C	H1	H1d	H1d1	simplex 2
Norway_Finmark_117	**cylindrical and hollow**	**UV+**	A	H9	H9b	H9b	simplex 2
Norway_Finmark_118	**cylindrical and hollow**	**UV-**	C	H1	H1d	H1d1	simplex 2
Norway_Finmark_119.1	**cylindrical and hollow**	**UV-**	C	H1	H1d	H1d1	simplex 2
Norway_Finmark_122	**cylindrical and hollow**	**UV+**	C	H1	H1a	H1a2	simplex 1
Norway_Finmark_124	**cylindrical and hollow**	**UV+**	C	H1	H1s	H1s	simplex 2
Norway_Hedmark_218	**cylindrical and hollow**	**UV+**	C	H1	H1q	H1q1	simplex 1
Norway_Hordaland_295	**cylindrical and hollow**	**UV+**	C	H1	H1a	H1a2	simplex 1
Norway_Juvasshytta_06	**cylindrical and hollow**	**UV+**	C	H1	H1a	H1a1	simplex 1
Norway_SognOgFjordane_296	**cylindrical and hollow**	**UV+**	C	H1	H1a	H1a2	simplex 1
Norway_Svalbard_01	**cylindrical and hollow**	**UV+**	C	H1	H1q	H1q1	simplex 1
Norway_Svalbard_54	**cylindrical and hollow**	**UV+**	C	H1	H1q	H1q1	simplex 1
Norway_Svalbard_56	**cylindrical and hollow**	**UV+**	C	H1	H1q	H1q1	simplex 1
Norway_Svalbard_61	**cylindrical and hollow**	**UV+**	C	H1	H1q	H1q1	simplex 1
Norway_Svalbard_62.3	**cylindrical and hollow**	**UV+**	C	H1	H1t	H1t1	simplex 2
Norway_Svalbard_319	**cylindrical and hollow**	**UV+**	C	H1	H1t	H1t2	simplex 2
Norway_Troms_EU714430	**cylindrical and hollow**	**UV+**	C	H1	n/a	n/a	n/a
Norway_Troms_EU714431	**cylindrical and hollow**	**UV+**	C	H1	n/a	n/a	n/a
NZ_AY961604	**cylindrical and hollow**	**UV+**	C	H1	n/a	n/a	n/a
NZ_AY961605	**cylindrical and hollow**	**UV+**	C	H1	n/a	n/a	n/a
NZ_Cantebury_564.1	**cylindrical and hollow**	**UV-**	C	H1	n/a	n/a	n/a
NZ_Cantebury_565.1	**cylindrical and hollow**	**UV-**	C	H1	n/a	n/a	n/a
Peru_AlbodePucaraju_492	**flat and wide**	**UV+**	C	H1	n/a	n/a	n/a
Peru_AlbodePucaraju_495	**cylindrical and hollow**	**UV-**	C	H1	n/a	n/a	n/a
Peru_AlbodePucaraju_496	**cylindrical and hollow**	**UV-**	C	H1	H1c	n/a	n/a
Peru_Ancash_395.2	**flat and wide**	**UV-**	C	H1	H1c	n/a	n/a
Peru_Bolognesi_393	**flat and wide**	**UV+**	C	H1	H1c	n/a	n/a

Sample ID	Morphology	Chemistry	Lineage	Dataset F1	Dataset F2	Dataset F3	Dataset A1
				mycobiont			photobiont
Peru_CordilleraBlanca_478	**cylindrical and hollow**	**UV-**	C	H1	n/a	n/a	n/a
Peru_CordilleraBlanca_479	**cylindrical and hollow**	**UV-**	C	H1	n/a	n/a	n/a
Peru_CordilleraBlanca_481	**cylindrical and hollow**	**UV-**	C	H1	n/a	n/a	n/a
Peru_Cusco_JQ409343	**cylindrical and hollow**	**UV-**	C	H7	H7	n/a	n/a
Peru_Cusco_JQ409349	**cylindrical and hollow**	**UV+**	C	H1	n/a	n/a	n/a
Peru_Huaraz_394.2	**flat and wide**	**UV+**	C	H1	n/a	n/a	n/a
Peru_Laguna69_369	**cylindrical and hollow**	**nd**	C	H1	H1h	n/a	n/a
Peru_Laguna69_370	**cylindrical and hollow**	**nd**	C	H1	H1g	n/a	n/a
Peru_Laguna69_371	**cylindrical and hollow**	**nd**	C	H1	n/a	n/a	n/a
Peru_Laguna69_372	**cylindrical and hollow**	**nd**	C	H1	n/a	n/a	n/a
Peru_Laguna69_373	**cylindrical and hollow**	**UV-**	C	H1	n/a	n/a	n/a
Peru_Laguna69_464	**cylindrical and hollow**	**UV-**	C	H1	n/a	n/a	n/a
Peru_Laguna69_465	**cylindrical and hollow**	**UV-**	C	H1	n/a	n/a	n/a
Peru_Laguna69_466	**cylindrical and hollow**	**UV-**	C	H1	n/a	n/a	n/a
Peru_LagunaSafunaBaja_482	**cylindrical and hollow**	**UV-**	C	H1	n/a	n/a	n/a
Peru_LagunaSafunaBaja_484	**flat and wide**	**UV-**	C	H1	n/a	n/a	n/a
Peru_LagunaSafunaBaja_485	**cylindrical and hollow**	**UV-**	C	H1	n/a	n/a	n/a
Peru_Langoni_497	**cylindrical and hollow**	**UV-**	C	H1	n/a	n/a	n/a
Peru_Langoni_500	**cylindrical and hollow**	**UV-**	C	H1	H1c	n/a	n/a
Peru_Langoni_501	**cylindrical and hollow**	**UV-**	C	H1	n/a	n/a	n/a
Peru_QuebradaMatara_502	**flat and wide**	**UV-**	C	H1	H1c	n/a	n/a
Peru_QuebradaMatara_504	**cylindrical and hollow**	**UV-**	C	H1	H1c	n/a	n/a
Peru_QuebradaMatara_505	**cylindrical and hollow**	**UV-**	C	H1	n/a	n/a	n/a
Peru_Tarma_392.2	**flat and wide**	**UV+**	C	H1	H1f	n/a	n/a
Peru_Tupatupa_507	**cylindrical and hollow**	**UV-**	C	H1	n/a	n/a	n/a
Peru_Tupatupa_508	**cylindrical and hollow**	**UV-**	C	H1	H1c	n/a	n/a
Peru_Tupatupa_511	**cylindrical and hollow**	**UV-**	C	H1	H1c	n/a	n/a
Peru_Yanapaccha_489	**cylindrical and hollow**	**UV-**	C	H1	n/a	n/a	n/a
Peru_Yanapaccha_491	**flat and wide**	**UV-**	C	H1	n/a	n/a	n/a
Poland_TatraMt_301	**cylindrical and hollow**	**UV+**	C	H1	H1q	H1q1	simplex 1
Poland_TatraMt_303	**cylindrical and hollow**	**UV+**	C	H1	H1q	H1q2	simplex 1
Poland_TatraMt_304	**cylindrical and hollow**	**UV+**	C	H1	H1q	H1q1	simplex 1
Poland_TatraMt_305	**cylindrical and hollow**	**UV+**	C	H5	n/a	n/a	simplex 1
Poland_TatraMt_306	**cylindrical and hollow**	**UV-**	B	H8	H8b	H8b2	simplex 1
Romania_BucegiMt_02	**cylindrical and hollow**	**UV+**	C	H1	H1b	H1b3	vagua
Romania_BucegiMt_45	**cylindrical and hollow**	**UV+**	C	H1	H1b	H1b3	vagua

Sample ID	Morphology	Chemistry	Lineage	Dataset F1	Dataset F2	Dataset F3	Dataset A1
				mycobiont			photobiont
Romania_BucegiMt_53	**cylindrical and hollow**	**UV+**	C	H1	H1b	H1b3	vagua
Romania_BucegiMt_97	**cylindrical and hollow**	**UV+**	C	H1	H1b	H1b3	vagua
Romania_BucegiMt_239.1	**cylindrical and hollow**	**UV+**	C	H1	H1b	H1b3	n/a
Romania_BucegiMt_239.2	**cylindrical and hollow**	**UV-**	C	H1	H1b	H1b3	n/a
Romania_RetezatMt_435	**cylindrical and hollow**	**UV+**	C	H1	n/a	n/a	n/a
Romania_RetezatMt_449	**cylindrical and hollow**	**UV+**	C	H1	n/a	n/a	n/a
Romania_RetezatMt_463	**cylindrical and hollow**	**UV-**	B	H8	H8a	n/a	n/a
Russia_ LitovkaMt_311	**cylindrical and hollow**	**UV-**	C	H1	H1b	H1b6	simplex 2
Russia_ MalayaKhatiparaMt_327	**cylindrical and hollow**	**UV+**	C	H6	H6	H6b	impressa
Russia_ MalayaKhatiparaMt_328	**cylindrical and hollow**	**UV+**	C	H6	H6	H6a	impressa
Russia_AltaiMt_338	**cylindrical and hollow**	**UV+**	C	H4	H4	H4	impressa
Altai_AltaiMt_339	**cylindrical and hollow**	**UV+**	C	H1	H1b	H1b2	impressa
Russia_ArbatMt_342	**cylindrical and hollow**	**UV-**	C	H1	H1i	n/a	simplex 2
Russia_Dzuletta_310	**cylindrical and hollow**	**UV-**	C	H1	H1d	H1d2	simplex 2
Russia_FranzJosef_320	**cylindrical and hollow**	**UV+**	C	H1	H1q	H1q3	simplex 2
Russia_FranzJosef_321	**cylindrical and hollow**	**UV+**	C	H1	H1j	H1j	simplex 2
Russia_FranzJosef_323	**cylindrical and hollow**	**UV+**	A	H9	H9c	H9c	simplex 2
Russia_FranzJosef_324	**cylindrical and hollow**	**UV+**	C	H1	H1q	H1q1	simplex 2
Russia_Gydan_331	**cylindrical and hollow**	**UV+**	A	H9	H9b	H9b	simplex 2
Russia_Gydan_332	**cylindrical and hollow**	**UV+**	A	H10	H10	H10	simplex 2
Russia_Kolguev_315	**cylindrical and hollow**	**UV+**	A	H10	H10	H10	simplex 2
Russia_Kolguev_316	**cylindrical and hollow**	**UV+**	A	H10	H10	H10	simplex 2
Russia_SayanMt_326	**cylindrical and hollow**	**UV+**	C	H1	H1b	H1b3	impressa
Russia_Ural_284.1	**cylindrical and hollow**	**UV+**	C	H1	H1b	H1b1	simplex 2
Russia_Ural_284.2	**cylindrical and hollow**	**UV-**	C	H1	H1d	H1d1	simplex 2
Russia_Yamal_333	**cylindrical and hollow**	**UV-**	C	H1	H1d	H1d1	simplex 1
Russia_Yamal_334	**cylindrical and hollow**	**UV+**	C	H1	H1b	H1b1	simplex 2
Russia_Yamal_335	**cylindrical and hollow**	**UV-**	C	H1	H1d	H1d1	simplex 2
Sweden_Abisko_226	**cylindrical and hollow**	**UV+**	C	H1	H1a	H1a2	simplex 1
Sweden_Abisko_229	**cylindrical and hollow**	**UV-**	C	H1	H1d	H1d1	simplex 2
Sweden_Abisko_234	**cylindrical and hollow**	**UV-**	C	H1	H1d	H1d1	simplex 2
Sweden_Abisko_235	**cylindrical and hollow**	**UV+**	C	H1	H1a	H1a3	simplex 1
Sweden_Gaelivare_72	**cylindrical and hollow**	**UV+**	C	H1	H1q	H1q1	simplex 1
Sweden_Gotland_04	**cylindrical and hollow**	**UV+**	C	H1	H1b	H1b1	simplex 2
Sweden_Gotland_63	**cylindrical and hollow**	**UV+**	C	H1	H1b	H1b1	simplex 2
Sweden_Gotland_64	**cylindrical and hollow**	**UV+**	C	H1	H1b	H1b1	simplex 2

Sample ID	Morphology	Chemistry	Lineage	Dataset F1	Dataset F2	Dataset F3	Dataset A1
			mycobiont				photobiont
Sweden_Gotland_65	**cylindrical and hollow**	**UV+**	C	H1	H1b	H1b1	simplex 2
Sweden_Gotland_71	**cylindrical and hollow**	**UV+**	C	H1	H1b	H1b1	simplex 2
Sweden_Jaemtlands_214.1	**cylindrical and hollow**	**UV+**	C	H1	H1a	H1a2	simplex 1
Sweden_Jaemtlands_214.2	**cylindrical and hollow**	**UV-**	C	H1	H1d	H1d1	simplex 2
Sweden_Jaemtlands_216.1	**cylindrical and hollow**	**UV-**	C	H1	H1d	H1d1	simplex 2
Sweden_Jaemtlands_216.2	**cylindrical and hollow**	**UV+**	A	H9	H9c	H9c	simplex 1
Sweden_Jaemtlands_217.1	**cylindrical and hollow**	**UV+**	A	H9	H9d	H9d	simplex 2
Sweden_Jaemtlands_217.2	**cylindrical and hollow**	**UV-**	C	H1	H1a	H1a2	simplex 2
Sweden_MoiRana_05	**cylindrical and hollow**	**UV+**	C	H1	H1a	H1a2	simplex 1
Sweden_MoiRana_37	**cylindrical and hollow**	**UV+**	C	H1	H1a	H1a2	simplex 1
Sweden_MoiRana_38	**cylindrical and hollow**	**UV+**	C	H1	H1a	H1a2	simplex 1
Sweden_MoiRana_39	**cylindrical and hollow**	**UV+**	C	H1	H1a	H1a2	simplex 1
Sweden_MoiRana_44	**cylindrical and hollow**	**UV+**	C	H1	H1a	H1a2	simplex 1
Sweden_Öland_03	**cylindrical and hollow**	**UV+**	C	H1	H1b	H1b1	simplex 2
Sweden_Öland_17	**cylindrical and hollow**	**UV+**	C	H1	H1b	H1b1	simplex 2
Sweden_Öland_18	**cylindrical and hollow**	**UV+**	C	H1	H1r	H1r	simplex 2
Sweden_Öland_19	**cylindrical and hollow**	**UV+**	C	H1	H1b	H1b1	simplex 2
Sweden_Öland_20	**cylindrical and hollow**	**UV+**	C	H1	H1b	H1b1	simplex 2
Sweden_Öland_164	**cylindrical and hollow**	**UV+**	C	H1	H1k	H1k	simplex 2
Sweden_Öland_26	**cylindrical and hollow**	**UV+**	C	H1	H1b	H1b1	simplex 2
Sweden_Öland_28	**cylindrical and hollow**	**UV+**	C	H1	H1k	H1k	simplex 2
Sweden_Öland_29	**cylindrical and hollow**	**UV+**	C	H1	H1b	H1b1	simplex 2
Sweden_Öland_35	**cylindrical and hollow**	**UV+**	C	H1	H1b	H1b1	simplex 2
Sweden_Öland_07	**cylindrical and hollow**	**UV+**	C	H1	H1b	H1b1	simplex 2
Sweden_Öland_08	**cylindrical and hollow**	**UV+**	C	H1	H1b	H1b1	simplex 2
Sweden_Öland_09	**cylindrical and hollow**	**UV+**	C	H1	H1b	H1b1	simplex 2
Sweden_Öland_10	**cylindrical and hollow**	**UV+**	C	H1	H1b	H1b1	simplex 2
Sweden_Öland_16	**cylindrical and hollow**	**UV+**	C	H1	H1b	H1b1	simplex 2
Sweden_Öland_189	**cylindrical and hollow**	**UV+**	C	H1	H1b	H1b1	simplex 2
Sweden_Taerna_219.1	**cylindrical and hollow**	**UV+**	C	H1	H1a	H1a2	simplex 2
Sweden_Taerna_219.2	**cylindrical and hollow**	**UV+**	C	H1	H1a	H1a2	simplex 1 impressa
Switzerland_SchynigePlatteau_433	**cylindrical and hollow**	**UV-**	B	H8	H8a	n/a	n/a
Switzerland_Valais_111	**cylindrical and hollow**	**UV-**	B	H8	H8b	H8b1	simplex 1
Switzerland_Valais_113	**cylindrical and hollow**	**UV+**	C	H1	H1b	H1b1	impressa
Switzerland_Valais_115	**cylindrical and hollow**	**UV-**	B	H8	H8b	H8b1	simplex 1
USA_Aleutian_286	**cylindrical and hollow**	**UV-**	C	H1	H1a	H1a2	simplex 1

Sample ID	Morphology	Chemistry	Lineage	Dataset F1	Dataset F2	Dataset F3	Dataset A1
			mycobiont				photobiont
USA_Aleutian_287.1	**cylindrical and hollow**	**UV-**	C	H1	H1d	H1d1	simplex 1
USA_Aleutian_287.2	**cylindrical and hollow**	**UV+**	C	H1	H1b	H1b5	simplex 1
USA_Aleutian_288	**cylindrical and hollow**	**UV+**	C	H1	H1l	H1l2	simplex 2
USA_Aleutian_291.1	**cylindrical and hollow**	**UV-**	C	H1	H1d	H1d1	simplex 1
USA_Aleutian_291.2	**cylindrical and hollow**	**UV+**	A	H9	H9a	H9a	simplex 1
USA_Aleutian_EU714413	**cylindrical and hollow**	**UV+**	C	H1	n/a	n/a	n/a
USA_Aleutian_EU714414	**cylindrical and hollow**	**UV-**	C	H1	n/a	n/a	n/a
USA_Aleutian_EU714415	**cylindrical and hollow**	**UV-**	C	H1	n/a	n/a	n/a
USA_Aleutian_EU714416	**cylindrical and hollow**	**UV+**	A	H9	n/a	n/a	n/a
USA_Aleutian_EU714417	**cylindrical and hollow**	**UV-**	C	H1	n/a	n/a	n/a
USA_Aleutian_EU714418	**cylindrical and hollow**	**UV+**	A	H9	n/a	n/a	n/a
USA_Aleutian_EU714419	**cylindrical and hollow**	**UV-**	C	H1	n/a	n/a	n/a
USA_Aleutian_EU714420	**cylindrical and hollow**	**UV-**	C	H1	n/a	n/a	n/a
USA_Aleutian_EU714421	**cylindrical and hollow**	**UV+**	C	H1	n/a	n/a	n/a
USA_Aleutian_EU714422	**cylindrical and hollow**	**UV+**	A	H9	n/a	n/a	n/a
USA_Aleutian_EU714423	**cylindrical and hollow**	**UV+**	A	H9	n/a	n/a	n/a
USA_Aleutian_EU714424	**cylindrical and hollow**	**UV+**	C	H1	n/a	n/a	n/a
USA_Aleutian_EU714427	**cylindrical and hollow**	**UV-**	C	H1	n/a	n/a	n/a
USA_Aleutian_EU714428	**cylindrical and hollow**	**UV+**	A	H9	n/a	n/a	n/a
USA_Aleutian_EU714429	**cylindrical and hollow**	**UV-**	C	H1	n/a	n/a	n/a
USA_BritishColombia_272	**cylindrical and hollow**	**UV-**	C	H1	H1d	H1d1	simplex 2
USA_Chugach_JQ409342	**cylindrical and hollow**	**UV+**	C	H1	H1i	n/a	n/a
USA_Chugach_JQ409344	**cylindrical and hollow**	**UV-**	C	H1	H1e	n/a	n/a
USA_FingerMt_82	**cylindrical and hollow**	**UV-**	C	H1	H1d	H1d1	simplex 2
USA_Kinnikinnik_JQ409345	**cylindrical and hollow**	**UV-**	C	H1	H1c	n/a	n/a
USA_Kinnikinnik_JQ409347	**cylindrical and hollow**	**UV+**	C	H1	n/a	n/a	n/a
USA_Kurupa_209.2	**cylindrical and hollow**	**UV+**	C	H1	H1b	H1b1	impressa
USA_RadioTowerMt_277	**cylindrical and hollow**	**UV-**	C	H1	H1d	H1d1	simplex 2
USA_RadioTowerMt_280	**cylindrical and hollow**	**UV-**	C	H1	H1d	H1d1	simplex 2
USA_Selby_210.1	**cylindrical and hollow**	**UV-**	C	H1	H1d	H1d1	simplex 2
USA_Selby_210.2	**cylindrical and hollow**	**UV+**	C	H1	H1a	H1a6	simplex 1
USA_Summit_213	**cylindrical and hollow**	**UV+**	C	H1	H1b	H1b1	simplex 1
USA_Takahula_212	**cylindrical and hollow**	**UV+**	C	H1	H1b	H1b4	simplex 1
USA_Walker_205	**cylindrical and hollow**	**UV+**	C	H1	H1b	H1b1	simplex 1
USA_Wiseman-Antigu_83.1	**cylindrical and hollow**	**UV-**	C	H1	H1d	H1d1	simplex 2
USA_Wiseman-Antigu_83.2	**cylindrical and hollow**	**UV+**	C	H1	H1b	H1b1	simplex 2

Table S8. Detection of the presence of recombination between and within the three lineages of *Thamnolia*. The tests were performed on all the fungal datasets (F1 to F4) whenever possible, and between and within the three lineages (A, B and C); on six independent loci (ITS, IGS, DEAD, EFα, RPB2, β-tub or the concatenated data set.) The recombination events detected with the "Four Gamete Test" (4GT), the minimum number of recombination events (Rm), Index of Association (Ia) and Standardized Index of Association (rD) are in bold. For each analysed dataset the number of haplotypes (#Hapl), alignment length in base pairs (bp) and the number of segregating sites (S) is shown.

**Loci**	**Lineage**	**Analysed dataset**	**# Hapl**	**alignment length (bp)**	**S**	***Recombination tests***			
						4GT	*Rm*	***Ia***	***rD***
ITS1	A	Dataset F1	2	162	1	*0*	*0*	*n/a*	*n/a*
ITS1+ITS2	A	Dataset F2	5	660	8	*0*	*0*	*n/a*	*n/a*
ITS1 + ITS 2	A	Dataset F3	n/a	n/a	n/a	*n/a*	*n/a*	*n/a*	*n/a*
DEAD			n/a	n/a	n/a	*n/a*	*n/a*	*n/a*	*n/a*
EFα			n/a	n/a	n/a	*n/a*	*n/a*	*n/a*	*n/a*
concatanated			5	1328	9	*0*	*0*	*n/a*	*n/a*
ITS	A	Dataset F4	n/a	n/a	n/a	*n/a*	*n/a*	*n/a*	*n/a*
IGS			n/a	n/a	n/a	*n/a*	*n/a*	*n/a*	*n/a*
DEAD			n/a	n/a	n/a	*n/a*	*n/a*	*n/a*	*n/a*
EFα			n/a	n/a	n/a	*n/a*	*n/a*	*n/a*	*n/a*
RPB2			n/a	n/a	n/a	*n/a*	*n/a*	*n/a*	*n/a*
β-tub			n/a	n/a	n/a	*n/a*	*n/a*	*n/a*	*n/a*
concatanated			5	2416	10	*0*	*0*	***-0.043 p=0.82***	***-0.022 p=0.802***
ITS1	B	Dataset F1	1	163	n/a	*n/a*	*n/a*	*n/a*	*n/a*
ITS1+ITS2	B	Dataset F2	2	660	1	*0*	*0*	*n/a*	*n/a*
ITS1 + ITS 2	B	Dataset F3	n/a	n/a	n/a	*n/a*	*n/a*	*n/a*	*n/a*
DEAD			n/a	n/a	n/a	*n/a*	*n/a*	*n/a*	*n/a*
EFα			n/a	n/a	n/a	*n/a*	*n/a*	*n/a*	*n/a*
concatanated			2	1328	1	*0*	*0*	*n/a*	*n/a*
ITS			n/a	n/a	n/a	*n/a*	*n/a*	*n/a*	*n/a*
IGS			n/a	n/a	n/a	*n/a*	*n/a*	*n/a*	*n/a*

Loci	Lineage	Analysed dataset	# Hapl	alignment length (bp)	S	Recombination tests			
						4GT	*Rm*	***Ia***	***rD***
DEAD	B	Dataset F4	n/a	n/a	n/a	*n/a*	*n/a*	*n/a*	*n/a*
EFα			n/a	n/a	n/a	*n/a*	*n/a*	*n/a*	*n/a*
RPB2			n/a	n/a	n/a	*n/a*	*n/a*	*n/a*	*n/a*
β-tub			n/a	n/a	n/a	*n/a*	*n/a*	*n/a*	*n/a*
concatanated			2	2417	2	*0*	*0*	*n/a*	*n/a*
ITS1	C	Dataset F1	8	165	5	*0*	*0*	*n/a*	*n/a*
ITS1+ITS2	C	Dataset F2	25	666	20	*1*	*1*	*n/a*	*n/a*
ITS	C	Dataset F3	18	667	16	*0*	*0*	*n/a*	*n/a*
DEAD			6	308	5	*0*	*0*	*n/a*	*n/a*
EFα			5	371	5	*0*	*0*	*n/a*	*n/a*
concatanated			33	1332	26	***14***	***2***	***-0.025 p=0.726***	***-0.0141 p=0.689***
ITS	C	Dataset F4	7	677	7	*0*	*0*	*n/a*	*n/a*
IGS			5	207	4	*1*	*1*	*n/a*	*n/a*
DEAD			5	358	4	*0*	*0*	*n/a*	*n/a*
EFα			4	371	4	*0*	*0*	*n/a*	*n/a*
RPB2			4	442	3	*0*	*0*	*n/a*	*n/a*
β-tub			3	368	2	*0*	*0*	*n/a*	*n/a*
concatanated			14	2420	24	***15***	***5***	*0.905 *p=0.002*	*0.182 *p=0.002*
ITS1	ABC	Dataset F1	12	164	9	*3*	*2*	*n/a*	*n/a*
ITS1+ITS2	ABC	Dataset F2	32	666	39	*37*	*6*	*n/a*	*n/a*
ITS1 + ITS 2	ABC	Dataset F3	24	666	12	*12*	*3*	*n/a*	*n/a*
DEAD			10	308	14	*0*	*0*	*n/a*	*n/a*
EFα			6	371	6	*0*	*0*	*n/a*	*n/a*
concatanated			40	1334	56	***33***	***5***	*0.127 *p=0.0059*	*0.071 *p=0.0069*

Loci	Lineage	Analysed dataset	# Hapl	alignment length (bp)	S	Recombination tests			
						4GT	*Rm*	***Ia***	***rD***
ITS	ABC	Dataset F4	12	677	26	*12*	*3*	*n/a*	*n/a*
IGS			9	207	9	*1*	*1*	*n/a*	*n/a*
DEAD			9	358	16	*0*	*0*	*n/a*	*n/a*
EFα			5	371	5	*0*	*0*	*n/a*	*n/a*
RPB2			4	442	3	*0*	*0*	*n/a*	*n/a*
β-tub			5	368	5	*0*	*0*	*n/a*	*n/a*
concatanated			21	2423	64	***48***	***9***	*1.31 p*=0.0009*	*0.273 p*=0.0009*

n/a= not applicable;

* The Bonferroni corrected p-values were significantly different from zero at $p \leq 0.008$ for Dataset F3 and $p \leq 0.002$ for Dataset F4;
ITS1 = Internal transcribed spacer 1; ITS2 = Internal transcribed spacer 2; IGS = Intragenic transcribed spacer; DEAD = Dead-box helicase; EFα = elongation factor alpha; RPB2 = RNA polymerase II second largest subunit; β-tub = beta tubulin.

Table S9a. The variable sites showing the sites with four gametes (4GT) and the minimum number of recombination events (Rm) between the three lineages in the mycobiont Dataset F1.

Lineage	Haplotype number	variable sites											
		1	44	48	51	63	72	107	108	110	111	119	148
		**ITS1**											
**C**	10	G	T	G	C	T	C	A	A	A	T	T	T
**C**	7	A	T	G	T	T	C	A	A	A	T	T	T
**C**	8	A	T	G	C	T	C	A	A	A	T	T	C
**C**	9	A	T	A	C	T	C	A	A	A	T	T	T
**C**	12	A	T	G	C	T	T	A	A	A	T	T	T
**C**	5	A	T	G	C	T	C	A	A	A	T	T	T
**C**	11	A	T	G	C	T	C	A	A	A	T	T	T
**C**	6	A	T	G	C	T	C	A	A	A	T	T	T
**B**	3	A	T	G	C	A	C	-	-	G	C	-	T
**A**	2	A	C	G	C	A	T	-	-	G	T	-	T
**A**	4	A	C	G	C	A	C	-	-	G	T	-	T
**A**	1	A	C	G	C	A	C	-	-	G	T	-	T
Dataset		**F1**											
Rm						63	72						
							72			110			

sites with 4GT: (44,72) (63,72) (72,110)

Table S9b. The variable sites showing the sites with four gametes (4GT) and the minimum number of recombination events (Rm) between the three lineages in the mycobiont Dataset F2.

Lineage	Haplotype number	4	6	14	19	20	36	43	48	77	90	101	113	179	236	261	262	301	302	317	325	368	375	387	397	432	433	435	436	444	474	550	568	581	583	626	628	629	637	639	644	646	649	657	660	663	665
		ITS1 + ITS 2																																													
**C**	24	G	C	A	A	A	C	T	A	G	T	C	T	G	C	C	C	-	-	A	A	T	C	T	C	A	A	A	T	T	T	T	T	G	G	C	G	T	C	C	C	C	C	C	T	A	C
**C**	27	G	C	A	A	A	C	T	G	G	T	C	T	G	C	C	C	-	-	A	A	T	C	T	C	A	A	A	T	T	T	T	T	G	G	C	A	T	C	C	C	C	C	C	T	A	C
**C**	10	G	C	A	-	A	C	T	G	G	T	C	T	G	A	G	C	-	-	A	A	T	C	T	C	A	A	A	T	T	T	T	T	G	G	C	G	T	C	C	C	T	C	C	C	A	T
**C**	19	G	C	A	-	A	C	T	G	G	T	C	T	G	C	C	C	-	-	A	A	T	C	T	C	A	A	A	T	T	T	T	T	G	A	C	G	T	C	C	T	C	C	C	C	A	C
**C**	28	G	C	A	-	A	C	T	G	G	T	C	T	G	C	C	C	-	-	A	A	T	C	T	C	A	A	A	T	T	T	T	T	G	G	C	G	T	G	C	T	C	C	C	C	A	C
**C**	14	G	C	A	-	A	C	T	G	G	T	C	T	G	C	C	C	-	-	A	A	T	C	T	C	A	A	A	T	T	T	T	T	G	G	C	G	T	C	C	T	C	C	C	C	A	C
**C**	11	G	C	A	-	A	C	T	G	G	T	C	T	G	A	C	C	-	-	A	A	T	C	T	C	A	A	A	T	T	T	T	C	G	G	C	G	T	C	C	C	C	C	C	C	A	C
**C**	31	G	C	A	-	A	C	T	G	G	T	C	T	G	A	C	C	-	-	A	A	T	C	T	C	A	A	A	T	T	T	T	C	G	G	C	G	T	C	C	C	C	C	C	C	A	T
**C**	13	G	C	A	-	A	C	T	G	G	T	C	T	G	A	C	C	-	-	A	A	T	C	T	C	A	A	A	T	T	T	T	T	G	G	C	G	T	C	C	C	C	T	C	C	A	C
**C**	9	G	C	A	-	A	C	T	G	G	T	C	T	G	A	C	C	-	-	A	A	T	C	T	C	A	A	A	T	T	T	T	T	G	G	C	G	T	C	C	C	C	C	C	C	A	C
**C**	17	G	C	A	-	A	C	T	G	G	T	C	T	G	A	C	C	-	-	G	A	T	C	T	C	A	A	A	T	T	T	T	T	G	G	C	G	T	C	C	C	C	C	C	C	A	T
**C**	18	G	C	A	-	A	C	T	G	G	T	C	T	G	A	C	C	-	-	A	A	T	C	T	C	A	A	A	T	T	T	C	T	G	G	C	G	T	C	C	C	C	C	C	C	A	T
**C**	32	G	C	A	-	A	C	T	G	G	T	C	T	G	A	C	C	-	-	A	A	T	C	T	T	A	A	A	T	T	T	T	T	G	G	C	G	T	C	C	C	C	C	C	C	A	T
**C**	26	G	C	A	-	A	C	T	G	G	T	C	T	G	-	C	C	-	-	A	A	T	C	T	C	A	A	A	T	T	T	T	T	G	G	C	G	T	C	C	C	C	C	C	C	A	T
**C**	12	G	C	A	-	A	C	T	G	G	T	C	T	G	A	C	C	-	-	A	A	T	C	T	C	A	A	A	T	T	T	T	T	G	G	C	G	T	C	C	C	C	C	C	C	A	T
**C**	20	G	C	A	-	A	C	T	G	G	T	C	T	G	C	C	C	-	-	A	A	T	C	T	C	A	A	A	T	T	T	T	T	G	G	C	G	T	C	C	C	C	C	C	C	A	T
**C**	15	G	C	A	-	A	C	T	G	G	T	C	T	G	C	C	C	-	-	A	A	T	T	T	C	A	A	A	T	T	T	T	T	G	G	C	G	T	C	C	C	C	C	C	C	A	C
**C**	16	G	C	C	-	A	C	T	G	G	T	C	T	G	C	C	C	-	-	A	A	T	C	T	C	A	A	A	T	T	T	T	T	G	G	C	G	T	C	C	C	C	C	C	C	A	C
**C**	21	G	C	A	-	A	C	T	G	G	T	C	T	G	C	C	C	-	-	A	A	T	C	T	C	A	A	A	T	T	C	T	T	G	G	C	G	T	C	C	C	C	C	C	C	A	C
**C**	22	G	C	A	-	A	C	T	G	G	T	C	T	G	T	C	C	-	-	A	A	T	C	T	C	A	A	A	T	T	T	T	T	G	G	C	G	T	C	C	C	C	C	C	C	A	C
**C**	23	G	C	A	-	A	C	T	G	G	T	C	T	G	C	C	C	-	-	A	A	T	C	T	C	A	A	A	T	T	T	T	T	A	G	C	G	T	C	C	C	C	C	C	C	A	C
**C**	25	G	C	A	-	A	C	T	G	G	T	C	T	G	C	C	C	-	-	A	A	T	C	T	C	A	A	A	T	T	T	T	T	G	G	C	G	T	C	T	C	C	C	C	C	A	C
**C**	29	G	C	A	-	A	C	T	G	G	T	C	T	G	C	C	C	-	-	A	G	T	C	T	C	A	A	A	T	T	T	T	T	G	G	C	G	T	C	C	C	C	C	C	C	A	C
**C**	30	G	C	A	-	A	C	T	G	G	T	C	T	G	C	C	C	A	G	A	G	T	C	T	C	A	A	A	T	T	T	T	T	G	G	C	G	T	C	C	C	C	C	C	C	A	C
**C**	8	G	C	A	-	A	C	T	G	G	T	C	T	G	C	C	C	-	-	A	A	T	C	T	C	A	A	A	T	T	T	T	T	G	G	C	G	T	C	C	C	C	C	C	C	A	C
**B**	4	A	C	A	-	G	C	C	G	C	T	T	T	A	A	C	T	-	-	A	A	T	C	A	C	-	-	G	C	-	T	T	T	G	G	T	G	T	C	C	C	C	C	C	C	G	C
**B**	6	A	C	A	-	G	C	C	G	C	T	T	T	A	A	C	T	-	-	A	A	T	C	A	C	-	-	G	C	-	T	T	T	G	G	T	G	T	C	C	C	C	C	C	C	G	T
**A**	2	G	T	A	-	A	T	C	G	C	T	T	T	A	G	C	C	-	-	A	A	C	C	A	T	-	-	G	T	-	T	T	T	G	G	T	G	T	C	T	C	C	C	C	C	A	C
**A**	1	G	T	A	-	A	T	C	G	C	T	T	C	A	G	C	C	-	-	A	A	C	C	A	C	-	-	G	T	-	T	T	T	G	G	T	G	T	C	C	C	C	C	C	C	A	C
**A**	3	G	T	A	-	A	C	C	G	C	T	T	T	A	G	C	C	-	-	A	A	C	C	A	C	-	-	G	T	-	T	T	T	G	G	T	G	C	C	C	C	C	C	C	C	A	C
**A**	5	G	T	A	-	A	C	C	G	C	G	T	T	A	G	C	T	-	-	A	A	C	C	A	C	-	-	G	T	-	T	T	T	G	G	T	G	T	C	C	C	C	C	C	C	A	C
**A**	7	G	T	A	-	A	C	C	G	C	G	T	T	A	G	C	T	-	-	A	A	C	C	A	C	-	-	G	T	-	T	T	T	G	G	T	G	T	C	C	C	C	C	T	C	A	C

Dataset	F2
Rm	6 – 262
	262 – 368
	387 – 397
	397 – 435
	626 – 639
	663 – 665

sites with 4GT: (4,665) (6,262) (6,397) (6,639) (20,665) (36,397) (36,639) (43,397) (43,639) (43,665) (77,397) (77,639) (77,665) (101,397) (101,639) (101,665) (179,397) (179,639) (179,665) (262,368) (262,665) (368,397) (368,639) (387,397) (387,639) (387,665) (397,435) (397,626) (397,639) (397,665) (435,639) (435,665) (436,665) (568,665) (626,639) (626,665) (663,665)

Table S9c. The variable sites showing the sites with four gametes (4GT) and the minimum number of recombination events (Rm) between the three lineages in the mycobiont Dataset F3.

Lineage	Haplotype number	2	4	12	17	18	34	41	46	75	89	99	111	177	234	260	299	300	323	366	373	385	395	430	431	433	434	421	472	566	579	581	624	626	627	635	637	641	644	647	655	658	661	667	705	718	723	776	796	807	808	828	847	854	911	965	966	1022	1088	1091	1094	1193	1241	1334
		ITS																																									DEAD														Efα						
C	24	G	C	A	-	A	C	T	G	G	T	C	T	G	C	C	-	-	A	T	C	T	C	A	A	A	T	T	T	T	G	A	C	G	T	C	C	T	C	C	C	C	A	C	T	A	C	G	G	C	G	A	C	C	A	G	T	G	T	G	A	G	G	G
C	31	G	C	A	-	A	C	T	G	G	T	C	T	G	C	C	-	-	A	T	C	T	C	A	A	A	T	T	T	T	G	A	C	G	T	C	C	T	C	C	C	C	A	C	T	A	C	G	G	C	G	A	C	T	A	G	T	G	T	G	A	G	G	G
C	35	G	C	A	-	A	C	T	G	G	T	C	T	G	C	C	-	-	A	T	C	T	C	A	A	A	T	T	T	T	G	G	C	G	T	G	C	T	C	C	C	C	A	C	T	A	C	G	G	C	G	A	C	C	A	G	T	G	T	G	A	G	G	G
C	26	G	C	A	-	A	C	T	G	G	T	C	T	G	T	C	-	-	A	T	C	T	C	A	A	A	T	T	T	T	G	G	C	G	T	C	C	C	C	C	C	C	A	C	T	A	C	G	G	C	G	A	C	C	A	G	T	A	C	G	A	G	G	G
C	17	G	C	A	-	A	C	T	G	G	T	C	T	G	C	C	-	-	A	T	C	T	C	A	A	A	T	T	T	T	G	G	C	G	T	C	C	T	C	C	C	C	A	C	T	A	C	G	G	C	G	A	C	C	A	G	T	A	T	G	A	G	G	G
C	34	G	C	A	-	A	C	T	G	G	T	C	T	G	C	C	-	-	A	T	C	T	C	A	A	A	T	T	T	T	G	G	C	G	T	C	C	T	C	C	C	C	A	C	T	A	C	G	G	C	G	A	C	T	A	G	T	A	C	G	A	G	G	G
C	21	G	C	A	-	A	C	T	G	G	T	C	T	G	C	C	-	-	A	T	C	T	C	A	A	A	T	T	T	T	G	G	C	G	T	C	C	T	C	C	C	C	A	C	T	A	C	G	G	C	G	A	C	C	A	G	T	A	C	G	A	G	G	G
C	28	G	C	A	A	A	C	T	A	G	T	C	T	G	C	C	-	-	A	T	C	T	C	A	A	A	T	T	T	T	G	G	C	G	T	C	C	C	C	C	C	T	A	C	T	G	C	G	G	T	G	A	C	C	A	G	T	G	T	G	A	G	G	G
C	32	G	C	A	A	A	C	T	G	G	T	C	T	G	C	C	-	-	A	T	C	T	C	A	A	A	T	T	T	T	G	G	C	A	T	C	C	C	C	C	C	T	A	C	T	G	C	G	G	T	G	A	C	C	A	G	T	G	T	G	A	G	G	G
C	40	G	C	A	-	A	C	T	G	G	T	C	T	G	A	C	-	-	A	T	C	T	C	A	A	A	T	T	T	T	G	G	C	G	T	C	C	C	C	C	C	C	A	C	T	G	C	G	G	C	G	A	C	C	A	G	C	G	T	G	G	A	G	-
C	11	G	C	A	-	A	C	T	G	G	T	C	T	G	A	C	-	-	A	T	C	T	C	A	A	A	T	T	T	T	G	G	C	G	T	C	C	C	C	C	C	C	A	C	T	G	C	G	G	C	G	A	C	C	A	G	T	G	T	G	G	A	G	G
C	16	G	C	A	-	A	C	T	G	G	T	C	T	G	A	C	-	-	A	T	C	T	C	A	A	A	T	T	T	T	G	G	C	G	T	C	C	C	C	T	C	C	A	C	T	G	C	G	G	C	G	A	C	C	A	G	T	G	T	G	G	A	G	G
C	37	G	C	A	-	A	C	T	G	G	T	C	T	G	C	C	-	-	G	T	C	T	C	A	A	A	T	T	T	T	G	G	C	G	T	C	C	C	C	C	C	C	A	C	T	G	C	G	G	T	G	A	C	C	A	G	T	G	T	A	A	G	G	G
C	27	G	C	A	-	A	C	T	G	G	T	C	T	G	C	C	-	-	A	T	C	T	C	A	A	A	T	T	T	T	A	G	C	G	T	C	C	C	C	C	C	C	A	C	T	A	C	G	G	C	G	A	C	C	A	G	T	G	T	G	A	G	G	G
C	13	G	C	A	-	A	C	T	G	G	T	C	T	G	C	C	-	-	A	T	C	T	C	A	A	A	T	T	T	T	G	G	C	G	T	C	C	C	C	C	C	C	A	C	T	A	C	G	G	C	G	A	C	C	A	G	T	G	T	G	A	G	G	G
C	9	G	C	A	-	A	C	T	G	G	T	C	T	G	A	C	-	-	A	T	C	T	C	A	A	A	T	T	T	T	G	G	C	G	T	C	C	C	C	C	C	C	A	C	T	A	C	G	G	C	G	A	C	C	A	G	T	G	T	A	A	G	G	G
C	39	G	C	A	-	A	C	T	G	G	T	C	T	G	A	C	-	-	A	T	C	T	C	A	A	A	T	T	T	T	G	G	C	G	T	C	C	C	C	C	C	C	A	C	T	A	C	G	G	C	G	A	C	C	A	G	T	A	T	G	A	G	G	G
C	12	G	C	A	-	A	C	T	G	G	T	C	T	G	A	C	-	-	A	T	C	T	C	A	A	A	T	T	T	T	G	G	C	G	T	C	C	C	C	C	C	C	A	C	T	A	C	G	G	C	G	A	C	C	A	G	T	G	T	G	A	G	G	G
C	23	G	C	A	-	A	C	T	G	G	T	C	T	G	A	C	-	-	A	T	C	T	C	A	A	A	T	T	T	C	G	G	C	G	T	C	C	C	C	C	C	C	A	C	T	G	C	G	G	C	G	A	C	C	A	G	T	A	T	G	A	G	G	G
C	10	G	C	A	-	A	C	T	G	G	T	C	T	G	A	C	-	-	A	T	C	T	C	A	A	A	T	T	T	C	G	G	C	G	T	C	C	C	C	C	C	C	A	C	T	G	C	G	G	C	G	A	C	C	A	G	T	G	T	G	A	G	G	G
C	14	G	C	A	-	A	C	T	G	G	T	C	T	G	A	C	-	-	A	T	C	T	C	A	A	A	T	T	T	T	G	G	C	G	T	C	C	C	C	T	C	C	A	C	T	G	C	G	G	C	G	A	C	C	A	G	T	G	T	G	A	G	G	G
C	20	G	C	A	-	A	C	T	G	G	T	C	T	G	A	C	-	-	A	T	C	T	C	A	A	A	T	T	T	T	G	G	C	G	T	C	C	C	C	C	C	C	A	C	T	G	C	G	G	C	G	A	C	C	A	G	T	G	T	G	A	G	G	G
C	18	G	C	A	-	A	C	T	G	G	T	C	T	G	C	C	-	-	A	T	T	T	C	A	A	A	T	T	T	T	G	G	C	G	T	C	C	C	C	C	C	C	A	C	T	G	C	G	G	T	G	A	C	C	A	G	T	G	T	G	A	G	G	G
C	19	G	C	C	-	A	C	T	G	G	T	C	T	G	C	C	-	-	A	T	C	T	C	A	A	A	T	T	T	T	G	G	C	G	T	C	C	C	C	C	C	C	A	C	T	G	C	G	G	T	G	A	C	C	A	G	T	G	T	G	A	G	G	G
C	25	G	C	A	-	A	C	T	G	G	T	C	T	G	C	C	-	-	A	T	C	T	C	A	A	A	T	T	C	T	G	G	C	G	T	C	C	C	C	C	C	C	A	C	T	G	C	G	G	T	G	A	C	C	A	G	T	G	T	G	A	G	G	G
C	15	G	C	A	-	A	C	T	G	G	T	C	T	G	C	C	-	-	A	T	C	T	C	A	A	A	T	T	T	T	G	G	C	G	T	C	C	C	C	C	C	C	A	C	T	G	C	G	G	T	G	A	C	C	A	G	T	G	T	G	A	G	G	G
C	36	G	C	A	-	A	C	T	G	G	T	C	T	G	C	C	-	-	A	T	C	T	C	A	A	A	T	T	T	T	G	G	C	G	T	C	C	C	C	C	C	C	A	C	T	G	C	G	G	C	G	A	C	C	A	G	T	A	T	G	A	G	G	G
C	38	G	C	A	-	A	C	T	G	G	T	C	T	G	C	C	A	G	G	T	C	T	C	A	A	A	T	T	T	T	G	G	C	G	T	C	C	C	C	C	C	C	A	C	T	G	C	G	G	T	G	A	C	C	A	G	T	A	T	G	A	G	G	G
C	22	G	C	A	-	A	C	T	G	G	T	C	T	G	C	C	-	-	A	T	C	T	C	A	A	A	T	T	T	T	G	G	C	G	T	C	C	C	C	C	C	C	A	C	T	G	C	G	G	T	G	A	C	C	A	G	T	A	T	G	A	G	G	G
C	29	G	C	A	-	A	C	T	G	G	T	C	T	G	C	C	-	-	A	T	C	T	C	A	A	A	T	T	T	T	G	G	C	G	T	C	T	C	C	C	C	C	A	C	T	G	C	G	G	C	G	A	C	C	A	G	T	G	T	G	A	G	G	G
C	30	G	C	A	-	A	C	T	G	G	T	C	T	G	C	C	-	-	A	T	C	T	C	A	A	A	T	T	T	T	G	G	C	G	T	C	C	C	C	C	C	C	A	C	T	G	C	C	G	C	G	A	C	C	A	G	T	G	T	G	A	G	G	G
C	33	G	C	A	-	A	C	T	G	G	T	C	T	G	C	C	-	-	A	T	C	T	C	A	A	A	T	T	T	T	G	G	C	G	T	C	C	C	G	C	C	C	A	C	T	G	C	G	G	C	G	A	C	C	A	G	T	G	T	G	A	G	G	G
C	8	G	C	A	-	A	C	T	G	G	T	C	T	G	C	C	-	-	A	T	C	T	C	A	A	A	T	T	T	T	G	G	C	G	T	C	C	C	C	C	C	C	A	C	T	G	C	G	G	C	G	A	C	C	A	G	T	G	T	G	A	G	G	G
B	4	A	C	A	-	G	C	C	G	C	T	T	T	A	A	T	-	-	A	T	C	A	C	-	-	G	C	-	T	T	G	G	T	G	T	C	C	C	C	C	C	C	G	T	C	G	C	G	G	C	A	G	T	C	A	A	T	G	T	G	G	G	A	G
B	7	A	C	A	-	G	C	C	G	C	T	T	T	A	A	T	-	-	A	T	C	A	C	-	-	G	C	-	T	T	G	G	T	G	T	C	C	C	C	C	C	C	G	T	C	G	C	G	G	C	A	G	T	C	G	A	T	G	T	G	G	G	A	G
A	1	G	T	A	-	A	T	C	G	C	T	T	C	A	G	C	-	-	A	C	C	A	C	-	-	G	T	-	T	T	G	G	T	G	T	C	C	C	C	C	C	C	A	C	C	G	C	G	A	C	A	G	C	C	A	G	T	G	T	G	G	G	A	G
A	2	G	T	A	-	A	T	C	G	C	T	T	T	A	G	C	-	-	A	C	C	A	T	-	-	G	T	-	T	T	G	G	T	G	T	C	T	C	C	C	C	C	A	C	C	G	C	G	A	C	A	G	C	C	A	G	T	G	T	G	G	G	A	G
A	3	G	T	A	-	A	C	C	G	C	T	T	T	A	G	C	-	-	A	C	C	A	C	-	-	G	T	-	T	T	G	G	T	G	C	C	C	C	C	C	C	C	A	C	C	G	T	G	A	C	A	G	C	C	A	G	T	G	T	G	G	G	A	G
A	5	G	T	A	-	A	C	C	G	C	G	T	T	A	G	T	-	-	A	C	C	A	C	-	-	G	T	-	T	T	G	G	T	G	T	C	C	C	C	C	C	C	A	C	C	G	C	G	A	C	A	G	C	C	A	G	T	G	T	G	G	G	A	G
A	6	G	T	A	-	A	C	C	G	C	G	T	T	A	G	T	-	-	A	C	C	A	C	-	-	G	T	-	T	T	G	G	T	G	T	C	C	C	C	C	T	C	A	C	C	G	C	G	A	C	A	G	C	C	A	G	T	G	T	G	G	G	A	G
Dataset		F3																																																														
			4													260																																																
																260				366																																												
Rm																																	624				637																											
																																					637								705																			
																																																							854				1022						

sites with 4GT: (4,260) (4,637) (34,637) (41,637) (75,637) (99,637) (177,637) (260,366) (260,796) (323,1022) (323,1091) (366,637) (385,637) (433,637) (566,1022) (581,854)(624,637) (637,705) (637,796) (637,808) (637,828) (637,1094) (637,1241) (641,1022) (641,1088)(647,1094) (647,1193) (718,1022) (718,1091) (807,1022) (807,1091) (854,1022) (854,1088)

Table S9d. The variable sites showing the sites with four gametes (4GT) and the minimum number of recombination events (Rm) between the three lineages in the mycobiont Dataset F4.

		variable sites																																																																							
Lineage	Haplotype number	4	6	19	20	36	43	48	77	90	101	113	179	236	262	366	385	395	430	431	433	434	442	566	624	627	637	640	641	643	646	660	663	682	720	733	738	811	822	823	843	862	980	987	999	1000	1004	1015	1029	1086	1152	1158	1257	1305	1409	1420	1490	1498	1620	1978	2203	2215	2219	2230	2240	2268	2342	2356	2357	2383	2388	2408	2422	2423
		**ITS**																																**DEAD**																**Efα**						**β-tub**				**RPB2**			**IGS**											
**C**	21	G	C	-	A	C	T	G	G	T	C	T	G	C	C	T	T	C	A	A	A	T	T	T	C	T	C	-	C	T	C	C	A	C	T	A	C	G	C	G	A	C	G	G	G	T	G	G	C	A	C	A	G	G	G	C	A	T	G	T	A	T	C	T	C	G	C	T	T	C	-	T	C	A
**C**	20	G	C	A	A	C	T	A	G	T	C	T	G	C	C	T	T	C	A	A	A	T	T	T	C	T	C	-	C	C	C	T	A	C	T	G	C	G	T	G	A	C	G	G	G	T	G	G	C	G	T	A	G	G	G	C	A	T	A	T	C	C	C	T	C	G	C	T	C	C	-	C	C	A
**C**	16	G	C	-	A	C	T	G	G	T	C	T	G	C	C	T	T	C	A	A	A	T	T	T	C	T	C	-	C	C	C	C	A	C	T	G	C	G	T	G	A	C	G	G	G	T	G	G	C	A	T	A	G	G	G	C	A	T	A	T	C	T	T	T	C	G	C	T	T	C	-	T	C	A
**C**	17	G	C	-	A	C	T	G	G	T	C	T	G	C	C	T	T	C	A	A	A	T	T	T	C	T	C	-	C	C	C	C	A	C	T	G	C	G	T	G	A	C	G	G	G	T	G	G	C	A	T	A	G	G	G	C	A	T	A	T	C	C	T	T	C	G	C	T	T	C	-	T	C	A
**C**	18	G	C	-	A	C	T	G	G	T	C	T	G	C	C	T	T	C	A	A	A	T	T	T	C	T	T	-	C	C	C	C	A	C	T	G	C	G	C	G	A	C	G	G	G	T	T	G	C	G	T	A	G	G	G	C	A	T	A	C	C	T	T	T	C	G	C	T	T	C	-	C	C	A
**C**	19	G	C	-	A	C	T	G	G	T	C	T	G	C	C	T	T	C	A	A	A	T	T	T	C	T	C	-	C	C	G	C	A	C	T	G	C	G	C	G	A	C	G	G	G	T	T	G	C	G	T	A	G	G	G	C	A	T	A	C	C	T	T	T	C	G	C	T	T	C	-	T	C	A
**C**	12	G	C	-	A	C	T	G	G	T	C	T	G	C	C	T	T	C	A	A	A	T	T	T	C	T	C	-	C	C	C	C	A	C	T	G	C	G	C	G	A	C	G	G	G	T	T	G	C	G	T	A	G	G	G	C	A	T	A	C	C	T	T	T	C	G	C	T	T	C	-	T	C	A
**C**	15	G	C	-	A	C	T	G	G	T	C	T	G	C	C	T	T	C	A	A	A	T	T	T	C	T	C	-	C	C	C	C	A	C	T	G	C	G	C	G	A	C	G	G	G	T	T	G	C	G	T	A	G	G	G	C	A	T	A	T	C	T	T	T	C	G	C	T	T	C	-	T	C	A
**C**	14	G	C	-	A	C	T	G	G	T	C	T	G	C	C	T	T	C	A	A	A	T	T	T	C	T	C	-	C	C	C	C	A	C	T	G	C	G	C	G	A	C	G	G	G	T	G	G	C	G	T	A	G	G	G	C	A	T	A	T	C	T	T	T	C	G	C	T	T	C	-	T	C	A
**C**	11	G	C	-	A	C	T	G	G	T	C	T	G	C	C	T	T	C	A	A	A	T	T	T	C	T	C	-	C	C	C	C	A	C	T	G	C	G	C	G	A	C	G	G	G	T	G	G	C	G	T	A	G	G	G	C	A	T	A	C	C	T	T	T	C	G	C	T	T	C	-	C	C	A
**C**	13	G	C	-	A	C	T	G	G	T	C	T	G	C	C	T	T	C	A	A	A	T	T	T	C	T	C	-	C	C	C	C	A	C	T	G	C	G	C	G	A	C	G	G	G	T	G	G	C	G	T	A	G	G	G	C	A	T	A	C	C	T	T	T	C	G	C	T	T	C	-	T	C	A
**C**	8	G	C	-	A	C	T	G	G	T	C	T	G	A	C	T	T	C	A	A	A	T	T	C	C	T	C	C	C	C	C	C	A	C	T	G	C	G	C	G	A	C	G	G	G	T	G	A	C	G	T	A	G	G	G	C	A	T	A	T	C	C	C	T	C	G	C	T	T	T	-	T	C	A
**C**	9	G	C	-	A	C	T	G	G	T	C	T	G	A	C	T	T	C	A	A	A	T	T	T	C	T	C	C	C	C	C	C	A	C	T	G	C	G	C	G	A	C	G	G	G	T	G	G	C	G	T	G	A	G	G	C	A	C	G	T	C	C	C	T	C	G	C	T	T	C	-	T	C	A
**C**	10	G	C	-	A	C	T	G	G	T	C	T	G	A	C	T	T	C	A	A	A	T	T	T	C	T	C	C	C	C	C	C	A	C	T	G	C	G	C	G	A	C	G	G	G	T	G	G	C	G	T	A	G	G	G	C	A	C	G	T	C	C	C	T	C	G	C	T	T	C	-	T	C	A
**B**	6	A	C	-	G	C	C	G	C	T	T	T	A	A	T	T	A	C	-	-	G	C	-	T	T	T	C	-	-	C	C	C	G	T	C	G	C	G	C	A	G	T	A	C	G	C	G	G	C	G	T	G	G	A	G	A	A	T	G	T	C	T	C	C	T	T	C	A	T	C	T	T	C	A
**B**	7	A	C	-	G	C	C	G	C	T	T	T	A	A	T	T	A	C	-	-	G	C	-	T	T	T	C	-	-	C	C	C	G	T	C	G	C	G	C	A	G	T	A	C	G	C	G	G	C	G	T	G	G	A	A	A	C	T	G	T	C	T	C	C	T	T	C	A	T	C	T	T	C	A
**A**	1	G	T	-	A	T	C	G	C	T	T	T	A	G	C	C	A	T	-	-	G	T	-	T	T	T	T	-	-	C	C	C	A	C	C	G	C	A	C	A	G	C	G	C	G	C	G	G	T	G	T	G	G	A	G	C	A	T	G	T	C	T	C	C	T	T	T	A	T	C	-	T	C	A
**A**	2	G	T	-	A	T	C	G	C	T	T	T	A	G	C	C	A	T	-	-	G	T	-	T	T	T	T	-	-	C	C	C	A	C	C	G	C	A	C	A	G	C	G	C	A	C	G	G	T	G	T	G	G	A	G	C	A	T	G	T	C	T	C	C	T	T	T	A	T	C	-	T	C	A
**A**	5	G	T	-	A	C	C	G	C	G	T	T	A	G	T	C	A	C	-	-	G	T	-	T	T	T	C	-	-	C	C	C	A	C	C	G	C	A	C	A	G	C	G	C	G	C	G	G	T	G	T	G	G	A	G	C	A	T	G	T	C	T	C	C	T	T	C	A	T	C	-	T	-	-
**A**	4	G	T	-	A	C	C	G	C	T	T	T	A	G	C	C	A	C	-	-	G	T	-	T	T	C	C	-	-	C	C	C	A	C	C	G	T	A	C	A	G	C	G	C	G	C	G	G	T	G	T	G	G	A	G	C	A	T	G	T	C	T	C	C	T	T	C	A	T	C	-	T	C	A
**A**	3	G	T	-	A	T	C	G	C	T	T	C	A	G	C	C	A	C	-	-	G	T	-	T	T	T	C	-	-	C	C	C	A	C	C	G	C	A	C	A	G	C	G	C	G	C	G	G	T	G	T	G	G	A	G	C	A	T	G	T	C	T	C	C	T	T	C	A	T	C	-	T	C	A
Dataset		F4																																																																									

Rm

6 262
262 366
624 637
637 720
822 1086
1158 1498
1620 2215
2215 2219
2219 2408

sites with 4GT: (6,262) (6,637) (36,637) (43,637) (77,637) (101,637) (179,637) (262,366) (262,811) (262,1029) (366,637) (385,637) (433,637) (624,637) (637,720) (637,811) (637,823) (637,843) (637,987) (637,1000) (637,1004) (637,1029) (637,1158) (637,1305) (637,1620) (637,1978) (637,2219) (637,2230) (637,2240) (637,2268) (637,2356) (637,2408) (822,1086) (822,2215) (822,2219) (822,2408) (1004,1978) (1004,2408) (1086,1620) (1086,2215) (1086,2219) (1158,1498) (1158,2215) (1620,2215) (1978,2408) (2215,2219) (2215,2408) (2219,2408)

Table S9e. The variable sites showing the sites with four gametes (4GT) and the minimum number of recombination events (Rm) within Lineage C in Dataset F2.

Lineage	Haplotype number	variable sites																							
		14	20	48	236	261	306	307	317	325	375	397	474	550	568	581	583	628	637	639	644	646	649	660	665
		ITS																							
**C**	1	A	-	G	C	C	-	-	A	A	C	C	T	T	T	G	G	G	C	C	C	C	C	C	C
**C**	2	A	-	G	A	C	-	-	A	A	C	C	T	T	T	G	G	G	C	C	C	C	C	C	C
**C**	3	A	-	G	A	G	-	-	A	A	C	C	T	T	T	G	G	G	C	C	C	T	C	C	T
**C**	4	A	-	G	A	C	-	-	A	A	C	C	T	T	C	G	G	G	C	C	C	C	C	C	C
**C**	5	A	-	G	A	C	-	-	A	A	C	C	T	T	T	G	G	G	C	C	C	C	C	C	T
**C**	6	A	-	G	A	C	-	-	A	A	C	C	T	T	T	G	G	G	C	C	C	C	T	C	C
**C**	7	A	-	G	C	C	-	-	A	A	C	C	T	T	T	G	G	G	C	C	T	C	C	C	C
**C**	8	A	-	G	C	C	-	-	A	A	T	C	T	T	T	G	G	G	C	C	C	C	C	C	C
**C**	9	C	-	G	C	C	-	-	A	A	C	C	T	T	T	G	G	G	C	C	C	C	C	C	C
**C**	10	A	-	G	A	C	-	-	G	A	C	C	T	T	T	G	G	G	C	C	C	C	C	C	T
**C**	11	A	-	G	A	C	-	-	A	A	C	C	T	C	T	G	G	G	C	C	C	C	C	C	T
**C**	12	A	-	G	C	C	-	-	A	A	C	C	T	T	T	G	A	G	C	C	T	C	C	C	C
**C**	13	A	-	G	C	C	-	-	A	A	C	C	T	T	T	G	G	G	C	C	C	C	C	C	T
**C**	14	A	-	G	C	C	-	-	A	A	C	C	C	T	T	G	G	G	C	C	C	C	C	C	C
**C**	15	A	-	G	T	C	-	-	A	A	C	C	T	T	T	G	G	G	C	C	C	C	C	C	C
**C**	16	A	-	G	C	C	-	-	A	A	C	C	T	T	T	A	G	G	C	C	C	C	C	C	C
**C**	17	A	A	A	C	C	-	-	A	A	C	C	T	T	T	G	G	G	C	C	C	C	C	T	C
**C**	18	A	-	G	C	C	-	-	A	A	C	C	T	T	T	G	G	G	C	T	C	C	C	C	C
**C**	19	A	-	G	-	C	-	-	A	A	C	C	T	T	T	G	G	G	C	C	C	C	C	C	T
**C**	20	A	A	G	C	C	-	-	A	A	C	C	T	T	T	G	G	A	C	C	C	C	C	T	C
**C**	21	A	-	G	C	C	-	-	A	A	C	C	T	T	T	G	G	G	G	C	T	C	C	C	C
**C**	22	A	-	G	C	C	-	-	A	G	C	C	T	T	T	G	G	G	C	C	C	C	C	C	C
**C**	23	A	-	G	C	C	G	A	A	G	C	C	T	T	T	G	G	G	C	C	C	C	C	C	C
**C**	24	A	-	G	A	C	-	-	A	A	C	C	T	T	C	G	G	G	C	C	C	C	C	C	T
**C**	25	A	-	G	A	C	-	-	A	A	C	T	T	T	T	G	G	G	C	C	C	C	C	C	T
Dataset		F2																							
Rm															568										665

sites with 4GT: (568,665)

Table S9f. The variable sites showing the sites with four gametes (4GT) and the minimum number of recombination events (Rm) within Lineage C in Dataset F3.

Lineage	Haplotype number	variable sites																												
		12	17	46	234	290	300	323	373	472	566	579	581	626	635	637	642	645	648	659	719	777	808	855	967	1023	1089	1092	1095	1194
		**ITS**																			**DEAD**					**Efα**				
**C**	1	A	-	G	C	-	-	A	C	T	T	G	A	G	C	C	T	C	C	C	A	G	C	C	T	G	T	G	A	G
**C**	2	A	-	G	C	-	-	A	C	T	T	G	A	G	C	C	T	C	C	C	A	G	C	T	T	G	T	G	A	G
**C**	3	A	-	G	C	-	-	A	C	T	T	G	G	G	G	C	T	C	C	C	A	G	C	C	T	G	T	G	A	G
**C**	4	A	-	G	T	-	-	A	C	T	T	G	G	G	C	C	C	C	C	C	A	G	C	C	T	A	C	G	A	G
**C**	5	A	-	G	C	-	-	A	C	T	T	G	G	G	C	C	T	C	C	C	A	G	C	C	T	A	T	G	A	G
**C**	6	A	-	G	C	-	-	A	C	T	T	G	G	G	C	C	T	C	C	C	A	G	C	T	T	A	C	G	A	G
**C**	7	A	-	G	C	-	-	A	C	T	T	G	G	G	C	C	T	C	C	C	A	G	C	C	T	A	C	G	A	G
**C**	8	A	A	A	C	-	-	A	C	T	T	G	G	G	C	C	C	C	C	T	G	G	T	C	T	G	T	G	A	G
**C**	9	A	A	G	C	-	-	A	C	T	T	G	G	A	C	C	C	C	C	T	G	G	T	C	T	G	T	G	A	G
**C**	10	A	-	G	A	-	-	A	C	T	T	G	G	G	C	C	C	C	T	C	G	G	C	C	T	G	T	G	G	A
**C**	11	A	-	G	A	-	-	A	C	T	T	G	G	G	C	C	C	C	C	C	G	G	C	C	C	G	T	G	G	A
**C**	12	A	-	G	A	-	-	A	C	T	T	G	G	G	C	C	C	C	C	C	G	G	C	C	T	G	T	G	G	A
**C**	13	A	-	G	C	-	-	G	C	T	T	G	G	G	C	C	C	C	C	C	G	G	T	C	T	G	T	A	A	G
**C**	14	A	-	G	C	-	-	A	C	T	T	A	G	G	C	C	C	C	C	C	A	G	C	C	T	G	T	G	A	G
**C**	15	A	-	G	C	-	-	A	C	T	T	G	G	G	C	C	C	C	C	C	A	G	C	C	T	G	T	G	A	G
**C**	16	A	-	G	A	-	-	A	C	T	T	G	G	G	C	C	C	C	C	C	A	G	C	C	T	G	T	A	A	G
**C**	17	A	-	G	A	-	-	A	C	T	T	G	G	G	C	C	C	C	C	C	A	G	C	C	T	A	T	G	A	G
**C**	18	A	-	G	A	-	-	A	C	T	T	G	G	G	C	C	C	C	C	C	A	G	C	C	T	G	T	G	A	G
**C**	19	A	-	G	A	-	-	A	C	T	C	G	G	G	C	C	C	C	C	C	G	G	C	C	T	A	T	G	A	G
**C**	20	A	-	G	A	-	-	A	C	T	C	G	G	G	C	C	C	C	C	C	G	G	C	C	T	G	T	G	A	G
**C**	21	A	-	G	A	-	-	A	C	T	T	G	G	G	C	C	C	C	T	C	G	G	C	C	T	G	T	G	A	G
**C**	22	A	-	G	A	-	-	A	C	T	T	G	G	G	C	C	C	C	C	C	G	G	C	C	T	G	T	G	A	G
**C**	23	A	-	G	C	-	-	A	T	T	T	G	G	G	C	C	C	C	C	C	G	G	T	C	T	G	T	G	A	G
**C**	24	C	-	G	C	-	-	A	C	T	T	G	G	G	C	C	C	C	C	C	G	G	T	C	T	G	T	G	A	G
**C**	25	A	-	G	C	-	-	A	C	C	T	G	G	G	C	C	C	C	C	C	G	G	T	C	T	G	T	G	A	G
**C**	26	A	-	G	C	-	-	A	C	T	T	G	G	G	C	C	C	C	C	C	G	G	T	C	T	G	T	G	A	G
**C**	27	A	-	G	C	-	-	A	C	T	T	G	G	G	C	C	C	C	C	C	G	G	C	C	T	A	T	G	A	G
**C**	28	A	-	G	C	A	G	G	C	T	T	G	G	G	C	C	C	C	C	C	G	G	T	C	T	A	T	G	A	G
**C**	29	A	-	G	C	-	-	A	C	T	T	G	G	G	C	C	C	C	C	C	G	G	T	C	T	A	T	G	A	G
**C**	30	A	-	G	C	-	-	A	C	T	T	G	G	G	C	T	C	C	C	C	G	G	C	C	T	G	T	G	A	G
**C**	31	A	-	G	C	-	-	A	C	T	T	G	G	G	C	C	C	C	C	C	G	C	C	C	T	G	T	G	A	G
**C**	32	A	-	G	C	-	-	A	C	T	T	G	G	G	C	C	C	G	C	C	G	G	C	C	T	G	T	G	A	G
**C**	33	A	-	G	C	-	-	A	C	T	T	G	G	G	C	C	C	C	C	C	G	G	C	C	T	G	T	G	A	G
Dataset		**F3**																												
Rm													581											855						
																								855		1023				

sites with 4GT: (323,1023) (323,1092) (566,1023) (581,855) (642,1023) (642,1089) (648,1095) (648,1194) (719,1023) (719,1092) (808,1023) (808,1092) (855,1023) (855,1089)

Table S9g. The variable sites showing the sites with four gametes (4GT) and the minimum number of recombination events (Rm) within Lineage C in Dataset F4.

Lineage	Haplotype number	19	48	236	566	637	640	643	646	660	733	822	1004	1015	1086	1152	1158	1257	1498	1620	1978	2203	2215	2219	2357	2383	2407
		**ITS**									**DEAD**				**Efα**				**β-tub**		**RPB2**			**IGS**			
**C**	1	-	G	A	C	C	C	C	C	C	G	C	G	A	G	T	A	G	T	A	T	C	C	C	T	T	T
**C**	2	-	G	A	T	C	C	C	C	C	G	C	G	G	G	T	G	A	C	G	T	C	C	C	T	C	T
**C**	3	-	G	A	T	C	C	C	C	C	G	C	G	G	G	T	A	G	C	G	T	C	C	C	T	C	T
**C**	4	-	G	C	T	C	-	C	C	C	G	C	G	G	G	T	A	G	T	A	C	C	T	T	T	C	C
**C**	5	-	G	C	T	C	-	C	C	C	G	C	T	G	G	T	A	G	T	A	C	C	T	T	T	C	T
**C**	6	-	G	C	T	C	-	C	C	C	G	C	G	G	G	T	A	G	T	A	C	C	T	T	T	C	T
**C**	7	-	G	C	T	C	-	C	C	C	G	C	G	G	G	T	A	G	T	A	T	C	T	T	T	C	T
**C**	8	-	G	C	T	C	-	C	C	C	G	C	T	G	G	T	A	G	T	A	T	C	T	T	T	C	T
**C**	9	-	G	C	T	C	-	C	C	C	G	T	G	G	A	T	A	G	T	A	T	C	T	T	T	C	T
**C**	10	-	G	C	T	C	-	C	C	C	G	T	G	G	A	T	A	G	T	A	T	C	C	T	T	C	T
**C**	11	-	G	C	T	T	-	C	C	C	G	C	T	G	G	T	A	G	T	A	C	C	T	T	T	C	C
**C**	12	-	G	C	T	C	-	C	G	C	G	C	T	G	G	T	A	G	T	A	C	C	T	T	T	C	T
**C**	13	A	A	C	T	C	-	C	C	T	G	T	G	G	G	T	A	G	T	A	T	C	C	C	C	C	C
**C**	14	-	G	C	T	C	-	T	C	C	A	C	G	G	A	C	A	G	T	G	T	A	T	C	T	C	T
Dataset		**F4**																									
Rm												822			1086												
															1086					1620							
																				1620			2215				
																							2215	2219			
																								2219			2407

sites with 4GT: (236,1620) (822,1086) (822,2215) (822,2219) (822,2407) (1004,1978) (1004,2407) (1086,1620) (1086,2215) (1086,2219) (1620,2215) (1978,2407) (2215,2219) (2215,2407) (2219,2407)
